# Practical Guidance for Using PurpleAir Particle Monitors for Indoor and Outdoor Measurements in Community Field Studies

**DOI:** 10.1007/s44408-025-00048-4

**Published:** 2025-08-11

**Authors:** Mingyu Wang, David Chang, Aditya Singh, Jeff Wagner, Zhong-Min Wang, Brett C. Singer, Shelly L. Miller, Nayamin Martinez, Ruben Rodriguez, Isabella Kaser, McKenna Thompson, Mohammad Heidarinejad, Brent Stephens, Gina Solomon

**Affiliations:** 1 Department of Civil, Architectural, and Environmental Engineering, Illinois Institute of Technology, Alumni Memorial Hall Room 228, 3201 S Dearborn Street, Chicago, IL 60616 USA; 2 Tracking California, Public Health Institute, Oakland, CA USA; 3 California Department of Public Health, Center for Laboratory Sciences, Environmental Health Laboratory, Richmond, CA USA; 4 Lawrence Berkley National Laboratory, Berkeley, CA USA; 5 Department of Mechanical Engineering, University of Colorado, Boulder, CO USA; 6 Central California Environmental Justice Network, Fresno, CA USA; 7 Office of Environmental Health Hazard Assessment, California Environmental Protection Agency, Oakland, CA USA; 8 Public Health Institute, Oakland, CA USA; 9 Division of Occupational, Environmental and Climate Medicine, University of California San Francisco School of Medicine, San Francisco, CA USA

**Keywords:** Low-cost particle monitors, Long-term indoor exposure assessments, Performance, Data collection, QA/QC

## Abstract

Low-cost particle monitors have been widely evaluated in laboratory and ambient monitoring settings, but we have less knowledge about their performance for long-term indoor and outdoor monitoring in residential settings. We seek to provide practical guidance for using a type of low-cost particle monitors that have become widespread for indoor and outdoor monitoring in community field studies, PurpleAir PA-II monitors. We base our insights on experiences in a community-led residential field study in and around homes of predominantly agricultural workers in California’s San Joaquin Valley. Our guidance spans three categories: (1) providing tools for handling and merging disparate data structures resulting from Wi-Fi-transmitted data and data collected on onboard microSD cards, (2) assessing performance metrics of PA-II monitors from laboratory co-location and field measurements, and (3) assessing data collection success rates of Wi-Fi data transmission and microSD card data acquisition from our study locations. The post-processing methods we demonstrate can successfully align data from both Wi-Fi transmission and microSD cards. Laboratory co-location measurements demonstrated that > 90% of the tested monitors performed well relative to each other (high precision), with only a few problematic monitors that warranted further investigation or exclusion from use. The application of co-location factors generated using the mean of all co-located monitors as a reference did not significantly affect distributions of field-measured indoor or outdoor PM_2.5_ concentrations. Relying solely on Wi-Fi data transmission in our study would have resulted in large data loss (i.e., < 50% success rate); using microSD card storage with PA-II-SD monitors increased the data collection success rate to over 80% in these settings. This work contributes to the growing body of knowledge on low-cost particle sensor performance and usability.

**Graphical abstract:**

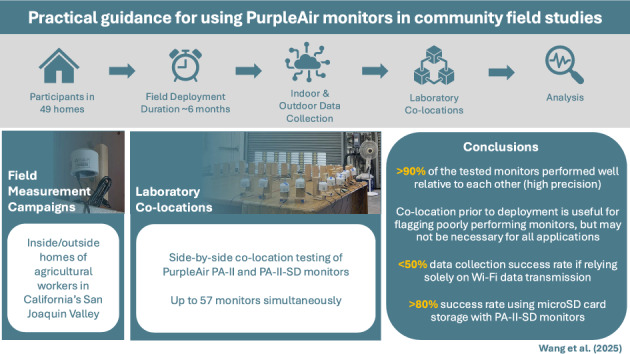

**Supplementary Information:**

The online version contains supplementary material available at 10.1007/s44408-025-00048-4.

## Introduction

There has been a tremendous increase in the use of low-cost particle monitors for monitoring indoor and/or outdoor air quality in the last decade, as sensor technologies have improved in performance, costs have decreased, and software applications to manage data have become more widespread and easier to use (Giordano et al. 2021; Jayaratne et al. 2020; Morawska et al. 2018; Sá et al. 2022). Most consumer-grade low-cost particle monitors use one (or more) of only a few makes and models of optical particle sensors (Zou et al. 2020a, 2020b). One such commercially available family of low-cost consumer-grade particle monitors, PurpleAir, has gained extensive usage because of its high performance relative to regulatory and research-grade monitors and its accessible mapping and visualization features. PurpleAir has sold over 25,000 units and has established a vast global user network (Ashworth 2023), with many monitors set to publicly share indoor or outdoor air quality data for others to view, download, and analyze (PurpleAir 2023a). Numerous studies have found that PurpleAir monitors (and their bare Plantower sensors) perform quite well compared to much more expensive regulatory or research-grade monitors (i.e., R^2^ > 0.9 is common), especially for ambient fine particulate matter (PM_2.5_) and many (but not all) indoor PM_2.5_ sources (Singer and Delp 2018; Levy Zamora et al. 2019; Ardon-Dryer et al. 2020; Li et al. 2020; Tryner et al. 2020; Koehler et al. 2023; Molina Rueda et al. 2023; Park et al. 2023; SCAQMD 2023; Srishti et al. 2023). The PurpleAir community is also active through various online platforms, where users exchange information and support each other with questions. Finally, the PurpleAir support team typically responds quickly to user questions, which enhances the ease in using PurpleAir monitors.

These factors have also led to increased usage of PurpleAir monitors in community monitoring (deSouza and Kinney 2021; Durkin et al. 2020; Mullen et al. 2022) and also research studies. For example, data from existing PurpleAir sensor networks have been used to investigate the impacts of wildfire smoke on indoor PM_2.5_ concentrations (Liang et al. 2021), evaluate the impacts of July 4th fireworks on ambient PM_2.5_ concentrations (Mousavi et al. 2021), quantify indoor PM_2.5_ exposures during COVID-19 closures (Mousavi and Wu 2021), identify elevated ambient PM_2.5_ concentrations from woodsmoke (Robinson et al. 2023), assess the magnitude of ambient PM_2.5_ infiltration in residences (Lunderberg et al. 2023; Wallace et al. 2022a, b), characterize the relative contributions of indoor and outdoor sources to indoor PM_2.5_ concentrations (Wallace and Ott 2023), and assess socioeconomic inequity in indoor and outdoor PM_2.5_ concentrations (Wallace 2024). PurpleAir monitors and/or their bare Plantower sensors have also been deployed in targeted indoor field campaigns to assess secondhand aerosol exposure from vaping marijuana (Wallace et al. 2020), assess the effects of portable air cleaners on ambient PM_2.5_ infiltration during wildfires (Xiang et al. 2021), quantify spatiotemporal transport of indoor PM_2.5_ sources in homes (Sankhyan et al. 2022), assess PM_2.5_ exposure in vulnerable populations (Koehler et al. 2023), evaluate personal PM_2.5_ exposure models (Tsameret et al. 2024), evaluate the impacts of a do-it-yourself (DIY) box fan and filter combination on indoor PM_2.5_ in a smoke-impacted community (Prathibha et al. 2024), compare the impacts of different types of mechanical ventilation systems on indoor PM_2.5_ concentrations in homes (Francisco et al. 2025), and assess the effects of portable air cleaners on concentrations, sources, and sinks of indoor PM_2.5_ in homes of U.S. military Veterans with underlying respiratory disease (Stephens et al. 2025; Farhoodi et al. 2025).

Several studies have also used data from networks of outdoor and/or indoor PurpleAir monitors to evaluate various performance metrics (e.g., accuracy, precision, and bias) of individual PurpleAir monitors and Plantower sensors (Sayahi et al. 2019; Searle et al. 2023; Wallace 2022), explore calibration methods (Datta et al. 2020; deSouza et al. 2022), investigate drift/degradation over time (Collier-Oxandale et al. 2022; deSouza et al. 2023; Fang et al. 2025), propose correction factors and improved algorithms for improving accuracy and reducing bias (Barkjohn et al. 2021; Wallace 2022), and provide practical guidance for using and interpreting data from PurpleAir monitors (Zimmerman 2022; Stampfer et al. 2024).

This work aims to contribute to this growing body of knowledge on PurpleAir performance and usability for residential field studies by summarizing practical guidance based on our recent experiences using PurpleAir PA-II monitors deployed in a community-led, residential field study of indoor air filtration interventions aimed at mitigating wildfire smoke exposure in communities of predominantly agricultural workers: Filtration for Respiratory Exposure to wildfire Smoke from Swamp Cooler Air (FRESSCA) (Solomon et al. 2025). Results of the intervention are presented elsewhere and are not the focus of this manuscript. Here, we aim to provide performance insights, useful information, and tools for improving the likelihood of success in using these low-cost monitors for exposure assessment in other residential field studies of indoor and outdoor air. Our insights and guidance span three main categories: (1) handling and merging disparate data structures resulting from Wi-Fi-transmitted data and data collected on onboard microSD cards from PA-II and PA-II-SD monitors, (2) assessing performance metrics of PA-II monitors from laboratory co-location and field measurements, and (3) assessing the success rates of data collection via Wi-Fi data transmission and microSD card data acquisition from the residential field settings in our study.

## Methodology

Our experiences center around two field measurement campaigns that were conducted in Fresno, Kings, and Kern Counties in California’s San Joaquin Valley (SJV) to test the impacts of different air filtration interventions on indoor particulate matter (PM) concentrations in the homes of predominantly agricultural workers (Fig. S1). A smaller pilot field campaign was first conducted in 2022 (target sample size of 30 homes), and a larger field campaign was conducted in 2023 (target sample size of 50 homes). In both phases, participation was restricted to individuals living in non-smoking homes. Institutional Review Board (IRB) approval for the field study was obtained from the Public Health Institute (PHI) IRB (IRB #I22-002).

### Deploying the PA-II and extracting data via Wi-Fi and microSD cards

**Fig. 1:**
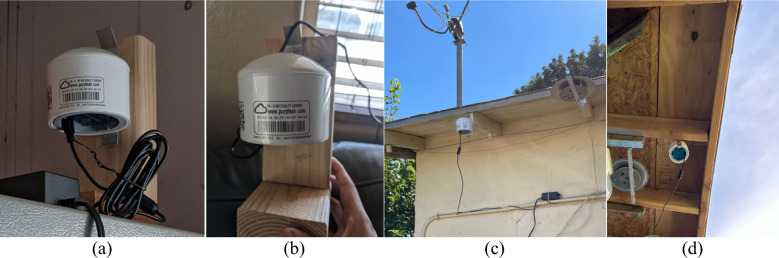
Examples of custom wooden mounting structure for PA-II monitors deployed indoors (**a** and **b**) and outdoors (**c** and **d**) in our residential field study

At the initiation of our study in 2021, three models of PM monitors were commercially available from PurpleAir: PA-I-Indoor, PA-II (Wi-Fi only, without onboard storage), and PA-II-SD (PA-II with Wi-Fi and onboard storage using a microSD card). The PA-I-Indoor monitor contained one Plantower optical particle sensor while the two types of PA-II units contain two. Although our study was designed to focus primarily on indoor measurements, we declined to select the PA-I-Indoor sensor, given that the performance assessments we had reviewed at the time demonstrated much lower correlations with reference instruments than PA-II, indicating that the use of two sensors in parallel in PA-II improves accuracy (SCAQMD 2020). Since the attached mounting structure for the PA-II units is tailored for outdoor applications (i.e., a bracket can be screwed directly into a wall outdoors, but that is not ideal for indoor mounting), we mounted the PA-II units on a small custom wooden structure built from short pieces of 5-cm by 10-cm lumber (Fig. 1). Figure 1a,b show two examples of indoor monitor installations and Fig. 1c,d show two examples of outdoor installations.

Data can be stored or extracted from PA-II monitors in different ways depending on the model of monitor. The PA-II monitor does not have any onboard storage and can only transmit data via Wi-Fi to the PurpleAir database of registered monitors. The PA-II-SD monitor combines Wi-Fi transmission with onboard storage on a microSD card mounted inside the monitor housing; this monitor costs slightly more than the PA-II. We used a combination of PA-II and PA-II-SD monitors in this study.

For both monitor models, Application Programming Interface (API) calling can be used to download data from the PurpleAir database if the device is connected and transmitting data via Wi-Fi. The API keys can be created using PurpleAir Develop developer dashboard. The sensor index and read key of the registered monitor are needed for API calling, which can be found in the URL of the monitor on the PurpleAir map (for public monitors, i.e., those marked as public during setup, only a sensor index is needed to view and download data). After locating the monitor on the PurpleAir map and clicking its marker, the sensor index is the 5 digits appearing after ‘select = ’ in the URL, and the read key (for private monitors) is the 16 digits after ‘key = ’ in the URL. We wrote a custom Python script to conduct PurpleAir API calling and data appending for this study; the script is available for download (https://github.com/berg-lab/PurpleAir). In March 2023, with the release of the PurpleAir Develop dashboard (https://develop.purpleair.com/home), PurpleAir API usage began consuming points to download data. Each new user account is provided initially with 1 million free points, after which users must pay for additional points to download data. However, PurpleAir has noted that users can download data from the monitors that they own for free by contacting PurpleAir and providing their monitor information and API key. Details for the costs of PurpleAir Wi-Fi data extraction via API calling are included in Sect. 2 of the SI (and Tables S1–S3).

For the PA-II-SD monitors only, a built-in microSD card holder allows for on-board storage on a microSD card. The monitors typically come with a 16 or 32 GB formatted microSD card, which allows for effectively unlimited storage given the small file sizes of text-based readings that are generated and stored. The microSD card serves as a backup to Wi-Fi; the monitor does not read from the microSD card and push data to the PurpleAir database via Wi-Fi transmission (PurpleAir 2023b). Moreover, a battery powered real time clock (RTC) inside the device records time stamps in Coordinated Universal Time (UTC) time (not local time), and, if a PA-II-SD monitor is deployed without first syncing to Wi-Fi, the file timestamps will be recorded with dates of either 1970 or 2000 (note that the timestamps in the data files stored on the microSD card will be in the correct year, albeit in UTC). The data stored on microSD are recorded at 2-min intervals without the ability to adjust, and the first data recordings appear shortly after the time at which the unit is powered on. A new “.csv” file is recorded on the microSD card for every day that data is recorded. The PurpleAir Data Download Tool also now enables downloading the data using the desired time zone, which also accounts for daylight savings time, but these features were not in place at the time of our measurements described herein. We explore these resulting data structures further in the results section of this manuscript.

### Field data collection

The FRESSCA project aims to evaluate interventions for reducing the infiltration of wildfire smoke from outdoors, including portable high efficiency particulate air (HEPA) indoor air cleaners, low-cost box fan and filter combinations, and a customized evaporative cooler (EC) filtration solution (Singh et al. 2025). The Central California Environmental Justice Network (CCEJN) recruited study participants in three counties in California’s San Joaquin Valley (Fresno, Kings, and Kern Counties). Participants were recruited for their use of ECs in their homes as an affordable alternative to air conditioners; ECs introduce large quantities of unfiltered outdoor air. One PurpleAir PA-II monitor was deployed indoors in each recruited home to monitor PM concentrations throughout the summer wildfire season. PA-II monitors were also installed outdoors at several locations near clusters of homes, usually outside a recruited home of convenience that was also located near other recruited homes.

In the first pilot year of field testing (2022), participants in 31 homes were recruited in three communities within Fresno and Kern Counties (Coalinga, Arvin, and Lamont, CA); 21 successfully completed field data collection. In April and May of 2022, we obtained written informed consent from study participants and permission from landlords (where applicable). During that time, we pilot-tested project questionnaires, installed PurpleAir PA-II monitors in each home, and installed five PA-II monitors in nearby outdoor locations. In July 2022, we deployed various filtration interventions for pilot testing in the study homes. The PA-II monitors were then retrieved in October 2022. In the second year of field testing (2023), participants in 58 homes were recruited in Fresno, Kings, and Kern Counties; 49 homes completed field data collection. In this expanded intervention year, some homes received PA-II monitors prior to intervention installation (i.e., as early as April 2023); a combination of portable air cleaners (PACs) with HEPA filters and, for approximately half the homes, a custom EC filtration solution was installed beginning in July 2023; and recruitment and monitoring continued until October 2023, at which point all equipment was retrieved from the study homes. Throughout the study periods, team members monitored the PA-IIs connected to Wi-Fi to ensure they were online and collecting/transmitting data. If monitors were offline for > 7 days, they were reported to the CCEJN field team to troubleshoot further. Here we describe the field-collected data in aggregate without specificity to the interventions (which will come in a forthcoming manuscript) to provide practical insights and lessons learned through our experiences using these monitors.

### Indoor Co-location Measurements

To investigate the performance of the PA-II monitors used in the field study relative to each other, indoor co-location measurements were conducted at multiple time points in 2023 and 2024. Since the FRESSCA study objectives were built primarily on relative sensor comparisons (e.g., seeking indoor/outdoor concentration ratios between homes and within homes before and after interventions), the decision was made to focus on sensor performance relative to each other, rather than co-location with a reference monitor (which, as mentioned in Sect. 1, has already been conducted in many prior studies and has typically demonstrated good performance). Such relative comparisons against each other allow for (1) identifying problematic monitors that perform poorly relative to the group and thus may need to be corrected in subsequent data analysis, excluded from analysis, or withdrawn from the field deployments, and (2) calculation of any co-location factors needed to “calibrate” monitors relative to each other.

**Fig. 2:**
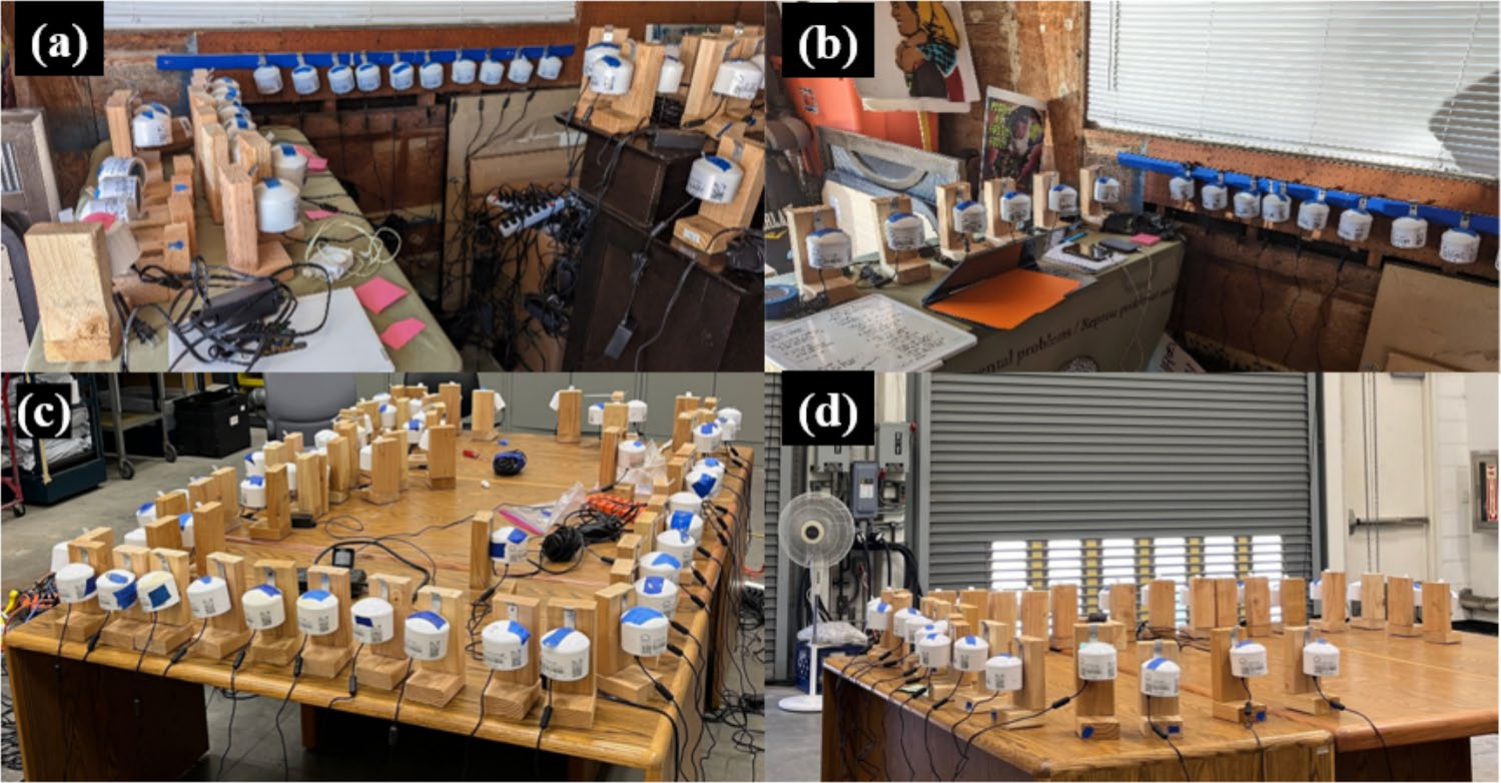
Co-located PA-II monitors in a home garage setting (**a** and **b**) and a large research laboratory garage setting (**c** and **d**)

We conducted side-by-side co-location testing of the PurpleAir PA-II and PA-II-SD monitors used in the field study in several batches of convenience, first in smaller batches at a time at the California Department of Public Health’s (CDPH’s) Richmond campus and in the home garage of one of our community partners after the pilot year deployment (Fig. 2a, b), and then again in larger batches in a large research garage at CDPH after the intervention year deployment (Fig. 2c, d). The locations were chosen because of their proximity to members of the field research team, their adequate size and shelter, and access to AC power and Wi-Fi, which supports the co-location of monitors and data collection. In the initial home garage co-locations, the garage door was kept closed throughout the co-location measurements, with co-location testing occurring over multiple 2–4-week periods from March to June of 2023. Groups of monitors were placed next to each other on two tables by a wall, but were also screwed into a wooden strip along the wall, as not all the monitors fit on the two tables. In the latter CDPH co-locations, the large research garage that was utilized is located approximately 30 m (~ 100 ft) away from an interstate highway, has two large garage doors and was not designed to be well-sealed. Activities inside the space were limited to periodic checking of the monitors by the team in an attempt to minimize indoor sources; however, occupancy was not limited to zero, and thus there was potential for transient indoor sources in addition to infiltration from outdoors. The garage doors were mostly kept closed throughout the co-location measurements, and a portable fan was operated in the background to enhance air mixing. Testing occurred over multiple 2–3-week periods from November 2023 to March 2024. Both co-location strategies were intended to expose the PA-II monitors to indoor PM inside the garage that primarily infiltrated from outdoors, thus minimizing the influence of intermittent indoor sources and their often-unique low-cost particle sensor responses (Park et al. 2023; Zou et al. 2020b).

Table S4 summarizes the batches of PA-II monitor co-location measurements, including their rationale, a summary of monitor types (i.e., Wi-Fi only or Wi-Fi + SD), the method for accessing data, and other relevant details. Because there were limits to the maximum number of Wi-Fi connections that the wireless access point would support (a limit that appears to vary by router make and model), three different batches of monitors were first tested from March to May 2023. Following field deployment in summer 2023, most PA-II monitors were then re-tested in one large batch (n = 57 monitors) in November 2023 to (i) test more monitors against each other in the same group rather than in multiple smaller groups and (ii) investigate whether relative monitor performance had changed over time. Two monitors were not available (i.e., not retrieved from field sites yet) at the time of co-location, thus co-location was repeated with those two monitors at two different subsequent times following the large batch testing in which a singular untested monitor was co-located alongside five of the best performing monitors identified in the November 2023 large batch testing.

### Data management and statistical analysis

All data were processed in Jupyter Notebook version 7.2.2 using custom written code, utilizing Scipy package version 1.13.1 for statistical analyses. Time series data were timestamped, merged, and analyzed using a combination of distributional and comparative statistical tests, including Shapiro–Wilk normality tests (*shapiro*) for distribution fits, Kolmogorov–Smirnov (KS) tests (*ks_2samp*) for large sample comparisons between groups, and calculating Cohen’s *d* for effect size estimates (Cohen 2013). Linear regressions (*linregress*) were conducted to assess performance between monitors, which has been shown to be reasonable for applications in which limited effort in parameter tuning is needed (Liang and Daniels 2022).

## Results and discussion

### Harmonizing data obtained via Wi-Fi (API calling) and microSD cards

Monitor type (i.e., PA-II or PA-II-SD) affected data extraction and time-stamp alignment processes. In general, when the monitor type is either Wi-Fi only or Wi-Fi + SD and the monitor is connected to Wi-Fi, it is preferable to download data by Wi-Fi API calling because it automatically synchronizes the time interval at consistent 10-min intervals for all monitors. However, if a specific monitor could not be found on the PurpleAir map from the link that the field team provided (using the specific sensor index and read key for that sensor to make the API call), or if a monitor could not successfully connect and transmit via Wi-Fi, then the data extracted from the microSD card was used as an alternative data source. Details for extracting Wi-Fi and SD card data are provided below.

**Fig. 3:**
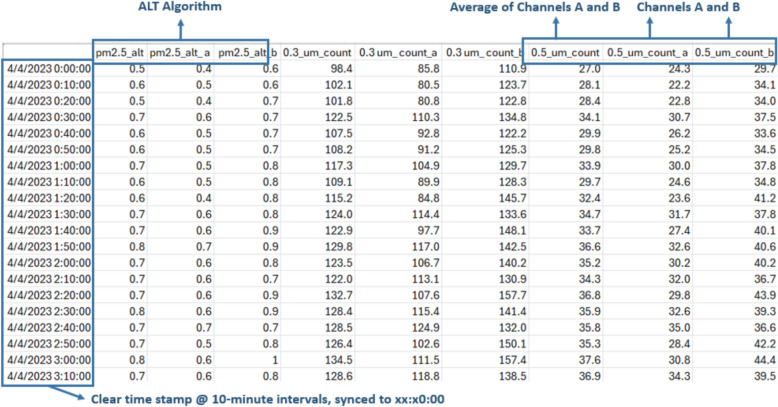
An example of data extracted via the Wi-Fi API

Procedures for downloading and cleaning data, as well as the resulting data structures, are different between Wi-Fi API calling and manually extracting from the microSD cards. The data extracted via Wi-Fi API are structured differently, as follows (Fig. 3):

- The default time interval of API-called data is every 10 min.
- In 10-min intervals, the time structure is clear and consistent, in hh:mm:ss intervals of hh:mm:00, synchronized for all monitors such that each recording from multiple devices has the same 10-min interval timestamp.
- For each PM bin (e.g., PM_2.5_, PM_10_, 0.3_µm_count, etc.), three values are provided: *pmXX_a* (result from monitor channel A), *pmXX_b* (result from monitor channel B), and *pmXX* (the average of *pmXX_a* and *pmXX_b*).
- Other PM metrics such as ‘*pm2.5_alt*’, which integrate algorithms developed by researchers, can also be downloaded directly.

**Fig. 4:**
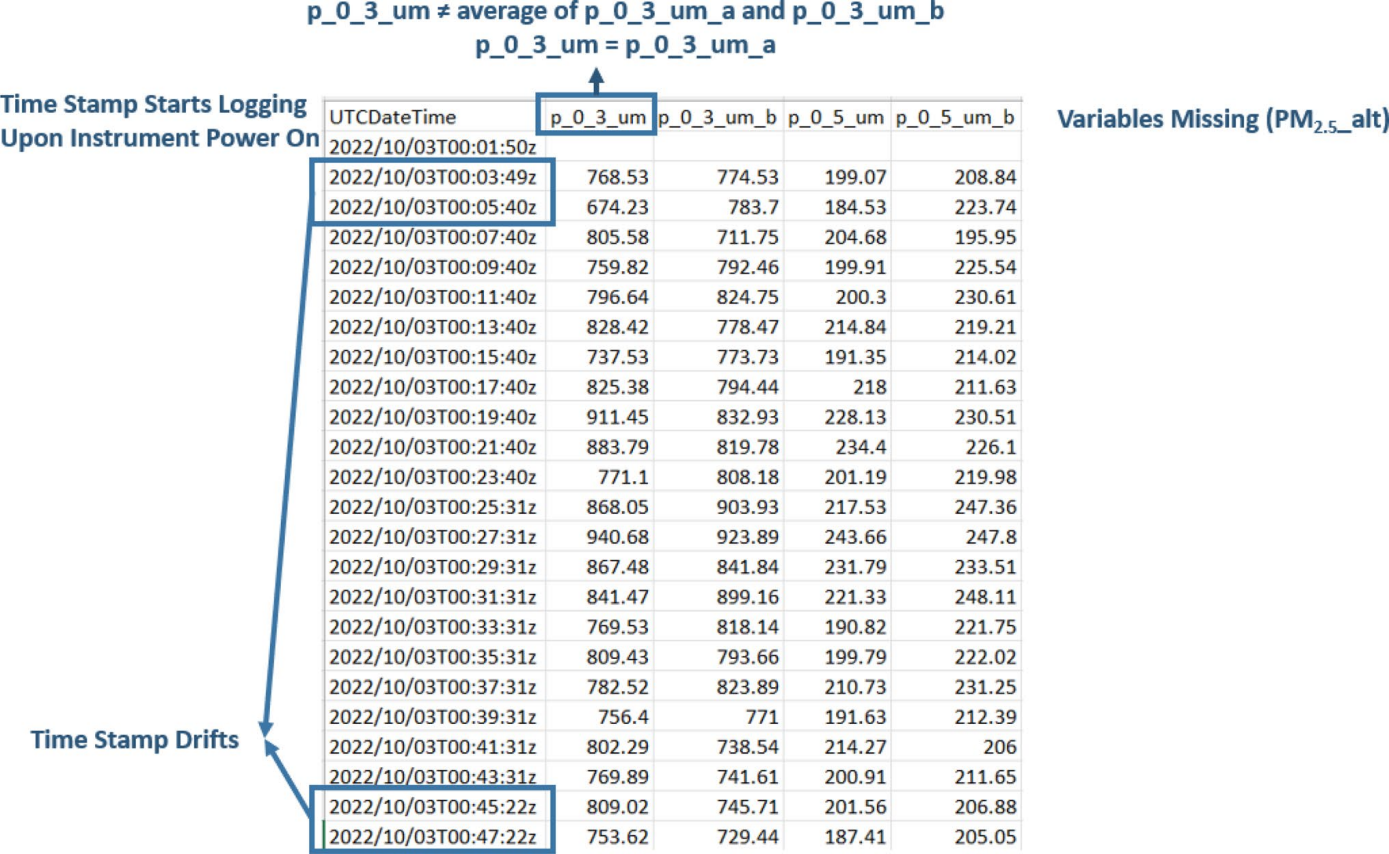
An example of data extracted from microSD card

The data extracted from microSD card readings are structured as follows (Fig. 4):

- Each data point is stored at approximately 2-min intervals, with the first timestamp beginning when the monitor was plugged in and powered on (Couzo et al. 2024). A new data file is recorded at the end of every 24-h day (at 11:59 pm UTC).
- For each PM metric, there are two values recorded on the SD card: *pmYY_b* and *pmYY*. For monitors with both SD and Wi-Fi capabilities, it was observed that value *pmYY_b* from the SD card was found to be the same data as that from the channel B values *pmXX_b* in Wi-Fi transmitted data, but the *pmYY* value from the SD card is not the average of channel A and B like the Wi-Fi transmitted data *pmXX*, but rather is equal to the channel A values *pmXX_a* from Wi-Fi records. Therefore, in order to directly compare data collected from the SD cards and Wi-Fi transmission, the *pmYY* values from the SD card data should be relabeled as *pmYY_a* and a new variable *pmYY* should be calculated as the average of the new *pmYY_a* and the original *pmYY_b* to generate an average of the two channel values in a manner that is equivalent to the Wi-Fi transmitted data.
- There is no parameter ‘*pm2.5_alt*’ from the SD card, at least in the batches of monitors that we tested; instead, it must be calculated in post-processing.

Moreover, the time-stamped data records from the microSD card readings drifted with some regularity. For example, some records “lost” 10 s on a repeatable pattern of every 20 min, such that instead of recording 720 data points every 24 h (i.e., 30 records per hour × 24 h), they may record 725 or 726 data points. Some records “lost” varying amounts of time (usually 5–7 s) every 20 min. In these cases, still 723 to 725 data points are stored per 24-h day. At other times, time stamped data records in the SD card readings drifted irregularly, for example, “losing” 1 s at seemingly random intervals. In these cases, still 719 or 720 data points are stored per 24-h day, suggesting that the device may both “lose” time and “catch up” time within the day of recorded data. Such behaviors were largely unpredictable; for example, at times, a monitor that was “losing” data one day could return to regularly expected recordings the following day without intervention. While we do not know the exact source of these timing issues, it could be due to internal clock precision, differences in firmware, buffered SD card write operations, or timing inconsistencies in the devices’ data logging logic.

The highest time-resolution available for Wi-Fi API called data is also at 2-min intervals, like the SD card readings; also, these 2-min intervals are not recorded at hh:mm:00 like the 10-min interval data are. Upon further inspection, the 2-min values in each data field are the same values for both Wi-Fi data and SD card data; however, the timestamps of Wi-Fi data are usually 1-s slower than the SD card readings. This might be because the Wi-Fi data records the timestamp (using internet-based clock time) when the cloud storage receives the data.

Because our field studies and laboratory co-location measurements both utilized a mix of SD and Wi-Fi data collection and we desire concurrent (time-synchronized) measurements to compare indoor and outdoor concentrations measured simultaneously across monitors and/or homes, these inconsistencies in timekeeping between the SD and Wi-Fi collected data necessitate further post-processing to synchronize data between monitors for subsequent analysis. Even among only the SD card collected data from different monitors measuring concurrently, the raw 2-min data are not synchronized in time across monitors because data recording begins shortly after the device is powered on and because of the drifts and timestamp differences explained above. For example, three different monitors measuring simultaneously might record data to SD cards at 12:00:12, 12:00:28, and 12:00:47, each most closely adjacent to a 12:00 or 12:01 time stamp, but none explicitly recorded at 12:00 or 12:01. If we assume that the RTCs are all accurate for each device (i.e., they start with the same time stamp and do not drift significantly over the duration of deployment, an assumption that has not been tested but may not be valid (Ali 2015)), an average of five consecutive 2-min intervals can be used to generate 10-min interval time stamped data that are equivalent to 10-min interval data that would be extracted via Wi-Fi (if available). This allows for effective synchronization but requires some logic to be applied in post-processing. The following modifications to data extracted from SD cards are made to harmonize with data collected via monitors concurrently transmitting via Wi-Fi:

1. Rename value *pmYY* as *pmYY_a*.
2. Resample the 2-min interval data to 10-min intervals by averaging the data from hh:m0:00 to hh:m9:59 to align with the 10-min interval data at hh:m0:00.
3. Calculate the average of the 10-min-averaged new variable *pmYY_a* and the 10-min-averaged original *pmYY_b*, and rename it as *pmYY*.
4. Calculate *pm2.5_alt_a*, *pm2.5_alt_b*, and *pm2.5_alt* using Eq. 1 (L. Wallace et al. 2022a, b) or another algorithm as needed:
  1 $${PM}_{2.5\_ALT}=CF\times (0.00030418\times \text{N}1+ 0.0018512\times \text{N}2+0.02069706\times \text{N}3)$$

where N1 = [0.3_µm_count]—[0.5_µm_count], N2 = [0.5_µm_count]—[1.0_µm_count], and N3 = [1.0_µm_count]—[2.5_µm_count], all in units of particles per deciliter (#/dL). N1, N2 and N3 represent three size categories, where the particles in each category are considered to have the same diameter as the geometric mean of the boundaries of the category. Particles are assumed to be spherical. Therefore, the coefficient before each size category is the mass of a single particle in that size category, calculated by multiplying the volume of each particle $(\pi \times \frac{{diameter}^{3}}{6})$ by the density (assumed 1 g/m^3^). CF is a calibration factor, originally assumed to be 3 in the ALT-CF3 algorithm (Wallace et al. 2021) and later adjusted to 3.4 to improve accuracy (L. A. Wallace et al. 2022a, b; Wallace 2023). Here we utilize CF = 3 because at the time of data downloading in 2023, a CF of 3.4 was not yet published and available in the data download environment.

**Fig. 5:**
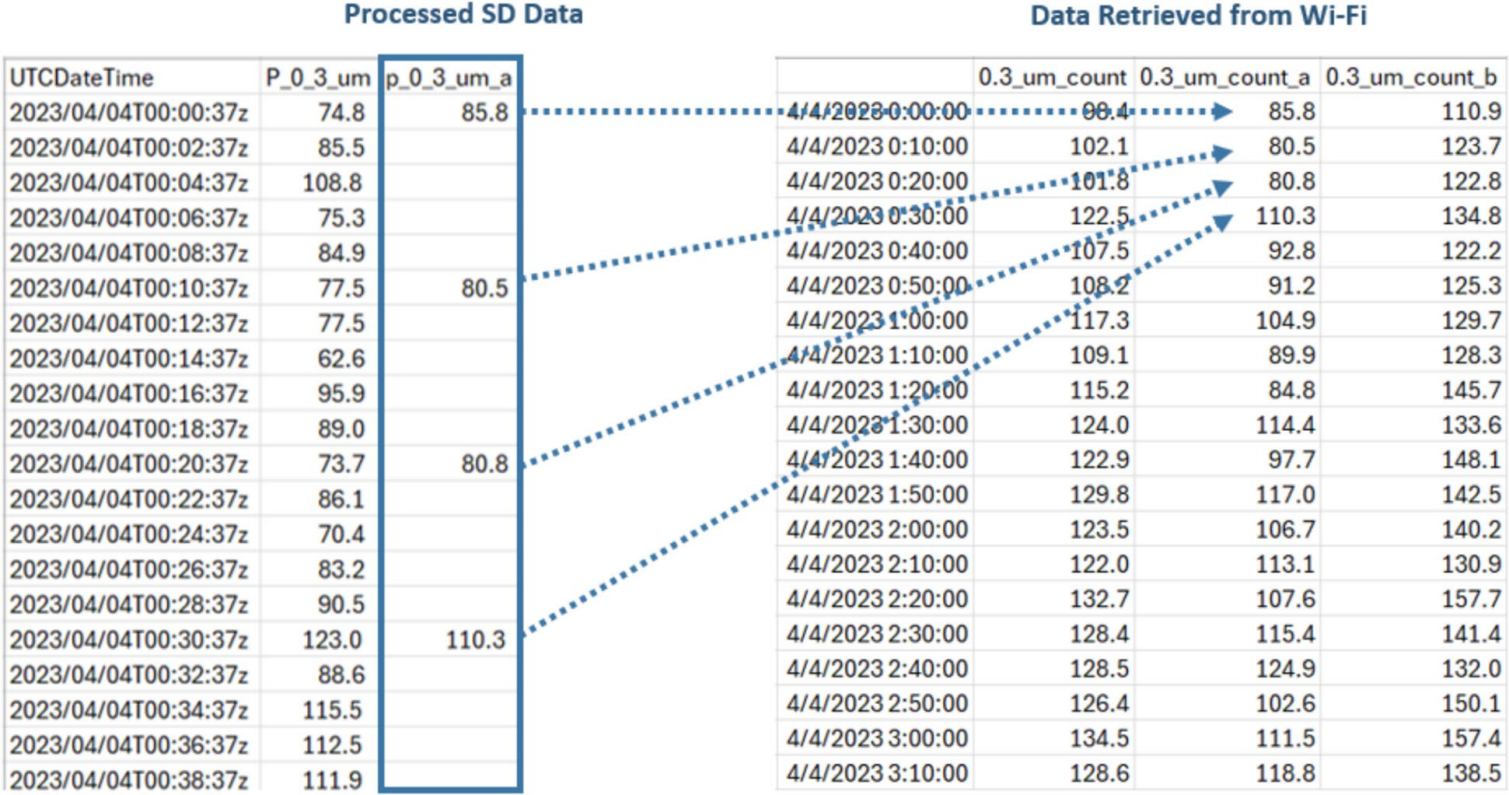
Comparison of post-processed SD data and Wi-Fi extracted data

Equation 1 is applied separately for channels A and B to derive *PM*_*2.5_*_*alt_a* and *PM*_*2.5_*_*alt_b*, which are then averaged to obtain *PM*_*2.5_*_*alt*. These procedures led to harmonized data from both SD and Wi-Fi collection at consistent, concurrent 10-min time intervals. An example of these procedures is shown in Fig. 5 for illustrative purposes.

### Indoor Co-location Results

During the co-location tests after the pilot year deployment (in Spring 2023), 34 of the first 35 monitors that were tested from March 3, 2023 to April 3, 2023, were successfully registered, but only 28 had data successfully transmitted or extracted for calibration; thus, six monitors yielded no data (all were PA-II Wi-Fi only monitor, perhaps due to weak or failed Wi-Fi connection or losing power, although we do not know exactly why). There was no data loss from the next two calibration periods (total of 43 monitors). During the co-location test after the intervention year deployment (late 2023 and early 2024), no data were missing from the first 57 tested monitors in the November 13, 2023 to November 29, 2023 test batch. Five of the highest performing monitors from that group (i.e., regression slope and R^2^ were both close to 1) were then used to co-locate alongside two additional monitors, phased just one at a time, that were retrieved from the field at a later date due to logistical constraints in scheduling equipment retrieval from two homes. During these co-location tests, no data loss occurred. Thus, given the strengths of the post-intervention co-location measurements (i.e., mostly all monitors were tested in one batch, with a high success of data transfer/extraction), we focus primarily on these co-location results while emphasizing lessons learned throughout the process.

For each of the data sets resulting from the indoor co-location measurements, we used a combination of linear regression on concurrent data from each monitor against the other monitors, with two goals: (1) identifying any problematic monitors that performed poorly relative to the group and thus may need to be withdrawn from the field deployments or field data analysis and (2) calculating any co-location factors needed to “calibrate” monitors relative to each other to yield reasonably equivalent results and allow for direct comparison between concurrently deployed monitors (i.e., to other indoor monitors and to nearby outdoor monitors). For goal #1, we chose the monitor that recorded the greatest number of data points in the co-location measurements as an arbitrary reference monitor to which all others could be compared to, which allows for identifying anomalous behavior. For goal #2, we first excluded data from any poorly performing monitors identified in goal #1 and then used the average values of all monitors in each co-location group at each concurrent time interval (whether truly concurrent or synchronized in post-processing) as the reference. The use of mean values from a group of concurrently tested monitors eliminates the need to utilize a singular arbitrary reference (Kang et al. 2025).

Here, we focus on results from the co-location testing conducted following the second year of the intervention because it includes the largest number of concurrently deployed monitors. Given our focus on PM_2.5_, we focus primarily on the *pm2.5_alt* data value, as among all PM_2.5_ algorithms, *pm2.5_alt* is the only algorithm that is independent from the Plantower algorithms, with better accuracy and a lower limit of detection (LOD) compared to the Plantower algorithms or the EPA algorithms (Wallace et al. 2022a, b; Barkjohn et al. 2021). For the co-location analysis, we defined the following data inclusion criteria for each monitor:

1. Must include at least 288 data points of co-located concurrent data (i.e., 48 h of concurrent 10-min interval data in total, not necessarily consecutive in time).
2. The maximum concentration in *pm2.5_alt* of the reference (either arbitrary or mean) must be greater than 1 µg/m^3^ to ensure concentrations are in the functional range of the monitor.
3. The maximum concentration in *pm2.5_alt* of the reference(s) must be greater than twice the minimum *pm2.5_alt* concentration of the reference to ensure sufficient range is covered for linear regression.
4. The absolute range of *pm2.5_alt* concentrations of the reference(s) must be greater than twice the minimum *pm2.5_alt* concentration of the reference(s) to ensure sufficient range for linear regression.

In our co-location testing, these criteria were met by our 10-min interval reference data having a minimum and maximum *pm2.5_alt* of 0.8 µg/m^3^ and 22.7 µg/m^3^, respectively.

We first used the collected, time-synchronized 10-min interval data from co-location measurements to compare each monitor with an arbitrary reference, conducting linear regression for 16 output data values: [0.3_um_count], [0.5_um_count], [1.0_um_count], [2.5_um_count], [5.0_um_count], [10.0_um_count], [pm2.5_alt], [pm1.0_cf_1], [pm2.5_cf_1], [pm10.0_cf_1], [pm1.0_atm], [pm2.5_atm], [pm10.0_atm], [temperature], [humidity] and [pressure]. In each regression, the dependent variable (y-value) is the data from the arbitrary reference monitor, and the independent variable (x-value) is the data from each other monitor, repeated for each monitor. A zero intercept was assumed for all parameters except temperature, relative humidity, and pressure, which showed the need for offsets.

**Fig. 6:**
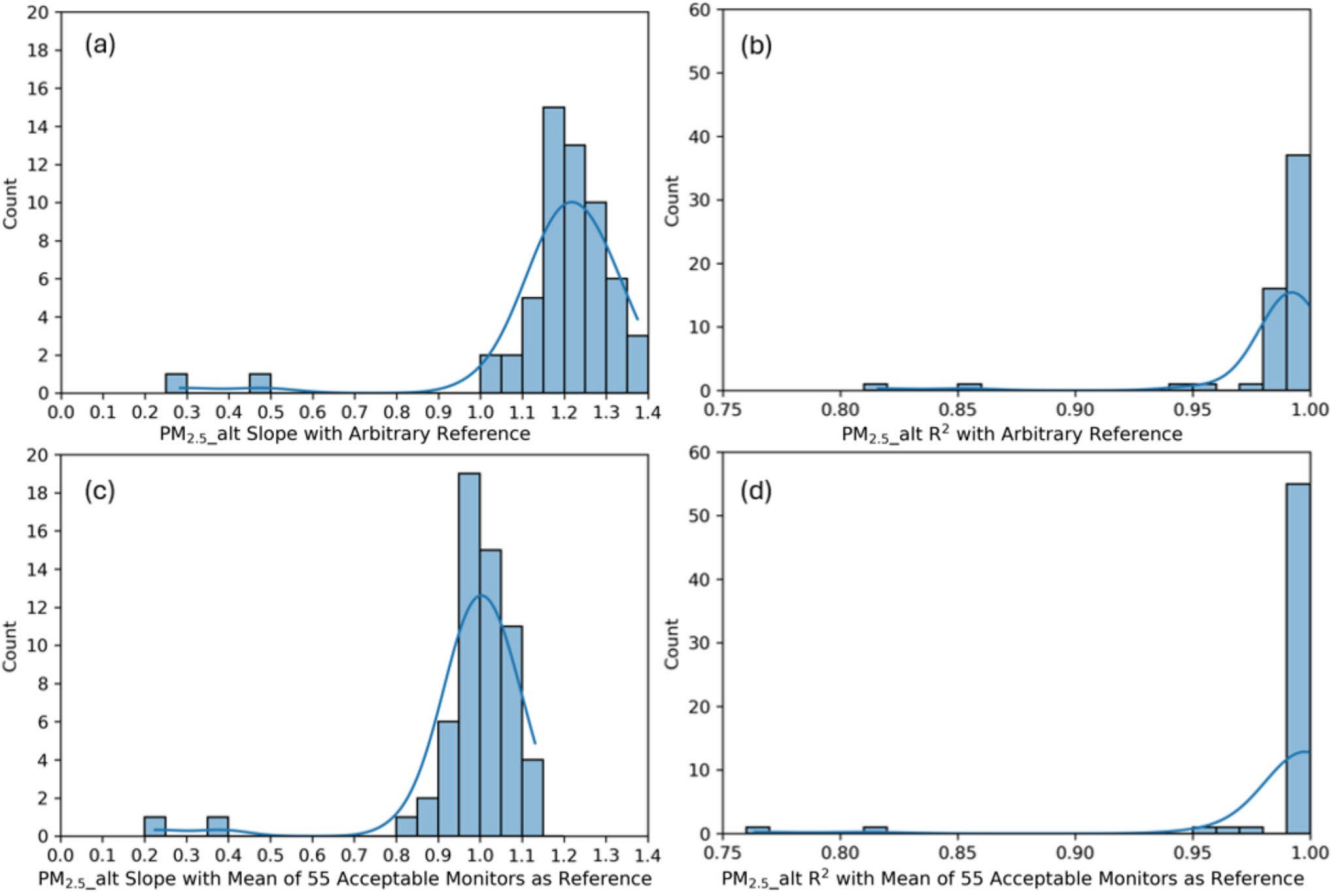
Histograms of slope and R^2^ of *pm2.5_alt* from each monitor with an arbitrary reference monitor (**a** and **b**) and the mean of monitors as a reference (c and d) for co-locations conducted in the intervention year

Figure 6 compares regression statistics (slope and R^2^) for *pm2.5_alt* concentrations from all monitors during the co-location measurements using (i) an arbitrary reference monitor and (ii) the mean-of-monitors as reference. Figure S2 and Figure S3 in the SI show individual scatter plots of *pm2.5_alt* concentrations from each monitor compared to (i) the arbitrary reference monitor and (ii) the mean of 55 acceptable monitors as reference, respectively, from the largest batch of co-location testing (n = 57 monitors), along with regression statistics (i.e., slope, R^2^, and N, the number of 10-min interval data points used in each regression). Figure S4 in the SI also shows a time-series profile of the average PM_2.5__alt concentrations from 55 acceptable monitors during the primary garage co-location measurements, which suggests that the garage appeared to include a mix of ambient-infiltrated PM and intermittent, yet uncharacterized, indoor PM sources. It is noteworthy that while the LOD for PurpleAir PA-II monitors with the ALT-CF3 algorithm has been reported to be less than 1 µg/m^3^ (Wallace 2022), according to Figure S2 and Figure S3, PM_2.5__alt concentrations remain highly correlated between each monitor even below 1 µg/m^3^.

With the arbitrary reference, regression slopes most frequently occurred between 1.15 and 1.20 (n = 15), with only 7% of monitors (n = 4) showing slopes between 0.9 and 1.1; however, 65% of monitors (n = 37) achieved R^2^ values above 0.99 (Fig. 6a, b). In fact, each monitor-to-reference comparison had *pm2.5_alt* R^2^ greater than or equal to 0.8, which is a commonly used threshold for minimum acceptable coefficient of determination in other studies on low-cost particle sensor performance (Zou et al. 2020a). All but two monitor comparisons had R^2^ greater than 0.95 (i.e., 55 out of 57 comparisons, or 96%, yielded R^2^ > 0.9). The slopes of regression lines varied between 0.85 and 1.15 for most monitor comparisons as well. Upon visual inspection, the two monitors that had the lowest R^2^ with arbitrary reference values also had slopes well outside the range of the others (i.e., 0.48 and 0.28). The performance of these monitors warranted further investigation to determine if they should be pulled from field use (and data excluded entirely) or if they could be corrected in post-processing via a different form of co-location factors. Ultimately, the two monitors were identified as problematic and were excluded from the remaining analyses.

After excluding data from poorly performing monitors, we then used the average values of all remaining acceptable monitors in each co-location group at each concurrent time interval as a collective group reference. Using the mean values from all monitors as the reference, the slope distribution shifted and concentrated more tightly around 1 (Fig. 6c, d). The highest frequency of slopes occurred between 0.95 and 1.00 (n = 19), with 58% of monitors (n = 33) showing slopes between 0.9 and 1.1. Similarly, the R^2^ distribution showed stronger convergence toward unity, with 91% of monitors achieving R^2^ values above 0.99. These results suggest highly repeatable measurements at high time resolution of 10-min intervals among the vast majority of concurrently deployed PA-II and PA-II-SD monitors.

Figure S5 shows an example of PM_2.5__alt concentrations resulting from one of the poorer performing monitors against the arbitrary reference, including the average of A and B channels and the channels A and B separately to investigate whether one of the channels was problematic. Figure S5a indicates that the regression between a monitor’s PM_2.5__alt average channel and the reference has an offset (intercept), while Figs. S5b and S5c also show a similar offset for the individual channel responses. Since both channel A and B show similar regression results, with R^2^ similar to the mean of ~ 0.8, an intercept could be added to the regression to improve performance and maintain usability (Fig. S6).

Tables 1 and 2 summarize regression statistics (e.g., mean, minimum, and maximum R^2^ and slope) of several other variables from PA-II and PA-II-SD monitors again using (i) an arbitrary reference monitor and (ii) the mean-of-monitors as reference, respectively. Additional regression details are provided in Table S5. Regression statistics were stronger for all variables with the mean-of-monitors as the reference compared to the arbitrary reference; therefore, the majority of the results discussion focuses on these regression results.

**Table 1:** Summary of R^2^ and slopes from linear regressions (all with zero intercept except temperature, relative humidity, and pressure) of 56 monitors versus a single arbitrary reference monitor in indoor co-location measurements

	R^2^ between monitors			Slope between monitors		
	Mean	Min	Max	Mean	Min	Max
pm2.5_alt	0.98	0.82	1.00	1.19	0.28	1.37
0.3_um_count	0.94	0.76	0.99	1.56	0.16	1.89
0.5_um_count	0.98	0.81	1.00	1.23	0.22	1.46
1.0_um_count	0.99	0.99	1.00	1.02	0.89	1.20
2.5_um_count	0.96	0.92	0.98	0.76	0.50	1.38
5.0_um_count	0.85	0.76	0.92	0.89	0.27	1.63
10.0_um_count	0.70	0.58	0.86	1.15	0.20	3.59
pm1.0_cf_1	0.97	0.75	1.00	1.25	0.23	1.52
pm2.5_cf_1	0.98	0.77	1.00	1.14	0.30	1.36
pm10.0_cf_1	0.99	0.77	1.00	1.11	0.31	1.31
pm1.0_atm	0.97	0.71	1.00	1.24	0.24	1.50
pm2.5_atm	0.98	0.75	1.00	1.14	0.30	1.34
pm10.0_atm	0.99	0.76	1.00	1.11	0.31	1.31
temperature	0.94	0.00	0.98	1.08	0.95	1.23
humidity	1.00	0.97	1.00	1.13	0.74	1.42
pressure	0.99	0.51	1.00	0.99	0.76	1.00

**Table 2:** Summary of R^2^ and slopes from linear regressions (all with zero intercept except temperature, relative humidity, and pressure) of 55 ‘acceptable’ monitors versus the mean of all 55 monitors as the reference in indoor co-location measurements conducted in the intervention year

	R^2^ between monitors			Slope between monitors		
	Mean	Min	Max	Mean	Min	Max
pm2.5_alt	1.00	0.96	1.00	1.00	0.82	1.13
0.3_um_count	0.98	0.79	1.00	1.00	0.60	1.19
0.5_um_count	0.99	0.90	1.00	1.00	0.78	1.16
1.0_um_count	1.00	1.00	1.00	1.00	0.87	1.17
2.5_um_count	0.99	0.97	0.99	1.02	0.68	1.86
5.0_um_count	0.94	0.88	0.97	1.07	0.46	1.93
10.0_um_count	0.90	0.78	0.95	1.15	0.27	3.52
pm1.0_cf_1	0.99	0.91	1.00	1.00	0.78	1.20
pm2.5_cf_1	1.00	0.95	1.00	1.00	0.85	1.17
pm10.0_cf_1	1.00	0.96	1.00	1.00	0.85	1.16
pm1.0_atm	0.99	0.92	1.00	1.00	0.78	1.19
pm2.5_atm	1.00	0.95	1.00	1.00	0.86	1.16
pm10.0_atm	1.00	0.96	1.00	1.00	0.86	1.15
temperature	0.97	0.00	0.99	0.96	0.83	1.11
humidity	1.00	0.98	1.00	1.00	0.66	1.27
pressure	0.99	0.53	1.00	0.98	0.78	0.99

In both Tables 1 and 2, particle size-resolved measurements indicate increasing variability in performance with increasing particle size, which is consistent with recent literature demonstrating poorer performance for coarse mode particles (Molina Rueda et al. 2023). Co-location measurements for smaller particle sizes (0.3 μm and 0.5 μm bins) showed stronger correlation (most R^2^ > 0.95 and most slopes are between 0.85 and 1.15 for mean-of-monitors regressions) than larger particles (with R^2^ values as low as 0.78 and slopes varying from 0.46 to 3.52 for 10.0 μm bin for mean-of-monitors regressions). The environmental parameters (temperature, humidity, and pressure) showed excellent consistency across all monitors, with most R^2^ values above 0.99 and slopes between 0.85 and 1.15 in the mean-of-monitors regressions.

One monitor had a temperature sensor failure, which was detected by an incalculable slope in Table S5. To detect such extreme anomalies, it is recommended to conduct at least a brief, short-term data collection check in the laboratory setting before field deployment to ensure the sensors, including temperature sensors, operate correctly and log data. Worth noting is that we also did not observe major differences in instrument responses between the pilot year and intervention year co-location results, suggesting there was no apparent drift in monitor responses over time. For example, in three different pilot year co-location tests with smaller numbers of monitors, R^2^ for PM_2.5__alt varied from 0.98 to 1 with a mean of 1 in group 1, from 0.99 to 1 with a mean of 1 in group 2, and from 0.94 to 1 with a mean of 0.98 in group 3 (excluding a total of 3 problematic monitors).

To include data from a monitor in subsequent analysis, we defined a minimum acceptable coefficient of determination (R^2^) for each monitor-to-reference comparison as 0.8 or greater, similar to prior studies on low-cost particle sensors (Zou et al. 2020a). For the monitors with R^2^ of all parameters within acceptable limits, we used their regression slopes to apply a co-location factor to their raw data collected in the field study. As such, each monitor had a specific factor for each parameter. For the monitors with R^2^ below 0.8 for any parameters, we investigated further by visually inspecting the co-location data and conducting linear regressions for the two monitor channels separately ([XX_a] and [XX_b]) to explore whether any poor co-location results stemmed from a single faulty sensor. If the R^2^ of all parameters from one of the two channels was within acceptable limits, we flagged the monitor and either replaced or continued to use the remaining functional channel output in subsequent analysis. If neither of the two channels had all parameters within acceptable limits, we flagged the monitor and excluded its field-collected data, then repeated the co-location regression process, excluding data from the flagged monitors.

Ideally, all monitors can be tested together via co-location measurements, but in our study, two monitors were returned later to the garage, separate from the larger group. To avoid redundant work of setting out all monitors, we selected five well-performing monitors based on the results of the primary co-location test to co-locate with the late-returned devices. The selection criteria for high performing monitors included slope and R^2^ as close to 1 as possible. We then assume that the average of these five monitors could reasonably approximate the average of all monitors in the primary test group, such that co-location of the late-returned monitors with these five devices could be used to reasonably represent all devices in the primary group, leading to similar co-location results as would be obtained from testing all devices together.

Ultimately, with this logic, we identified a total of 5 potentially problematic monitors via co-location testing throughout this study. One monitor showed good performance using only one sensor channel and was thus flagged for inclusion, but only when using data from channel B. Two additional monitors were flagged for inclusion with an offset introduced in data post-processing. The other two were excluded from further use due to poor performance and returned to PurpleAir. As such, the overall acceptability rate among all the monitors we tested was ~ 97% (excluding 2 out of 59) if we include the two flagged monitors identified in co-location, or ~ 92% if we used stricter criteria and excluded those two as well (excluding 5 out of 59). This is a relatively high rate of acceptability for many types of applications, although co-location testing prior to deployments for large field studies of indoor and/or outdoor air is still recommended to identify any problematic monitors prior to data collection, if possible. In this study, we chose to co-locate following field campaigns due primarily to logistical constraints.

### Field Data Capture Rates

During the 2023 field-testing period, a total of 49 homes completed the field data collection phase. Five homes were subsequently excluded from analysis due to incomplete intervention implementation, delayed recruitment timing, or poor PA-II monitor co-location results. The final analytical dataset comprised 44 homes (21 in Kern County and 23 in Fresno County). Of the eight outdoor monitoring locations, two PA-II monitors were excluded from analysis due to late installation or excessive distance from study residences, resulting in six outdoor monitors for the final analysis. The field deployment period extended approximately six months, with initial installations beginning as early as April 2023 and equipment retrieval completed in October 2023. The dataset was trimmed to include only periods of time when the PA was installed inside or outside each home as the functional maximum number of data points for each field-deployed monitor, resulting in a maximum of 893,158 data points at 10-min intervals across all 50 monitors (44 monitors located indoors and six located in nearby outdoor locations).

**Fig. 7:**
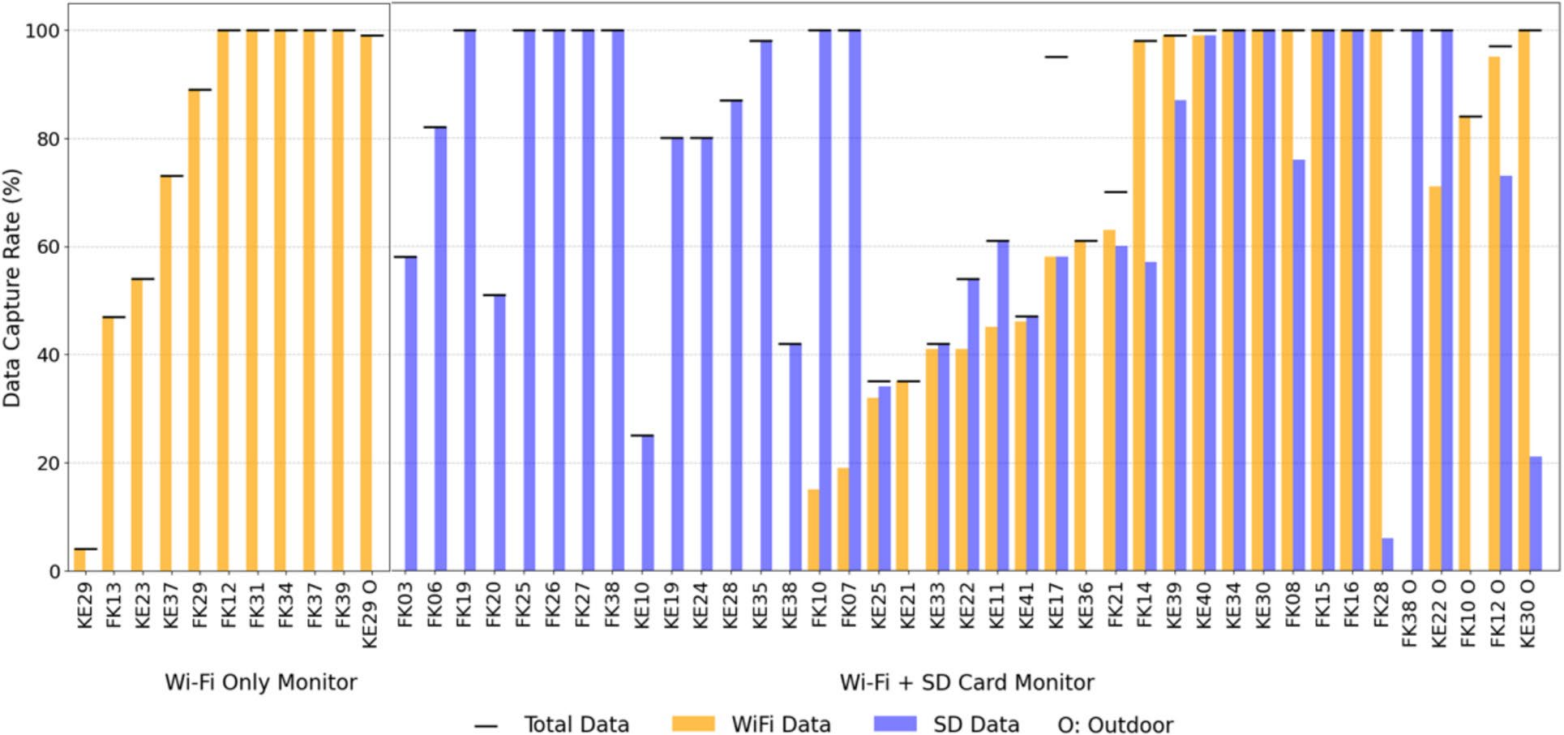
Field data capture rate of PA-II (Wi-Fi only) and PA-II-SD (Wi-Fi + SD) monitors, comparing data captured via Wi-Fi transmission, SD card storage, and the total data captured by the monitors. Plot is sorted by Wi-Fi data capture rate within each monitor type. FK = Fresno/Kings Counties; KE = Kern County

Of the 50 PA-II monitors that successfully recorded data and met inclusion criteria for analysis, 39 were PA-II-SD monitors and 11 were PA-II monitors (without any SD backup). Data were collected from the field deployments using both Wi-Fi API calling and microSD card downloading and merging as backup, as applicable. The research team attempted to connect monitors via Wi-Fi in all home deployments, although four homes did not have access to Wi-Fi and thus 46 monitors (40 indoors and six outdoors) were successfully initially connected to Wi-Fi in the field. However, not all Wi-Fi data transmission was successful in practice. To evaluate the success of data transmission via Wi-Fi for both indoor and outdoor monitors, we first compared the number of 10-min interval data points successfully transmitted via Wi-Fi in the field study to the maximum possible number of 10-min interval data points that we would expect from the start and end dates and times of monitor deployment in each home. We also calculated the number of data points recorded by onboard microSD card storage in the 39 PA-II-SD monitors and then added the total number of time stamps recorded between both onboard microSD and Wi-Fi transmission to calculate the total field data capture rates for each monitor in each home during the field study. The field data capture rates via both methods, both individually and combined as total, are shown in Fig. 7.

The average data capture rate of the 46 monitors that were connected to Wi-Fi in the field was 56% via Wi-Fi transmission and increased to 82% when integrated with data collected from onboard microSD cards. From the 11 PA-II monitors without onboard storage capabilities, the mean Wi-Fi data capture rate was 79%, with 1 out of 11 (9%) monitors having a Wi-Fi data capture rate less than 20%, 3 out of 11 (27%) monitors having a Wi-Fi data capture rate between 20 and 80%; and 7 out of 11 (63%) monitors having a Wi-Fi data capture rate higher than 80%. From the 35 PA-II-SD monitors that were also successfully connected to Wi-Fi, the Wi-Fi data collection rates varied from 0 to 100%, with a mean of only 44%; 13 out of 35 (37%) monitors had a Wi-Fi data capture rate less than 20%, 10 out of 35 (29%) monitors had a Wi-Fi data capture rate between 20 and 80%; and 12 out of 35 (34%) monitors had a Wi-Fi data capture rate higher than 80%. After integrating Wi-Fi data with onboard microSD card data for these 35 monitors, the mean data capture rate increased from 49 to 83%. The number of monitors having a total data capture rate lower than 20% decreased from 13 to 0, and the number of monitors having a total data capture rate higher than 80% increased from 12 to 27.

Accordingly, if we had only collected data via Wi-Fi transmission, we would have missed more than half of the total desired amount of time-series data. By using onboard microSD card storage as a backup, we nearly doubled the amount of data we were able to collect, as Wi-Fi transmission failures were apparently common in these field settings. We were surprised that the inclusion of onboard microSD card data did not increase the total data capture rate closer to 100%. Investigating further, from the 39 PA-II-SD monitors, the mean SD data capture rate was 69%, with 4 out of 39 (10%) monitors having a SD data capture rate less than 20%, 15 out of 39 (38%) monitors having a SD data capture rate between 20 and 80%; and 20 out of 39 (51%) monitors having a SD data capture rate higher than 80%. No specific pattern of SD data loss was found, as some SD data streams stopped permanently without resuming, while others showed intermittent gaps. As such, some SD data loss was likely attributable to power loss to the device, but some may have been attributable to unknown instrument failures or challenging field conditions (e.g., occupant sensor moves, liquid spills, or insects inhabiting the monitors).

### Laboratory- and Field-Collected Data Summary

**Fig. 8:**
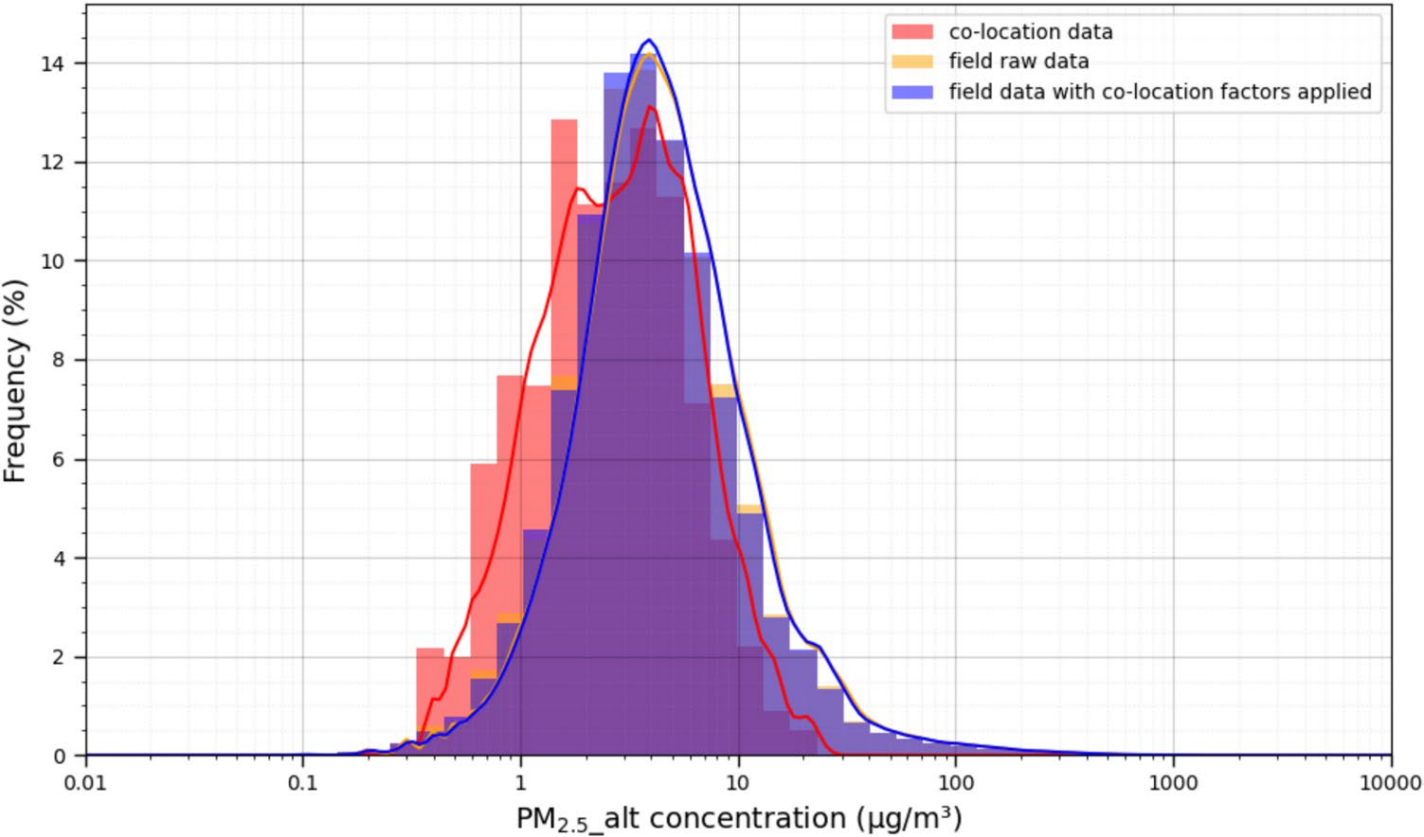
Histograms of PM_2.5__alt measurements from laboratory co-location tests and field tests, both with and without co-location factors applied, combining all indoor and outdoor data together

Figure 8 shows a histogram of PM_2.5__alt data resulting from both the laboratory co-location measurements and the field data collected from all indoor and outdoor monitors in the field study combined (including both raw concentrations and those with co-location factors applied) to compare their data ranges. Values are shown on a log-scale for visual clarity.

The raw PM_2.5__alt concentrations from the indoor co-location tests and the field campaign ranged 0.2–28 µg/m^3^ (mean = 3.9 µg/m^3^; SD = 3.4 µg/m^3^) and 0.05 to 1253 µg/m^3^ (mean = 8.1 µg/m^3^; SD = 21.7 µg/m^3^), respectively. The PM_2.5__alt concentrations from the combination of indoor and outdoor measurements in the field campaign, adjusted with co-location factors applied, range 0.05 to 1209 µg/m^3^ (mean = 8.1 µg/m^3^; SD = 21.8 µg/m^3^). As such, the manufacturer-reported upper limit of detection of 1000 µg/m^3^ was very rarely exceeded in the field data. A detailed summary of the data ranges is also shown in Table S6. Accordingly, the co-location factors are applicable to ~ 97% of the raw field-collected data (i.e., that were below 28 µg/m^3^ raw), but the relative performance among monitors is unknown for the remaining top 3% of data values that were not present in indoor co-location tests. Therefore, when conducting co-location measurements, it may be useful to rely not only on the infiltration of ambient particles but also consider periodically elevating concentrations, ideally using indoor sources that would be reasonably expected to be found in the field deployment locations. Inspection of the distributions of PM_2.5__alt concentrations from the raw field-collected data versus those with co-location factors applied suggests no meaningful differences in the distribution of resulting concentrations (Kolmogorov–Smirnov test statistic ~ 0.007; p < 0.05). This is reasonably expected since the average of the regression slopes from co-location testing for PM_2.5__alt is ~ 1, ranging from 0.82 to 1.13 (Table 2). These results suggest that the application of co-location factors led to small corrections, on average, and thus, co-location testing may not be needed in all community monitoring applications, depending on thresholds for acceptability.

**Fig. 9:**
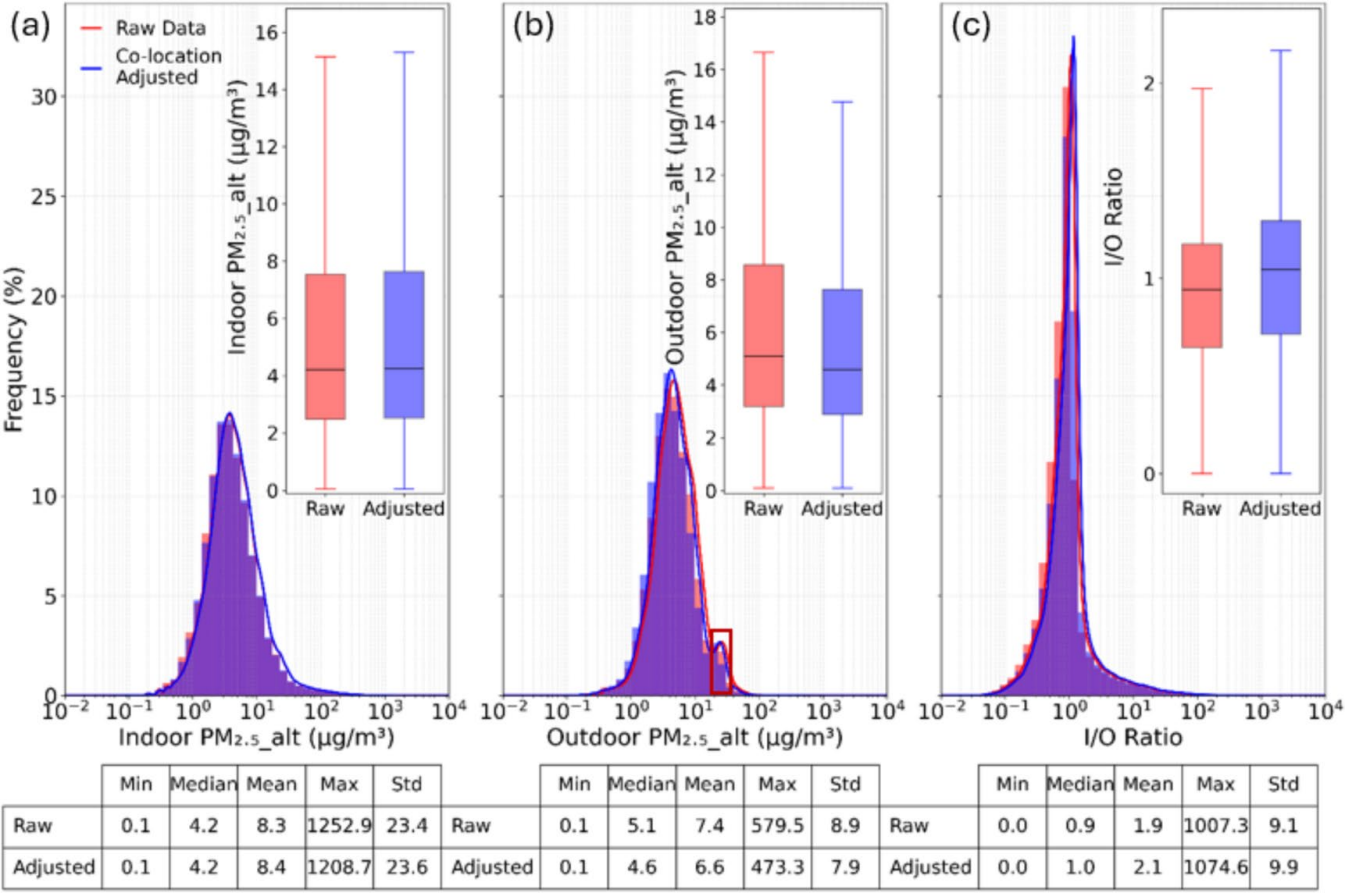
Distributions of indoor and outdoor PM_2.5__alt measurements and concurrent PM_2.5__alt indoor/outdoor (I/O) ratios measured in the field study, comparing raw (without co-location factors) and adjusted (with co-location factors). Box plots exclude outliers for visual clarity

Histograms and boxplots of PM_2.5__alt data collected from indoor and outdoor monitors in the field study, shown separately, along with the corresponding calculated I/O ratios, are shown in Fig. 9 to compare their distributions between raw concentrations and those with co-location factors from laboratory co-location testing applied (and also Figures S7 and S8).

Across all three comparisons, there are only minimal shifts on the distributions when applying the co-location factors. There were essentially no differences in the median, mean, and standard deviation between raw and adjusted indoor PM_2.5__alt concentrations (Fig. 9a). The median, mean, and standard deviation of adjusted outdoor PM_2.5__alt concentrations were each approximately 10–11% lower than raw concentrations (Fig. 9b). I/O ratios in both data sets spanned a wide range, from ~ 0 to > 1000 for individual data points at 10-min time intervals, while the median, mean, and standard deviation were similar between raw and adjusted datasets: median I/O = 0.9 raw and 1.0 adjusted; mean I/O = 1.9 raw and 2.1 adjusted; and standard deviation of I/O = 9.1 raw and 9.9 adjusted. The magnitudes of these effects were negligible, however, with Cohen’s *d* of − 0.004 for indoor, 0.0094 for outdoor, and -0.002 for I/O ratios. All values indicate that application of the co-location factors did not introduce significant impacts on the distributions of these values from this field study. For context, I/O ratios > 1 for PM_2.5_ are not uncommon in residences, given the abundance of indoor sources that can be present in homes (Chen and Zhao 2011). To the extent that local outdoor PM_2.5__alt readings can represent PM_2.5_ mass concentrations, median (mean) ambient concentrations of ~ 5 (~ 7) µg/m^3^ are fairly low for the area, which is historically subject to ambient air pollution from agricultural dust and biomass burning, including agricultural burning and wildfires (Clausnitzer and Singer 1996; Nieuwenhuijsen et al. 1998; Nieuwenhuijsen and Schenker 1998; Cisneros et al. 2017; Flores-Landeros et al. 2022; Sun et al. 2022). However, this is consistent with the San Joaquin Valley recently achieving a milestone of meeting federal ambient air quality standards for PM_2.5_ for 2022–2024 (Valley Air District 2025). Wildfires, although historically common in the area, where uncommon during our field deployments. In fact, there is only a small perturbation in the ambient PM_2.5__alt distribution visible in Fig. 9b, representing a small cluster of ambient air pollution events.

Since PA-II monitors contain two independently operating optical particle sensors next to each other, theoretically, the two sensors should have identical readings. In terms of data quality assurance, a comparison of the difference in data from the two sensors can be used as an inclusion/exclusion criterion, for example, differing by no more than a defined threshold. Figure S9, Tables S7, and S8 explore the imprecision of PM_2.5__alt readings between channels a and b for each monitor in both the laboratory co-locations and the field data collection campaigns, comparing the absolute difference between a and b channels and divided by the sum of a and b channels at each collected timestamp (Wallace 2022). Channel a/b imprecision was similar between both data sets, with a median of medians for 50 monitors in the laboratory co-location of 3.6% (ranging 2%–24.1%, SD = 0.04) and 3.2% (ranging 1.5% and 11.1%, SD = 0.02) in the field campaign, although the distributions of imprecision values were significantly different between co-location measurements and the field measurements for each monitor (p < 0.05 from KS test). Among the 50 monitors compared, imprecision for the field-collected data was lower than the laboratory co-location data for 35 of the monitors and higher for 15 of the monitors. Applying a precision limit of 20%, a total of 6.8% of the laboratory co-location data exceeded this limit compared to only 2.9% of the field-collected data. Higher imprecision with laboratory co-location data is attributable to lower concentrations during co-location testing compared to field-testing.

Finally, some previous studies have noted that PurpleAir and other optical particle monitors overestimate PM concentrations at high relative humidity (RH) conditions, especially above 60% RH, and may need algorithmic adjustments at high RH levels (Chakrabarti et al. 2004; Mathieu-Campbell et al. 2024). However, given the dry climate of our field study locations, over 98% of data was collected with indoor RH below 60%, such that additional adjustment for high RH was not needed.

### Additional Lessons Learned and Limitations

We learned several other lessons throughout these laboratory co-location and field measurement experiences. For example, we learned that caution needs to be exercised when removing the microSD card from the PA-II-SD monitor to connect to a microSD reader to download data, as the microSD card is wedged into the PA-II-SD monitor housing and is difficult to both remove and reinstall, especially in field settings. We found it ideal to carry small hand tools such as tongs or tweezers in the field to help remove and reinsert the microSD cards from the PA-II-SD monitors. Otherwise, the microSD card can easily become lost inside the PA monitor by slipping or detaching, requiring field personnel to shake the monitor upside down to dislodge the microSD card. Moreover, once the microSD card is removed, it can easily get lost in carpeting or furnishings, especially in low light or cluttered field settings. And the process of re-inserting the microSD card into the reader can physically damage the device and prevent it from further data recordings because the hardware is somewhat fragile. Another alternative approach for data retrieval that may be applicable in some settings is to clearly mark PA-II-SD monitors, remove them from the field, and remove the microSD card in an office or laboratory setting where better light and tools are more readily available and chances of mistakes are lower. However, if the goal is to leave monitors deployed while extracting interim data, this approach may not be practical.

One limitation is that the monitor performance results presented herein are limited to the specific batches of monitors used, while performance may vary depending on the production run. For example, a significant data quality issue arose during the initial field-testing phase of the project. In 2021, there was a re-design of the Plantower sensors inside the PurpleAir PA-II monitors. The monitors appeared the same externally but contained a new microcontroller in the sensors. The readings from these monitors differed from all other existing PA-II monitors in the smaller particle size bins by 50% or more. Most notably, counts in the smallest particle size bin (0.3 µm) were approximately threefold lower compared to readings from other PA-II monitors. Because wildfire smoke consists primarily of fine particles, and those smallest particles are of greatest importance for human health, the reduction in sensitivity to small particles was deemed unacceptable for the aims of the FRESSCA study. We became aware of this potential issue in early-July 2022 in conversations with scientists at the U.S. Environmental Protection Agency. We immediately contacted PurpleAir and were informed that 100% of the monitors in the batch we received from them in the Spring of 2022 had the problematic sensor. All of the affected monitors had already been installed by our team in the communities in April and May 2022. In response to our issues, PurpleAir provided us with a shipment of new monitors that contained the more sensitive (original) Plantower sensor, and we conducted additional field visits to all the participant homes to co-locate the newly received monitors next to the older monitors inside participant homes. We subsequently collected the defective monitors and shipped them back to PurpleAir.

Overall, this work seeks to leverage our experiences and provide practical guidance, resources, and tools for using low-cost PurpleAir particle monitors in residential field studies of indoor and outdoor air. While our experiences focused on PA-II and PA-II-SD monitors installed inside and outside homes in communities of predominantly agricultural workers in California’s Central Valley in the U.S., our work can also inform studies in any global region that faces practical considerations for monitor deployment, quality assurance and quality control, data retrieval, and data usability. For example, the proliferation of low-cost air quality monitors—including PurpleAir and many other makes and models—has greatly increased the spatiotemporal scales at which indoor and outdoor exposure measurements can be conducted. Some researchers have utilized extensive calibration approaches (conducted before, during, or after deployment) when deploying batches of lower-cost, consumer-grade particle monitors such as PurpleAir (Campmier et al. 2023; Koehler et al. 2023; Sonntag et al. 2023; Couzo et al. 2024; Aune et al. 2025), while others have not (Awokola et al. 2022; Ferro et al. 2022; Johnson et al. 2024; Krakowka et al. 2024). The findings of this study are also important given ongoing civic initiatives to deploy low-cost indoor and outdoor air quality sensors at scale across communities (O’Dell et al. 2023). Results from our co-location testing and our applications of co-location factors to field-collected indoor and outdoor data suggest that for lower-cost monitors such as PA-II that have demonstrated good performance in other environments, co-location testing is probably most valuable for identifying problematic monitors while calibration against reference aerosols may not always be necessary, depending on application requirements.

## Conclusion

This work contributes to the growing body of knowledge on low-cost particle sensor performance and usability by providing practical guidance learned from our recent experiences with PurpleAir PA-II monitors deployed in a recent field study of residential indoor and outdoor air. Laboratory co-location measurements demonstrated that > 90% of the tested PA-II and PA-II-SD monitors performed very well relative to each other within raw PM_2.5__alt concentrations of 0 to 28 µg/m^3^, with only a few problematic monitors identified that warranted further investigation or exclusion from use. Further, application of co-location factors to field-collected data did not significantly affect distributions of PM_2.5__alt concentrations when using the mean of all co-located monitors as a reference, which suggests that while co-location prior to deployment can be useful for improving data collection success and flagging poorly performing monitors, it may not be necessary for all applications. Our field experiences in these residential and outdoor settings revealed that relying solely on Wi-Fi data transmission can result in large data loss and that by using microSD card storage with PA-II-SD monitors effectively doubled the data collection success rate in these settings. We also identified and discussed several practical challenges and solutions for field deployments, including the challenges of harmonizing data in different structures, the importance of careful handling of microSD cards during data retrieval, and considerations for appropriate indoor mounting solutions. Our recommendations seek to help researchers maximize data quality and completeness while minimizing resource expenditure in future field studies.

## Supplementary Information

Below is the link to the electronic supplementary material.

Supplementary file1 (DOCX 5224 KB)

## Data Availability

Portions of the data from this project are posted to a public repository, Open Science Framework (OSF): https://osf.io/arz3d. Additional data not posted to the repository may be available upon reasonable request.

## References

[CR1] Ali AS (2015) Open Source Building Science Sensors (OSBSS): A low-cost Arduino-based platform for long-term data collection in indoor environments (Master’s thesis). Illinois Institute of Technology, Chicago, IL

[CR2] Ardon-Dryer K, Dryer Y, Williams JN, Moghimi N (2020) Measurements of PM2.5 with PurpleAir under atmospheric conditions. Atmos Meas Tech 13:5441–5458. 10.5194/amt-13-5441-2020

[CR3] Ashworth, B., 2023. It’s Easy to Check the Air Quality. Meet the People Collecting That Data for You [WWW Document]. Wired. https://www.wired.com/story/the-people-collecting-air-quality-sensor-data/

[CR4] Aune KT, Wilks M, Green T, Rule AM, McCormack M, Hansel NN, Putcha N, Kirk G, Raju S, Koehler K (2025) Calibration of indoor temperature and relative humidity readings in the PurpleAir monitor. Environ Monit Assess 197:667. 10.1007/s10661-025-14076-5 40402320 10.1007/s10661-025-14076-5

[CR5] Awokola B, Okello G, Johnson O, Dobson R, Ouédraogo AR, Dibba B, Ngahane M, Ndukwu C, Agunwa C, Marangu D, Lawin H, Ogugua I, Eze J, Nwosu N, Ofiaeli O, Ubuane P, Osman R, Awokola E, Erhart A, Mortimer K, Jewell C, Semple S (2022) Longitudinal ambient PM2.5 measurement at fifteen locations in eight sub-Saharan African countries using low-cost sensors. Atmosphere 13:1593. 10.3390/atmos13101593

[CR6] Barkjohn KK, Gantt B, Clements AL (2021) Development and application of a United States-wide correction for PM2.5 data collected with the PurpleAir sensor. Atmos Meas Tech 14:4617–4637. 10.5194/amt-14-4617-2021 10.5194/amt-14-4617-2021 PMC8422884 34504625

[CR7] Campmier MJ, Gingrich J, Singh S, Baig N, Gani S, Upadhya A, Agrawal P, Kushwaha M, Mishra HR, Pillarisetti A, Vakacherla S, Pathak RK, Apte JS (2023) Seasonally optimized calibrations improve low-cost sensor performance: long-term field evaluation of PurpleAir sensors in urban and rural India. Atmos Meas Tech 16:4357–4374. 10.5194/amt-16-4357-2023

[CR8] Chakrabarti B, Fine PM, Delfino R, Sioutas C (2004) Performance evaluation of the active-flow personal DataRAM PM2.5 mass monitor (Thermo Anderson pDR-1200) designed for continuous personal exposure measurements. Atmos Environ 38:3329–3340. 10.1016/j.atmosenv.2004.03.007

[CR9] Chen C, Zhao B (2011) Review of relationship between indoor and outdoor particles: I/O ratio, infiltration factor and penetration factor. Atmos Environ 45:275–288. 10.1016/j.atmosenv.2010.09.048

[CR10] Cisneros R, Brown P, Cameron L, Gaab E, Gonzalez M, Ramondt S, Veloz D, Song A, Schweizer D (2017) Understanding public views about air quality and air pollution sources in the San Joaquin Valley, California. J Environ Public Health 2017:1–7. 10.1155/2017/4535142 10.1155/2017/4535142 PMC5392406 28469673

[CR11] Clausnitzer H, Singer MJ (1996) Respirable-dust production from agricultural operations in the Sacramento Valley, California. J Environ Qual 25:877–884. 10.2134/jeq1996.00472425002500040032x

[CR12] Cohen, J., 2013. Statistical power analysis for the behavioral sciences, 0 ed. Routledge. 10.4324/9780203771587

[CR13] Collier-Oxandale A, Feenstra B, Papapostolou V, Polidori A (2022) Airsensor v1.0: enhancements to the open-source R package to enable deep understanding of the long-term performance and reliability of PurpleAir sensors. Environ Model Softw 148:105256. 10.1016/j.envsoft.2021.105256

[CR14] Couzo E, Valencia A, Gittis P (2024) Evaluation and correction of PurpleAir temperature and relative humidity measurements. Atmosphere 15:415. 10.3390/atmos15040415

[CR15] Datta A, Saha A, Zamora ML, Buehler C, Hao L, Xiong F, Gentner DR, Koehler K (2020) Statistical field calibration of a low-cost PM2.5 monitoring network in Baltimore. Atmos Environ 242:117761. 10.1016/j.atmosenv.2020.117761 10.1016/j.atmosenv.2020.117761 PMC7480820 32922146

[CR16] deSouza P, Kinney PL (2021) On the distribution of low-cost PM2.5 sensors in the US: demographic and air quality associations. J Expo Sci Environ Epidemiol. 10.1038/s41370-021-00328-2 10.1038/s41370-021-00328-2 33958706

[CR17] deSouza P, Kahn R, Stockman T, Obermann W, Crawford B, Wang A, Crooks J, Li J, Kinney P (2022) Calibrating networks of low-cost air quality sensors. Atmos Meas Tech 15:6309–6328. 10.5194/amt-15-6309-2022

[CR18] deSouza P, Barkjohn K, Clements A, Lee J, Kahn R, Crawford B, Kinney P (2023) An analysis of degradation in low-cost particulate matter sensors. Environ Sci: Atmos 3:521–536. 10.1039/D2EA00142J 37234229 10.1039/d2ea00142j PMC10208317

[CR19] Durkin A, Gonzalez R, Isaksen TB, Walker E, Errett NA (2020) Establishing a community air monitoring network in a wildfire smoke-prone rural community: the motivations, experiences, challenges, and ideas of clean Air Methow’s clean air ambassadors. IJERPH 17:8393. 10.3390/ijerph17228393 33202742 10.3390/ijerph17228393 PMC7697345

[CR78] Fang R, Zhang Y, Collingwood S, Stanford JB, Porucznik C, Sleeth D (2025) Assessing the impact of real-world environmental factors on low-cost PM2.5 monitor performance by comparing calibration before and after deployment. Sci Total Environ 995:180106. 10.1016/j.scitotenv.2025.180106 40700906 10.1016/j.scitotenv.2025.180106

[CR76] Farhoodi S, Kang K, Jagota K, Karpen N, Elfessi ZZ, Rubinstein I, Heidarinejad M, Stephens B (2025) Analysis of sources and sinks of indoor particulate matter reveals insights into the real-world efficacy of portable air cleaners in a randomized intervention trial. Sci Total Environ 996:180136. 10.1016/j.scitotenv.2025.180136 40737779 10.1016/j.scitotenv.2025.180136 PMC13224824

[CR20] Ferro AR, Zíková N, Masiol M, Satsangi GP, Twomey T, Chalupa DC, Rich DQ, Hopke PK (2022) Residential indoor and outdoor PM measured using low-cost monitors during the heating season in Monroe County, NY. Aerosol Air Qual Res 22:220210. 10.4209/aaqr.220210

[CR21] Flores-Landeros H, Pells C, Campos-Martinez MS, Fernandez-Bou AS, Ortiz-Partida JP, Medellín-Azuara J (2022) Community perspectives and environmental justice in California’s San Joaquin Valley. Environ Justice 15:337–345. 10.1089/env.2021.0005

[CR77] Francisco PW, Gilleade K, Sun Y, Merrin Z, Alavy M, LaFleur J (2025) Measured impacts of supply vs. exhaust ventilation in residences. Indoor Environ 2(2):100093. 10.1016/j.indenv.2025.100093

[CR22] Giordano MR, Malings C, Pandis SN, Presto AA, McNeill VF, Westervelt DM, Beekmann M, Subramanian R (2021) From low-cost sensors to high-quality data: a summary of challenges and best practices for effectively calibrating low-cost particulate matter mass sensors. J Aerosol Sci 158:105833. 10.1016/j.jaerosci.2021.105833

[CR23] Jayaratne, R., Liu, X., Ahn, K.-H., Asumadu-Sakyi, A., Fisher, G., Gao, J., Mabon, A., Mazaheri, M., Mullins, B., Nyaku, M., Ristovski, Z., Scorgie, Y., Thai, P., Dunbabin, M., Morawska, L., 2020. Low-cost PM2.5 Sensors: An Assessment of Their Suitability for Various Applications. Aerosol Air Qual. Res. 10.4209/aaqr.2018.10.0390

[CR24] Johnson MA, Abuya T, Wickramanayake A, Miller H, Sambu D, Mwanga D, Odwe G, Ndwiga C, Piedrahita R, Rossanese M, Gatari MJ, Giordano MR, Westervelt DM, Wotton L, Rajasekharan S (2024) Patterns and drivers of maternal personal exposure to PM_2.5_ in informal settlements in Nairobi, Kenya. Environ Sci Atmos 4:578–591. 10.1039/D3EA00074E

[CR25] Kang I, Wang M, Stephens B (2025) Letter to the editor regarding estimates in “Indoor air quality impacts of residential mechanical ventilation system retrofits in existing homes in Chicago, IL” (2022) Sci Tot Environ 804:150129. Sci Total Environ 963:178289. 10.1016/j.scitotenv.2024.178289 39824089 10.1016/j.scitotenv.2024.178289

[CR26] Koehler K, Wilks M, Green T, Rule AM, Zamora ML, Buehler C, Datta A, Gentner DR, Putcha N, Hansel NN, Kirk GD, Raju S, McCormack M (2023) Evaluation of calibration approaches for indoor deployments of PurpleAir monitors. Atmos Environ 310:119944. 10.1016/j.atmosenv.2023.119944 10.1016/j.atmosenv.2023.119944 PMC10609655 37901719

[CR27] Krakowka WI, Luo J, Craver A, Pinto JM, Ahsan H, Olopade CS, Aschebrook-Kilfoy B (2024) Household air pollution disparities between socioeconomic groups in Chicago. Environ Res Commun 6:091002. 10.1088/2515-7620/ad6d3f 39238838 10.1088/2515-7620/ad6d3f PMC11373614

[CR28] Levy Zamora M, Xiong F, Gentner D, Kerkez B, Kohrman-Glaser J, Koehler K (2019) Field and laboratory evaluations of the low-cost Plantower particulate matter sensor. Environ Sci Technol 53:838–849. 10.1021/acs.est.8b05174 30563344 10.1021/acs.est.8b05174

[CR29] Li J, Mattewal SK, Patel S, Biswas P (2020) Evaluation of nine low-cost-sensor-based particulate matter monitors. Aerosol Air Qual Res 20:254–270. 10.4209/aaqr.2018.12.0485

[CR30] Liang L, Daniels J (2022) What influences low-cost sensor data calibration? - a systematic assessment of algorithms, duration, and predictor selection. Aerosol Air Qual Res 22:220076. 10.4209/aaqr.220076

[CR31] Liang Y, Sengupta D, Campmier MJ, Lunderberg DM, Apte JS, Goldstein AH (2021) Wildfire smoke impacts on indoor air quality assessed using crowdsourced data in California. Proc Natl Acad Sci U S A 118:e2106478118. 10.1073/pnas.2106478118 34465624 10.1073/pnas.2106478118 PMC8433518

[CR32] Lunderberg DM, Liang Y, Singer BC, Apte JS, Nazaroff WW, Goldstein AH (2023) Assessing residential PM _2.5_ concentrations and infiltration factors with high spatiotemporal resolution using crowdsourced sensors. Proc Natl Acad Sci U S A 120:e2308832120. 10.1073/pnas.2308832120 38048461 10.1073/pnas.2308832120 PMC10723120

[CR33] Mathieu-Campbell ME, Guo C, Grieshop AP, Richmond-Bryant J (2024) Calibration of PurpleAir low-cost particulate matter sensors: model development for air quality under high relative humidity conditions. Atmos Meas Tech 17:6735–6749. 10.5194/amt-17-6735-2024 40078349 10.5194/amt-17-6735-2024 PMC11900072

[CR34] Molina Rueda E, Carter E, L’Orange C, Quinn C, Volckens J (2023) Size-resolved field performance of low-cost sensors for particulate matter air pollution. Environ Sci Technol Lett 10:247–253. 10.1021/acs.estlett.3c00030 36938150 10.1021/acs.estlett.3c00030 PMC10018765

[CR35] Morawska L, Thai PK, Liu X, Asumadu-Sakyi A, Ayoko G, Bartonova A, Bedini A, Chai F, Christensen B, Dunbabin M, Gao J, Hagler GSW, Jayaratne R, Kumar P, Lau AKH, Louie PKK, Mazaheri M, Ning Z, Motta N, Mullins B, Rahman MM, Ristovski Z, Shafiei M, Tjondronegoro D, Westerdahl D, Williams R (2018) Applications of low-cost sensing technologies for air quality monitoring and exposure assessment: How far have they gone? Environ Int 116:286–299. 10.1016/j.envint.2018.04.018 29704807 10.1016/j.envint.2018.04.018 PMC6145068

[CR36] Mousavi A, Wu J (2021) Indoor-generated PM _2.5_ during COVID-19 shutdowns across California: application of the PurpleAir indoor-outdoor low-cost sensor network. Environ Sci Technol 55:5648–5656. 10.1021/acs.est.0c06937 33871991 10.1021/acs.est.0c06937 PMC9033533

[CR37] Mousavi A, Yuan Y, Masri S, Barta G, Wu J (2021) Impact of 4th of July fireworks on spatiotemporal PM2.5 concentrations in California based on the PurpleAir sensor network: implications for policy and environmental justice. IJERPH 18:5735. 10.3390/ijerph18115735 34071796 10.3390/ijerph18115735 PMC8198140

[CR38] Mullen C, Flores A, Grineski S, Collins T (2022) Exploring the distributional environmental justice implications of an air quality monitoring network in Los Angeles County. Environ Res 206:112612. 10.1016/j.envres.2021.112612 34953883 10.1016/j.envres.2021.112612

[CR39] Nieuwenhuijsen MJ, Schenker MB (1998) Determinants of personal dust exposure during field crop operations in California agriculture. Am Ind Hyg Assoc J 59:9–13. 10.1080/15428119891010271 9438330 10.1080/15428119891010271

[CR40] Nieuwenhuijsen MJ, Kruize H, Schenker MB (1998) Exposure to dust and its particle size distribution in California agriculture. Am Ind Hyg Assoc J 59:34–38. 10.1080/15428119891010316 9438333 10.1080/15428119891010316

[CR41] O’Dell, K., Ford, B., Burkhardt, J., Magzamen, S., Anenberg, S.C., Bayham, J., Fischer, E.V., Pierce, J.R., 2023. Outside in: the relationship between indoor and outdoor particulate air quality during wildfire smoke events in western US cities. Environ. Res.: Health 1, 015003. 10.1088/2752-5309/ac7d69

[CR42] Park S, Lee S, Yeo M, Rim D (2023) Field and laboratory evaluation of PurpleAir low-cost aerosol sensors in monitoring indoor airborne particles. Build Environ 234:110127. 10.1016/j.buildenv.2023.110127

[CR43] Prathibha P, Turner M, Wei L, Davis A, Vinsonhaler K, Batchelder A, McCaughey B, Carlstad J, Chelminski A, Rappold AG, Hassett-Sipple B, Holder A (2024) Usage and impact of a do-it-yourself air cleaner on residential PM2.5 in a smoke-impacted community. Atmos Environ. 10.1016/j.atmosenv.2024.120650 10.1016/j.heliyon.2024.e25225 PMC10875335 38375293

[CR44] PurpleAir, 2023a. PurpleAir’s New API Dashboard & Data Download Tool Release [WWW Document]. PurpleAir. https://www2.purpleair.com/blogs/blog-home/purpleair-s-new-api-dashboard-data-download-tool-release

[CR45] PurpleAir, 2023b. SD Card Logging and Troubleshooting [WWW Document]. PurpleAir Community. https://community.purpleair.com/t/sd-card-logging-and-troubleshooting/543

[CR46] Robinson DL, Goodman N, Vardoulakis S (2023) Five years of accurate PM2.5 measurements demonstrate the value of low-cost PurpleAir monitors in areas affected by woodsmoke. IJERPH 20:7127. 10.3390/ijerph20237127 38063557 10.3390/ijerph20237127 PMC10706150

[CR47] S S, Agrawal P, Kulkarni P, Gautam HC, Kushwaha M, Sreekanth V (2023) Multiple PM low-cost sensors, multiple seasons’ data, and multiple calibration models. Aerosol Air Qual Res 23:220428. 10.4209/aaqr.220428

[CR48] Sá JP, Alvim-Ferraz MCM, Martins FG, Sousa SIV (2022) Application of the low-cost sensing technology for indoor air quality monitoring: a review. Environ Technol Innov 28:102551. 10.1016/j.eti.2022.102551

[CR49] Sankhyan S, Witteman JK, Coyan S, Patel S, Vance ME (2022) Assessment of PM _2.5_ concentrations, transport, and mitigation in indoor environments using low-cost air quality monitors and a portable air cleaner. Environ Sci Atmos 2:647–658. 10.1039/D2EA00025C

[CR50] Sayahi T, Butterfield A, Kelly KE (2019) Long-term field evaluation of the Plantower PMS low-cost particulate matter sensors. Environ Pollut 245:932–940. 10.1016/j.envpol.2018.11.065 30682749 10.1016/j.envpol.2018.11.065

[CR51] SCAQMD, 2020. PurpleAir PA-I-Indoor [WWW Document]. AQ-SPEC. https://www.aqmd.gov/aq-spec/sensordetail/purpleair-pa-i-indoor

[CR52] SCAQMD, 2023. PurpleAir PA-II. Air Quality Sensor Performance Evaluation Center (AQ-SPEC). https://www.aqmd.gov/aq-spec/product/purpleair-pa-ii

[CR53] Searle N, Kaur K, Kelly K (2023) Technical note: identifying a performance change in the Plantower PMS 5003 particulate matter sensor. J Aerosol Sci 174:106256. 10.1016/j.jaerosci.2023.106256

[CR54] Singer BC, Delp WW (2018) Response of consumer and research grade indoor air quality monitors to residential sources of fine particles. Indoor Air 28:624–639. 10.1111/ina.12463 29683219 10.1111/ina.12463

[CR55] Singh A, Stephens B, Heidarinejad M, Stinson B, Gall E, Wagner J, Singer B, Miller S, Martinez N, Rodriguez R, Solomon G (2025) Development and laboratory evaluation of a do-it-yourself (DIY) filtration solution for residential evaporative coolers to reduce indoor wildfire smoke exposure. Build Environ 270:112475. 10.1016/j.buildenv.2024.112475

[CR56] Solomon, G., Martinez, N., Von Behren, J., Kaser, I., Chang, D., Singh, A., Jarmul, S., Miller, S., Reynolds, P., Heidarinejad, M., Stephens, B., Singer, B., Wagner, J., Balmes, J., 2025. Evaporative Coolers and Wildfire Smoke Exposure: A Climate Justice Issue in Hot, Dry Regions Authors. Frontiers in Public Health 13. 10.3389/fpubh.2025.1541053 10.3389/fpubh.2025.1541053 PMC11897565 40078771

[CR57] Sonntag DB, Jung H, Harline RP, Peterson TC, Willis SE, Christensen TR, Johnston JD (2023) Infiltration of outdoor PM2.5 pollution into homes with evaporative coolers in Utah County. Sustainability 16(1):177. 10.3390/su16010177

[CR58] Stampfer O, Zuidema C, Allen RW, Fox J, Sampson P, Seto E, Karr CJ (2024) Practical considerations for using low-cost sensors to assess wildfire smoke exposure in school and childcare settings. J Expo Sci Environ Epidemiol. 10.1038/s41370-024-00677-8 10.1038/s41370-024-00677-8 PMC11550266 38730039

[CR75] Stephens B, Kang I, Jagota K, Elfessi Z, Karpen K, Farhoodi S, Heidarinejad M, Rubinstein I (2025) Study protocol for a 1-year randomized single-blind parallel group trial of stand-alone indoor air filtration in the homes of US military Veterans with moderate to severe COPD in metropolitan Chicago. Trials 26(1). 10.1186/s13063-025-08880-0 10.1186/s13063-025-08880-0 PMC12139193 40468409

[CR59] Sun P, Farley RN, Li L, Srivastava D, Niedek CR, Li J, Wang N, Cappa CD, Pusede SE, Yu Z, Croteau P, Zhang Q (2022) PM2.5 composition and sources in the San Joaquin Valley of California: a long-term study using ToF-ACSM with the capture vaporizer. Environ Pollut 292:118254. 10.1016/j.envpol.2021.118254 34610412 10.1016/j.envpol.2021.118254

[CR60] Tryner J, L’Orange C, Mehaffy J, Miller-Lionberg D, Hofstetter JC, Wilson A, Volckens J (2020) Laboratory evaluation of low-cost PurpleAir PM monitors and in-field correction using co-located portable filter samplers. Atmos Environ 220:117067. 10.1016/j.atmosenv.2019.117067

[CR61] Tsameret S, Furuta D, Saha P, Kwak N, Hauryliuk A, Li X, Presto AA, Li J (2024) Low-cost indoor sensor deployment for predicting PM _2.5_ exposure. ACS EST Air. 10.1021/acsestair.3c00105 10.1021/acsestair.3c00105 PMC11321336 39144754

[CR62] Valley Air District, 2025. Recent Air Quality Achievements [WWW Document]. San Joaquin Valley Air Pollution Control District. https://ww2.valleyair.org/about/achievements/recent-air-quality-achievements

[CR63] Wallace L (2022) Intercomparison of PurpleAir sensor performance over three years indoors and outdoors at a home: bias, precision, and limit of detection using an improved algorithm for calculating PM2.5. Sensors (Basel) 22:2755. 10.3390/s22072755 35408369 10.3390/s22072755 PMC9002513

[CR64] Wallace L (2023) Cracking the code—matching a proprietary algorithm for a low-cost sensor measuring PM1 and PM2.5. Sci Total Environ 893:164874. 10.1016/j.scitotenv.2023.164874 37336395 10.1016/j.scitotenv.2023.164874

[CR65] Wallace L (2024) Socioeconomic inequity of measured indoor and outdoor exposure to PM2.5: 5 years of data from 14,000 low-cost particle monitors. Indoor Environ 1:100016. 10.1016/j.indenv.2024.100016

[CR66] Wallace L, Ott W (2023) Long-term indoor-outdoor PM2.5 measurements using PurpleAir sensors: an improved method of calculating indoor-generated and outdoor-infiltrated contributions to potential indoor exposure. Sensors 23:1160. 10.3390/s23031160 36772199 10.3390/s23031160 PMC9920798

[CR67] Wallace L, Ott W, Zhao T, Cheng K-C, Hildemann L (2020) Secondhand exposure from vaping marijuana: concentrations, emissions, and exposures determined using both research-grade and low-cost monitors. Atmosp Environ 8:100093. 10.1016/j.aeaoa.2020.100093

[CR68] Wallace L, Bi J, Ott WR, Sarnat J, Liu Y (2021) Calibration of low-cost PurpleAir outdoor monitors using an improved method of calculating PM. Atmos Environ 256:118432. 10.1016/j.atmosenv.2021.118432

[CR69] Wallace, L., Zhao, T., Klepeis, N.E., 2022. Calibration of PurpleAir PA-I and PA-II Monitors Using Daily Mean PM2.5 Concentrations Measured in California, Washington, and Oregon from 2017 to 2021. Sensors 22, 4741. 10.3390/s22134741 10.3390/s22134741 PMC9269269 35808235

[CR70] Wallace, L.A., Zhao, T., Klepeis, N.E., 2022. Indoor contribution to PM_2.5_ exposure using all PurpleAir sites in Washington, Oregon, and California. Indoor Air 32. 10.1111/ina.13105 10.1111/ina.13105 36168225

[CR71] Xiang J, Huang C-H, Shirai J, Liu Y, Carmona N, Zuidema C, Austin E, Gould T, Larson T, Seto E (2021) Field measurements of PM2.5 infiltration factor and portable air cleaner effectiveness during wildfire episodes in US residences. Sci Total Environ 773:145642. 10.1016/j.scitotenv.2021.145642 33592483 10.1016/j.scitotenv.2021.145642 PMC8026580

[CR72] Zimmerman N (2022) Tutorial: guidelines for implementing low-cost sensor networks for aerosol monitoring. J Aerosol Sci 159:105872. 10.1016/j.jaerosci.2021.105872

[CR73] Zou Y, Young M, Chen J, Liu J, May A, Clark JD (2020a) Examining the functional range of commercially available low-cost airborne particle sensors and consequences for monitoring of indoor air quality in residences. Indoor Air 30:213–234. 10.1111/ina.12621 31709614 10.1111/ina.12621

[CR74] Zou Y, Young M, Wickey M, May A, Clark JD (2020b) Response of eight low-cost particle sensors and consumer devices to typical indoor emission events in a real home (ASHRAE 1756-RP). Sci Technol Built Environ 26:237–249. 10.1080/23744731.2019.1676094

