# Supplementary material for: Practical Guidance for Using PurpleAir Particle Monitors for Indoor and Outdoor Measurements in Community Field Studies
Source: Aerosol Air Qual Res. 2025 Aug 11;25(8):47. doi: 10.1007/s44408-025-00048-4 (PMC12339596; doi:10.1007/s44408-025-00048-4)
Supplement: Supplementary file 1 — Supplementary file1 (DOCX 5224 KB) [file 44408_2025_48_MOESM1_ESM.docx]

Supplemental Material for:

**Practical guidance for using PurpleAir particle monitors for indoor and outdoor measurements in community field studies**

Mingyu Wang^1^, David Chang^2^, Aditya Singh^1^, Jeff Wagner^3^, Zhong-Min Wang^3^, Brett C. Singer^4^, Shelly L. Miller^5^, Nayamin Martinez^6^, Ruben Rodriguez^6^, Isabella Kaser^2^, McKenna Thompson^7^, Mohammad Heidarinejad^1^, Brent Stephens^1^*, Gina Solomon^8,9^

^1^ Department of Civil, Architectural, and Environmental Engineering, Illinois Institute of Technology, Chicago, IL USA

^2^ Tracking California, Public Health Institute, Oakland, CA USA

^3^ California Department of Public Health, Center for Laboratory Sciences, Environmental Health Laboratory, Richmond, CA, USA

^4^ Lawrence Berkley National Laboratory, Berkeley, CA USA

^5^ Department of Mechanical Engineering, University of Colorado, Boulder, CO USA

^6^ Central California Environmental Justice Network, Fresno, CA USA

^7^ Office of Environmental Health Hazard Assessment, California Environmental Protection Agency, Oakland, CA USA

^8^ Public Health Institute, Oakland, CA USA

^9^ School of Medicine, University of California, San Francisco, CA USA

*Corresponding author:

Brent Stephens, PhD

Professor and Department Chair

Arthur W. Hill Endowed Chair in Sustainability

Department of Civil, Architectural, and Environmental Engineering

Illinois Institute of Technology

Alumni Memorial Hall Room 228

3201 S Dearborn Street

Chicago, IL 60616 USA

# 1. FRESSCA Study Locations

The field measurement campaigns were conducted in California’s San Joaquin Valley (SJV), including in Fresno (Coalinga and Huron), Kings (Kettleman City), and Kern (Arvin, Lamont, and Lost Hills) Counties. **Figure S1** shows the map of the FRESSCA study locations.

**
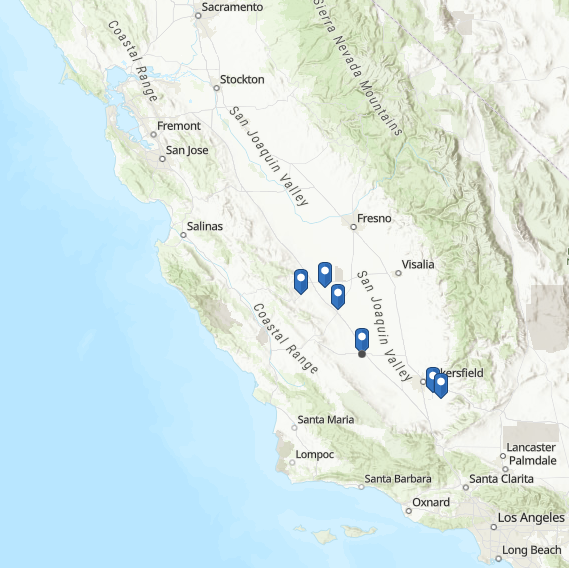
**

**Figure S1.** Map of the FRESSCA study locations, California, 2022–2023. This figure is replicated from (Solomon et al., 2025).

# 2. Costs of Wi-Fi data extraction via API calling

The tiered PurpleAir API points-based pricing structure as of April 2025 is summarized in **Table S1**.^[[1]](#footnote-2)^ We use this points-based pricing structure to illustrate data download costs for several scenarios, although we did not have to pay these costs in our field study.

Table S1. PurpleAir API points-based pricing structure, as of April 2025

| **Purchase**  **Amount** | **Points Per USD (points/$)** |
| --- | --- |
| $10 - $49 | 100,000 |
| $50 - $249 | 150,000 |
| $250 - $499 | 200,000 |
| $500 - $999 | 300,000 |
| $1,000 - $4,999 | 500,000 |
| $5,000+ | 1,000,000 |

Previously, the PurpleAir API data download approach allowed a limited number of data points to be downloaded in a single request, necessitating scripting of repeated downloads. In May 2024, the API began to allow downloading more data points at a time, which reduces the level of effort and time needed to download data. **Table S2** summarizes these data download request limits.

Table S2. PurpleAir API data download request limits

| **Average Period** | **Previous Limit**  **(Before May 1, 2024)** | **New Limit**  **(After May 1, 2024)** | **Relative Increase** |
| --- | --- | --- | --- |
| Real-Time | 2 Days | 30 Days | 15x |
| 10 Minutes | 3 Days | 60 Days | 20x |
| 30 Minutes | 7 Days | 90 Days | 12x |
| 1 Hour | 14 Days | 180 Days | 12x |
| 6 Hours | 90 Days | 1 Year | 4x |
| 1 Day | 1 Year | 2 Years | 2x |
| 1 Week | 5 Years | 5 Years | None |
| 1 Month | 20 Years | 20 Years | None |
| 1 Year | 100 Years | 100 Years | None |

As mentioned in the main text, points are now consumed when downloading historical data via API calling if users do not own the sensors from which they are requesting data. Using the “get_sensor_history” endpoint costs 2 points for each request. One data field (e.g., ‘*pm2.5_alt*’) costs 1 point per request/per row if the data is from only channel A or channel B (e.g., ‘*pm1.0_cf_1_a*’, ‘*pm2.5_atm_b*’), and costs 2 points per request/per row if the data is the average of channel A and channel B. Thus, if a user needs one day (24 hours) of *pm2.5_alt* and *pm2.5_cf_1_a* data at 10-minute intervals (i.e., 144 rows), it will consume 434 points ([2 points for “get_sensor_history”] x 1 request + [3 points for “pm2.5_alt” and “pm2.5_cf_1_a” per row] x 144 rows = 434 points). The actual monetary cost of points varies depending on the number of data points and the number of requests required at a time (see **Table S1**). To help understand these costs of data retrieval, **Table S3** summarizes the amount of points needed to download data from just one PA monitor via Wi-Fi calling and the total cost to users for different scenarios that one might encounter in conducting indoor or outdoor field investigations. For simplicity, cost estimates are made using only the assumption of spending $10-49 at a time, meaning 100,000 points per USD (or, alternatively, $0.00001 per point). With this assumption, the costs shown would scale linearly with the number of concurrently deployed monitors, such that downloading one year of all 2-channel-averaged PM data at 10-minute intervals (i.e., the fourth-to-last row in **Table S3**) from 20 deployed monitors in a field campaign would cost $148.

Table S3. Amount of points needed to download PA data from one monitor via Wi-Fi API calling via one request and the total cost to users for different scenarios

| Data Duration | Data Fields Selected | # of Data Fields | Time Interval | Number of Points Needed | Total Cost to User, $ USD* |
| --- | --- | --- | --- | --- | --- |
| 30 days | Data from channel A or B | 1 | 10-min | 4,322 | $ 0.04 |
|  |  |  | 1-hour | 722 | $ 0.01 |
|  | 2-channel-averaged data |  | 10-min | 8,642 | $ 0.09 |
|  |  |  | 1-hour | 1,442 | $ 0.01 |
|  | All number count data - from channel A or B | 6 | 10-min | 25,922 | $ 0.26 |
|  |  |  | 1-hour | 4,322 | $ 0.04 |
|  | All number count data –  2-channel-averaged |  | 10-min | 51,842 | $ 0.52 |
|  |  |  | 1-hour | 8,642 | $ 0.09 |
|  | All PM data - from channel A or B | 7 | 10-min | 30,242 | $ 0.30 |
|  |  |  | 1-hour | 5,042 | $ 0.05 |
|  | All PM data –  2-channel-averaged |  | 10-min | 60,482 | $ 0.60 |
|  |  |  | 1-hour | 10,082 | $ 0.10 |
|  | All data (PM, number count, T, RH, and P) | 48 | 10-min | 276,482 | $ 2.76 |
|  |  |  | 1-hour | 46,082 | $ 0.46 |
| 365 days | Data from channel A or B | 1 | 10-min | 52,562 | $ 0.53 |
|  |  |  | 1-hour | 8,762 | $ 0.09 |
|  | 2-channel-averaged data |  | 10-min | 105,122 | $ 1.05 |
|  |  |  | 1-hour | 17,522 | $ 0.18 |
|  | All number count data - from channel A or B | 6 | 10-min | 315,362 | $ 3.15 |
|  |  |  | 1-hour | 52,562 | $ 0.53 |
|  | All number count data –  2-channel-averaged |  | 10-min | 630,722 | $ 6.31 |
|  |  |  | 1-hour | 105,122 | $ 1.05 |
|  | All PM data - from channel A or B | 7 | 10-min | 367,922 | $ 3.68 |
|  |  |  | 1-hour | 61,322 | $ 0.61 |
|  | All PM data –  2-channel-averaged |  | 10-min | 735,842 | $ 7.36 |
|  |  |  | 1-hour | 122,642 | $ 1.23 |
|  | All data (PM, number count, T, RH, and P) | 48 | 10-min | 3,363,842 | $ 33.64 |
|  |  |  | 1-hour | 560,642 | $ 5.61 |

*All scenarios assume spending $10-49 at a time (100,000 points per USD, or $0.00001/point; Table S1)

To illustrate this pricing structure further, in our 2023 deployments in the FRESSCA field study, we sought to obtain approximately five consecutive months (i.e., 150 days) of 10-minute interval data from a total of 58 PA-II monitors. If we did not purchase the PurpleAir monitors but instead downloaded the data from someone else’s monitors from the PA Map, or had we not obtained free download access via request, we would need a total of 80,179,316 points to obtain 48 data fields from each monitor, given that the time durations were consistent across all homes (i.e. 58 monitors x (2 points + 64 points x (150 days) x 144 rows per day)). If we chose to download all the data at the conclusion of the study at once, the cost per points needed (**Table S1**) would be lower than if we downloaded data routinely throughout the study, and the total cost to download would be $400 (i.e., 200,000 points per USD in bulk). However, waiting until the completion of a field study to download data is not advised, as it does not allow time for interim data collection and inspection for quality assurance and quality control (QA/QC) purposes. Instead, if we decided to download data more frequently (e.g., weekly) and spent only $10-49 at a time, the total download cost would be $802. Our experiences suggest that it is worth spending more to download more frequently, if possible, but other use cases may vary. Our overall recommendation is that developing a thorough data acquisition plan in advance of monitor deployments can help reduce the project budget and ensure a high data collection success rate.

# 3. PurpleAir Co-location Summary

Table S4 summarizes PA-II monitor co-location measurements and data collection across different dates of the study.

Table S4. Summary of PA-II monitor co-location measurements and data collection

| **Dates** | **Total Number of Monitors** | **Monitor type** | | **Download method** | | **Purpose of Co-location** | **Study Period** |
| --- | --- | --- | --- | --- | --- | --- | --- |
|  |  | **Wi-Fi + SD** | **Wi-Fi Only** | **Wi-Fi API Calling** | **SD Card Extrac-tion** |  |  |
| Mar 3, 2023 – Apr 3, 2023 | 35 | 9 | 25 | 32 | 2 | To identify any problematic monitors prior to deployment. Batches were limited to fewer than 40 monitors to keep within maximum number of Wi-Fi connections. | After pilot year deployment |
| Apr 3, 2023 – Apr 17, 2023 | 38 | 38 | 0 | 38 | 0 |  |  |
| May 19, 2023 – Jun 5, 2023 | 5 | 5 | 0 | 5 | 0 |  |  |
| Nov 13, 2023 – Nov 29, 2023 | 57* | 42 | 15 | 49 | 8 | To co-locate all available monitors. Batches were increased in number to accommodate both Wi-Fi and onboard microSD. | After intervention year deployment |
| Dec 21, 2023 – Jan 7, 2024 | 6 | 6 | 0 | 6 | 0 | To co-locate a previously unavailable monitor with five high-performing monitors, repeated for two monitors in need of separate co-location |  |
| Mar 7, 2024 – Mar 18, 2024 | 6 | 6 | 0 | 6 | 0 |  |  |

* We focus on results from this largest co-location measurement campaign from November 2023.

# 4. PurpleAir Co-location Results

**Figure S2** shows linear regression results for *pm2.5_alt* concentrations from a total of 57 monitors that were concurrently deployed in a large research garage for co-location testing after the field measurements were conducted in the intervention year (2023); this is the largest batch of testing as summarized in **Table S4**. Each monitor on the x-axis is labeled as the unique home ID from which it had been deployed in the field (FK = Fresno Kings, KE = Kern County). The arbitrary reference monitor, which had the greatest amount of co-location data captured, is labeled KE22 Outdoor.


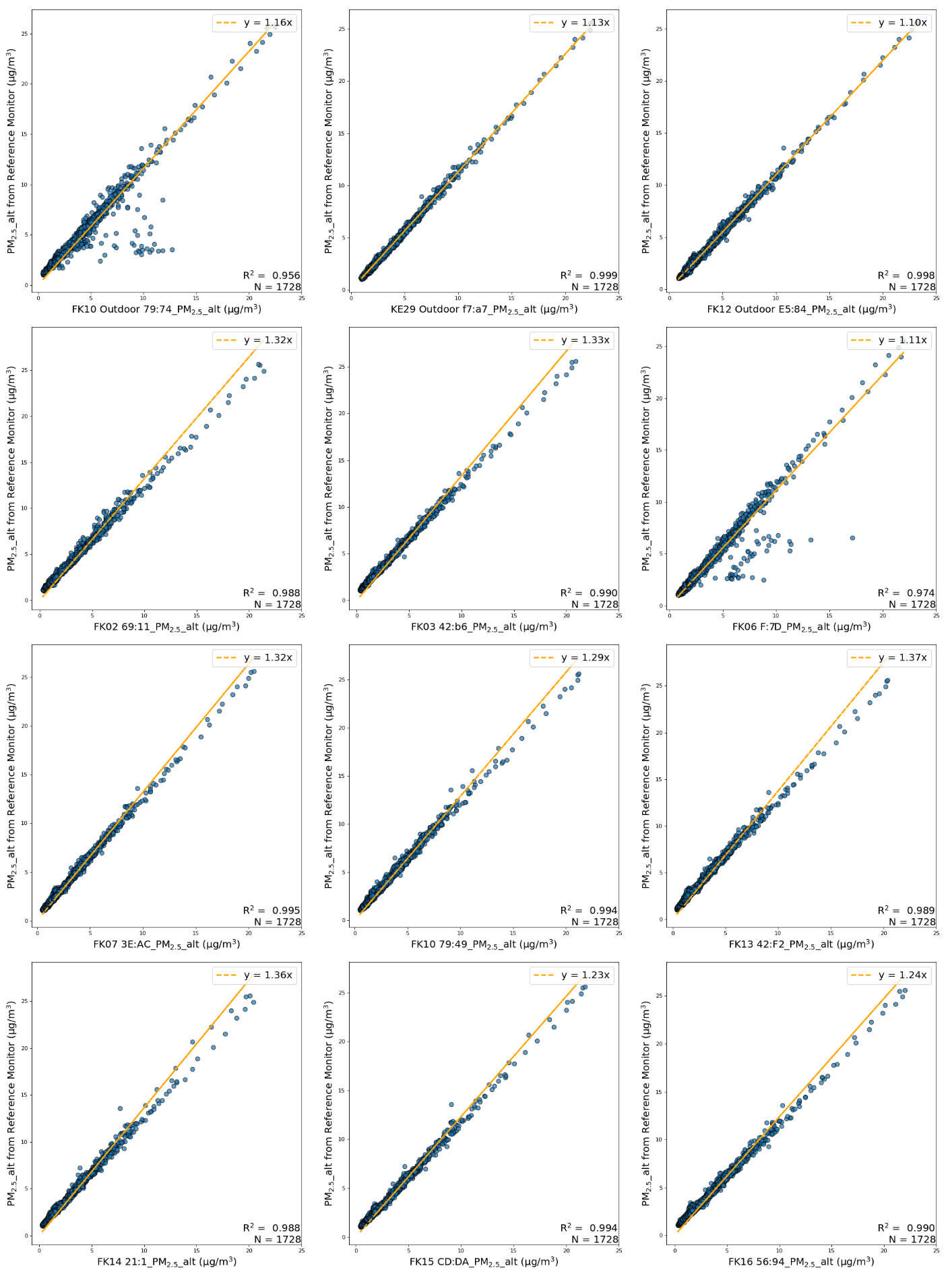


**Figure S2.** Scatter plots and regression results of concurrent *PM_2.5__alt* concentrations from each monitor versus the arbitrary reference monitor from the largest co-location test


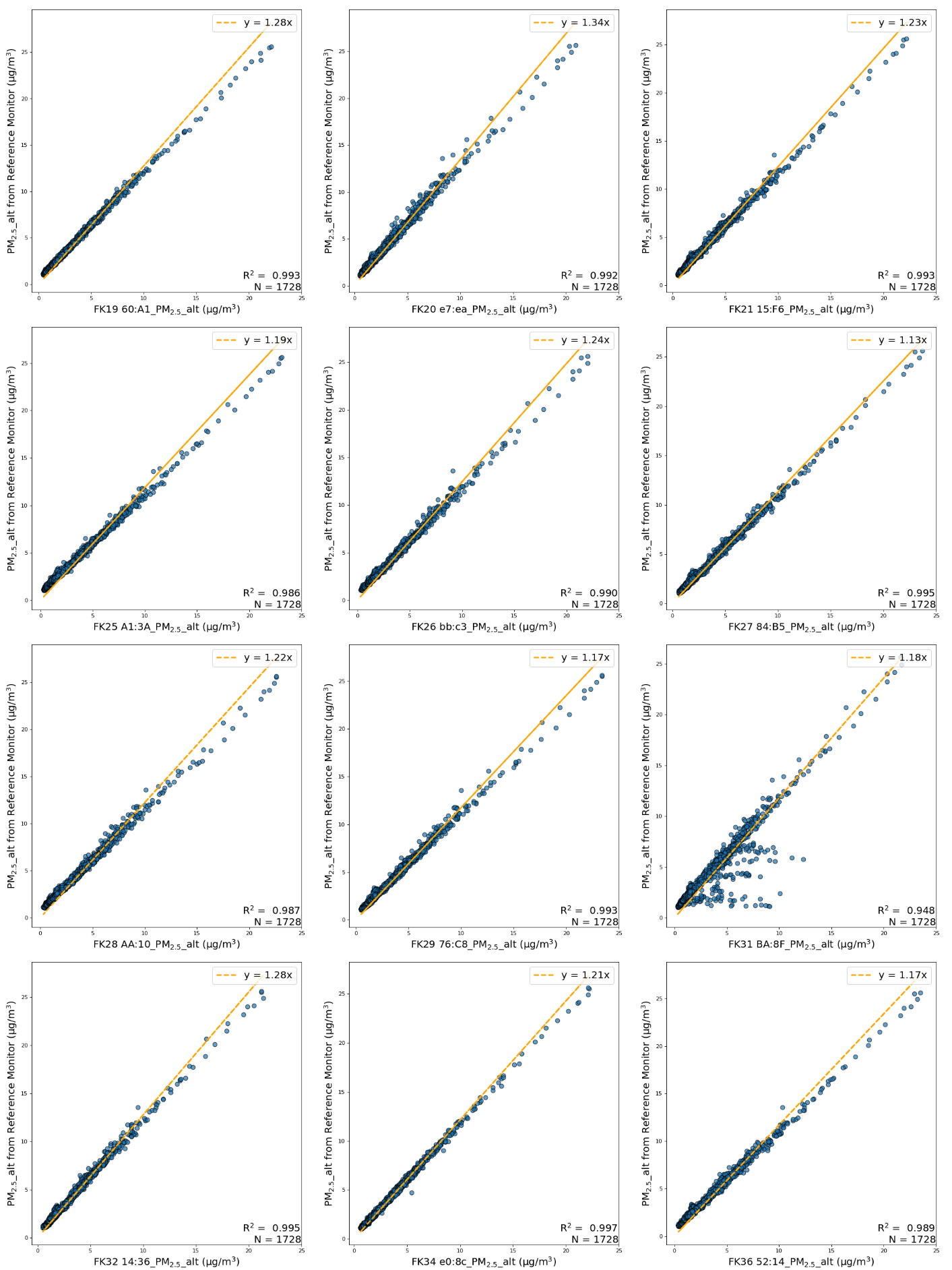


**Figure S2 (continued).** Scatter plots and regression results of concurrent *PM_2.5__alt* concentrations from each monitor versus the arbitrary reference monitor from the largest co-location test


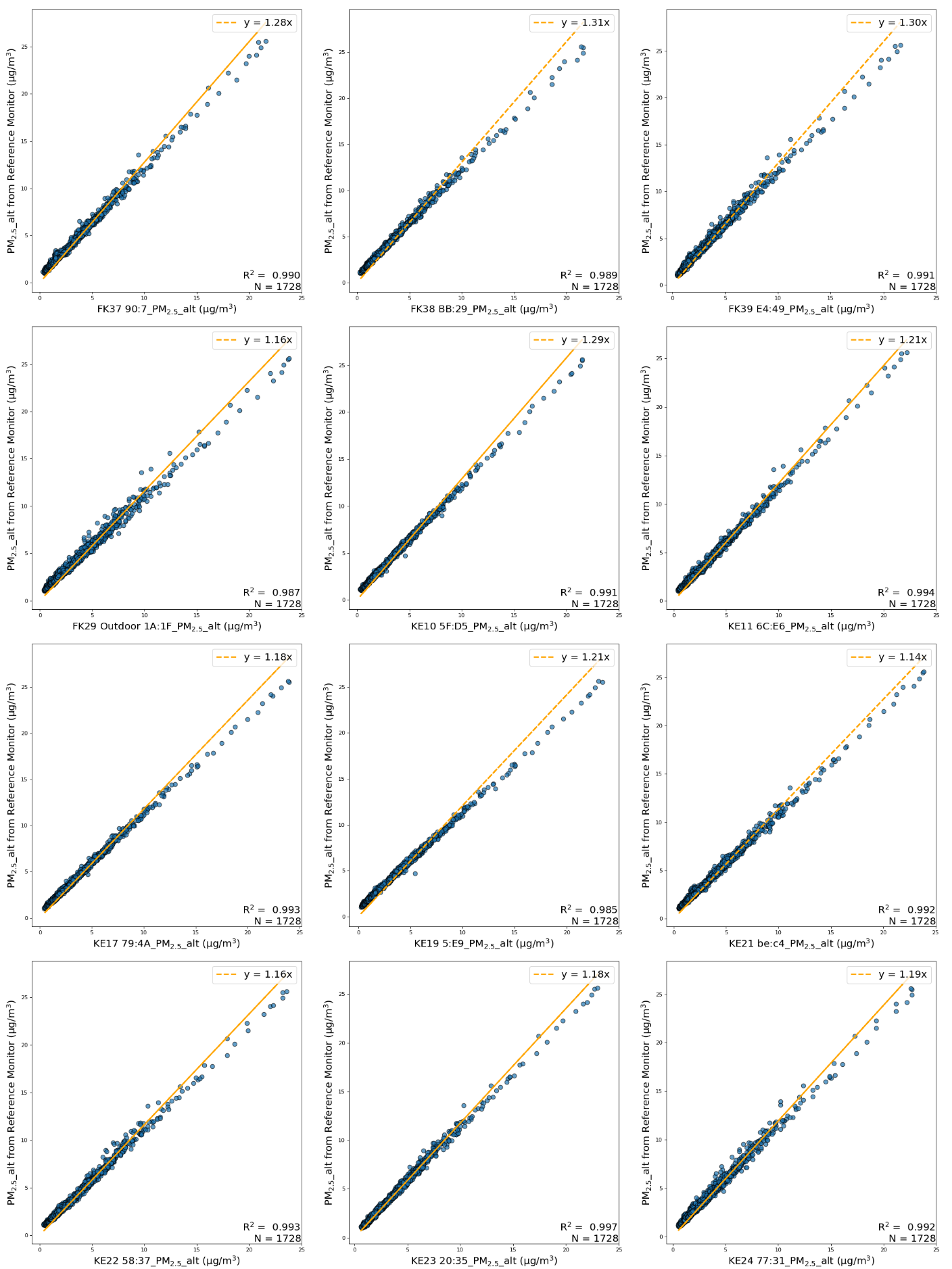


**Figure S2 (continued).** Scatter plots and regression results of concurrent *PM_2.5__alt* concentrations from each monitor versus the arbitrary reference monitor from the largest co-location test


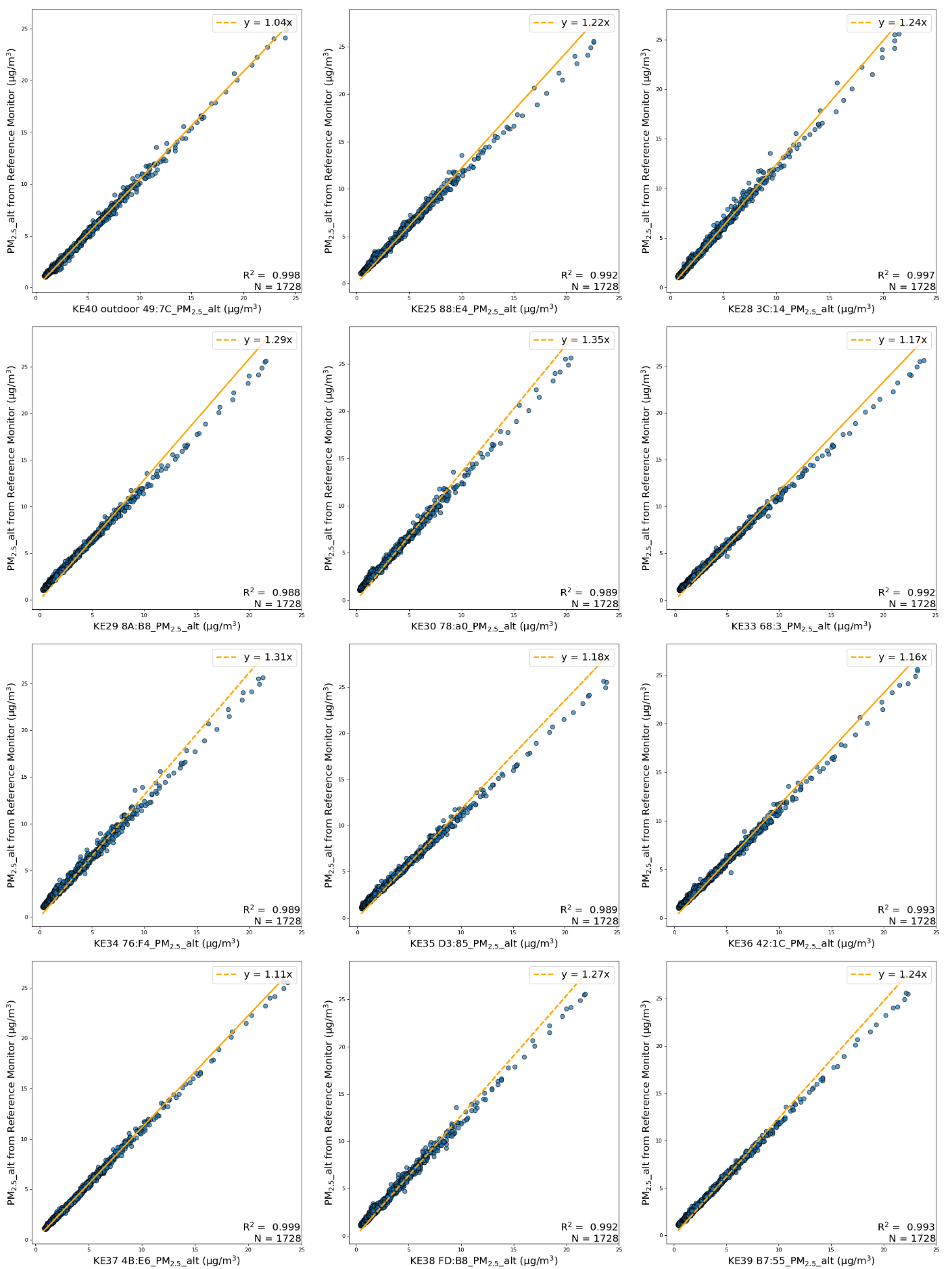


**Figure S2 (continued).** Scatter plots and regression results of concurrent *PM_2.5__alt* concentrations from each monitor versus the arbitrary reference monitor from the largest co-location test


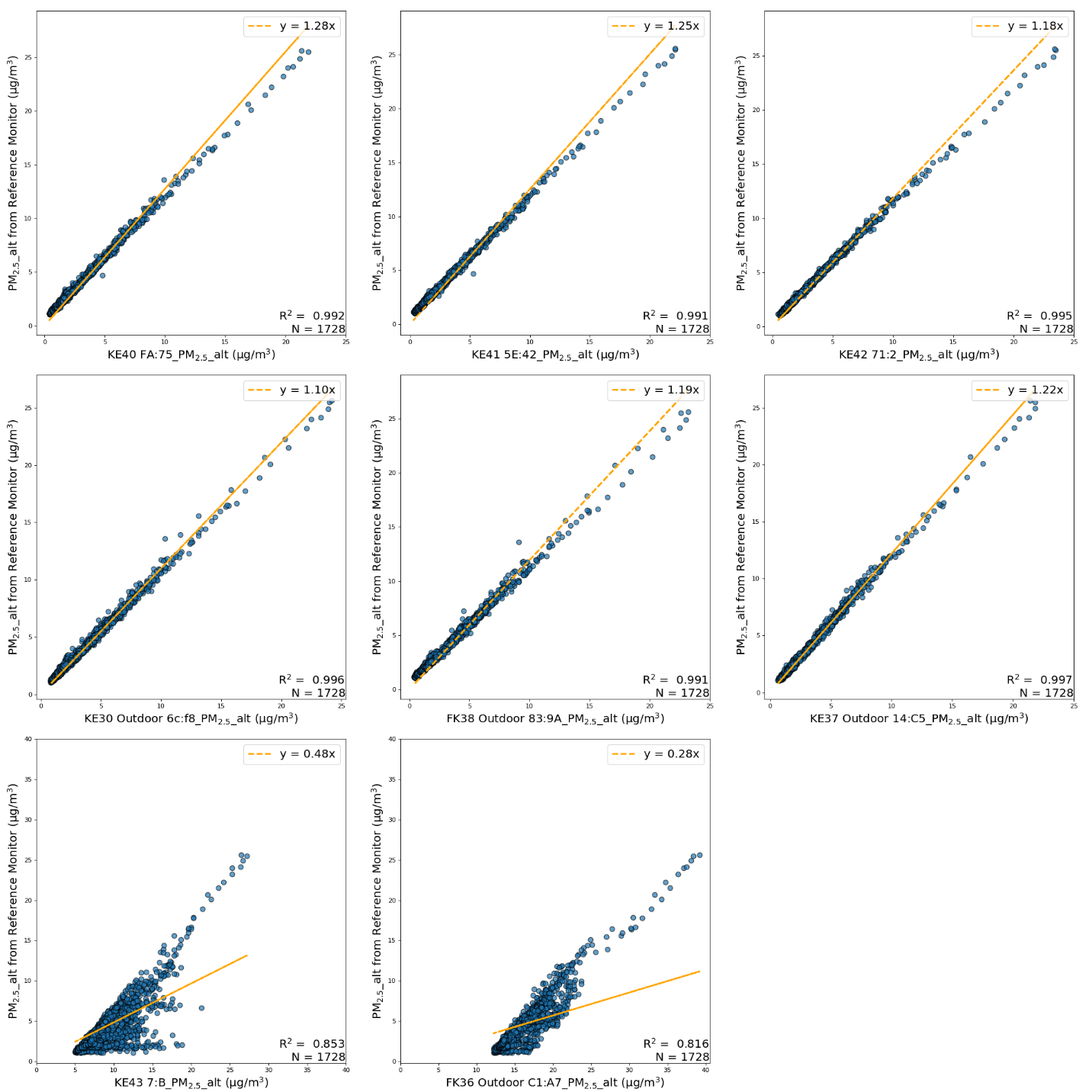


**Figure S2 (continued).** Scatter plots and regression results of concurrent *PM_2.5__alt* concentrations from each monitor versus the arbitrary reference monitor from the largest co-location test

PM_2.5__alt concentrations from 55 of the 57 monitors had a strong linear relationship versus the arbitrary reference monitor, while two monitors clearly had offsets (i.e., nonzero intercepts), showing systematic discrepancy. After visual inspection of the A and B channels of these two potentially problematic monitors, as explained in the main text, we next generated a new reference PM_2.5__alt as the average of the remaining 55 acceptable monitors. **Figure S3** shows linear regression results for PM_2.5__alt concentrations from all 57 monitors versus the average of the 55 acceptable monitors.


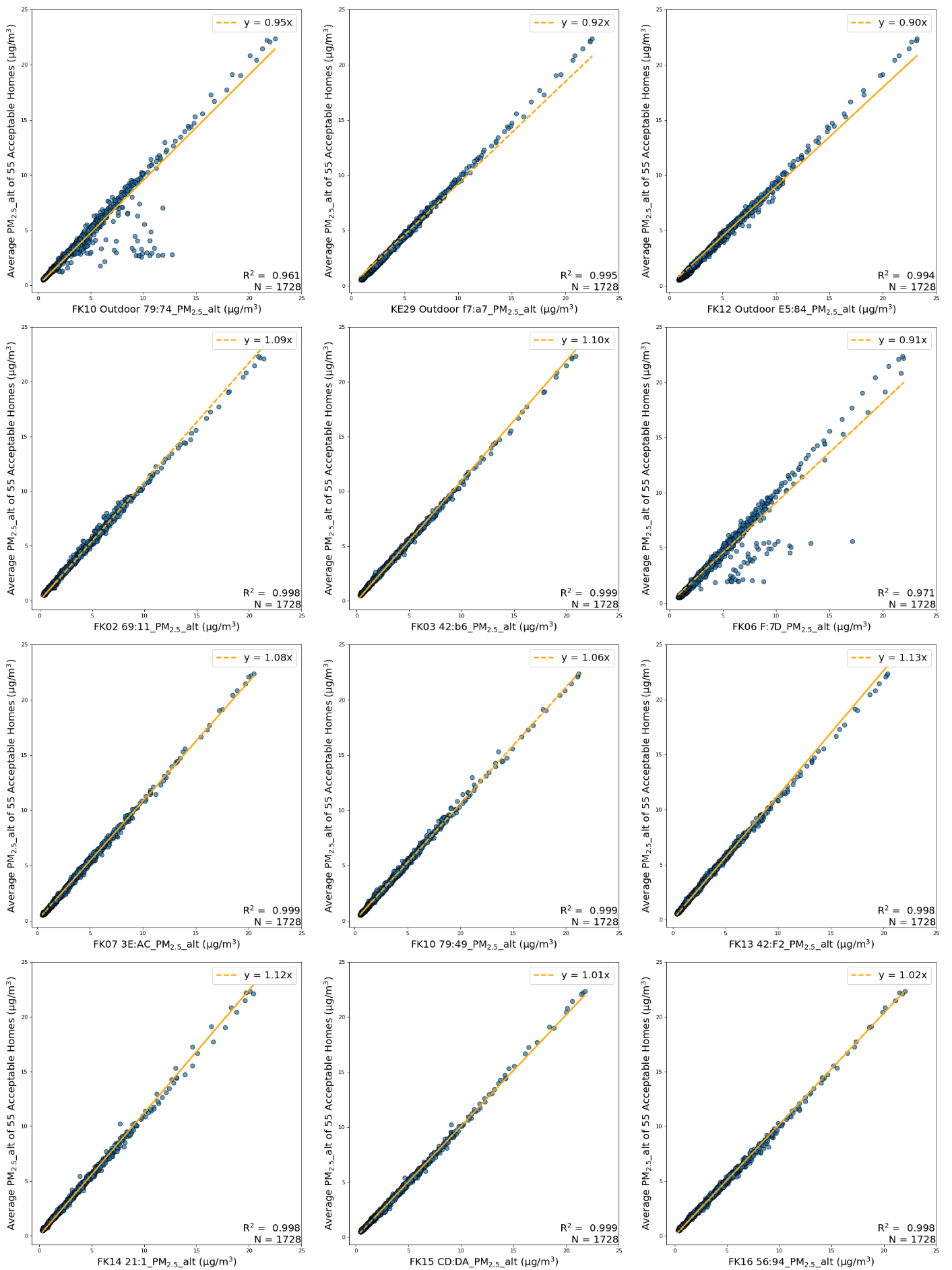


**Figure S3.** Scatter plots and regression results of concurrent PM2.5_alt concentrations from each monitor versus the average of 55 acceptable monitors from the largest co-location test

**
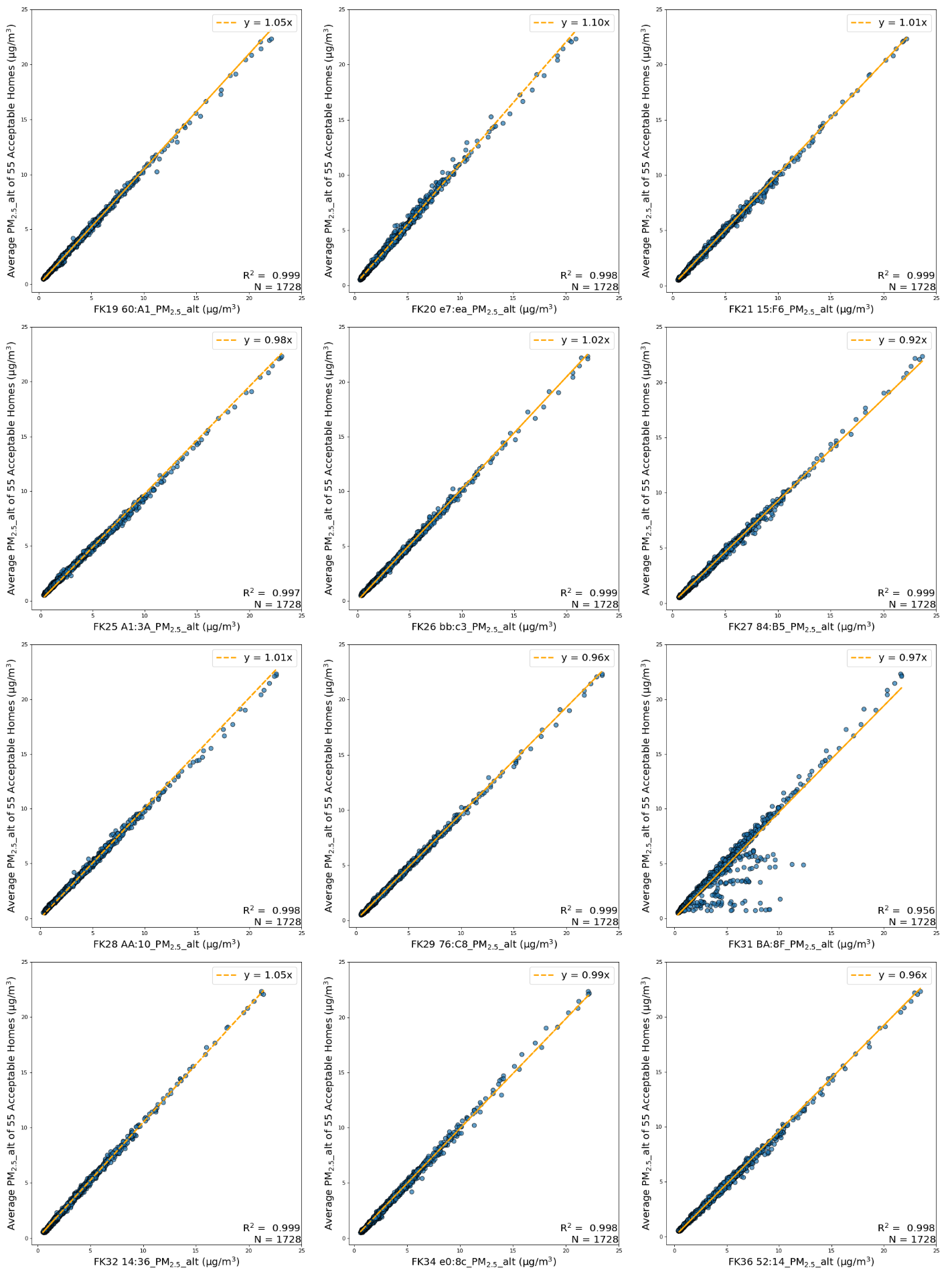
**

**Figure S3 (continued).** Scatter plots and regression results of concurrent *PM_2.5__alt* concentrations from each monitor versus the average of 55 acceptable monitors from the largest co-location test


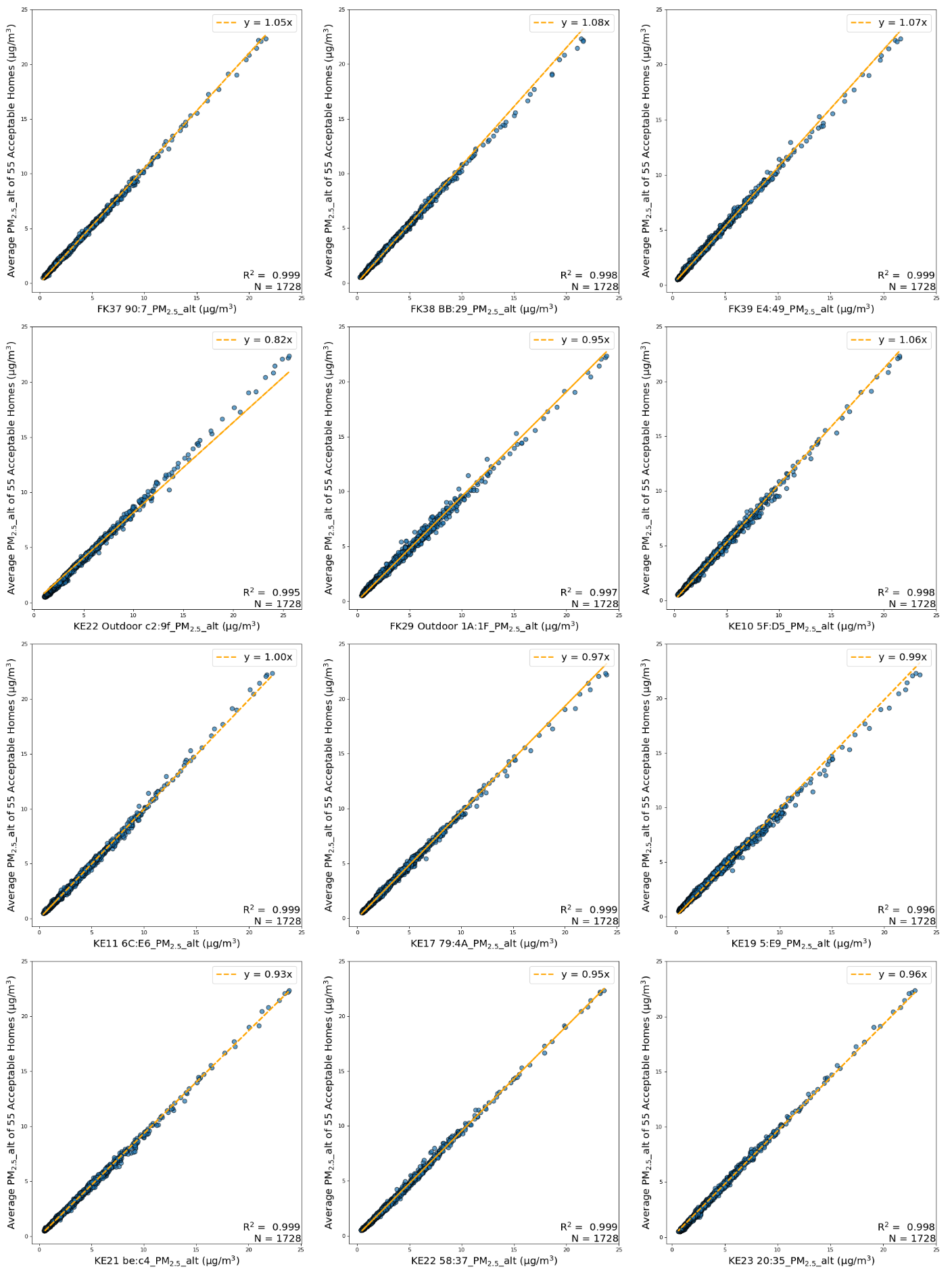


**Figure S3 (continued).** Scatter plots and regression results of concurrent *PM_2.5__alt* concentrations from each monitor versus the average of 55 acceptable monitors from the largest co-location test


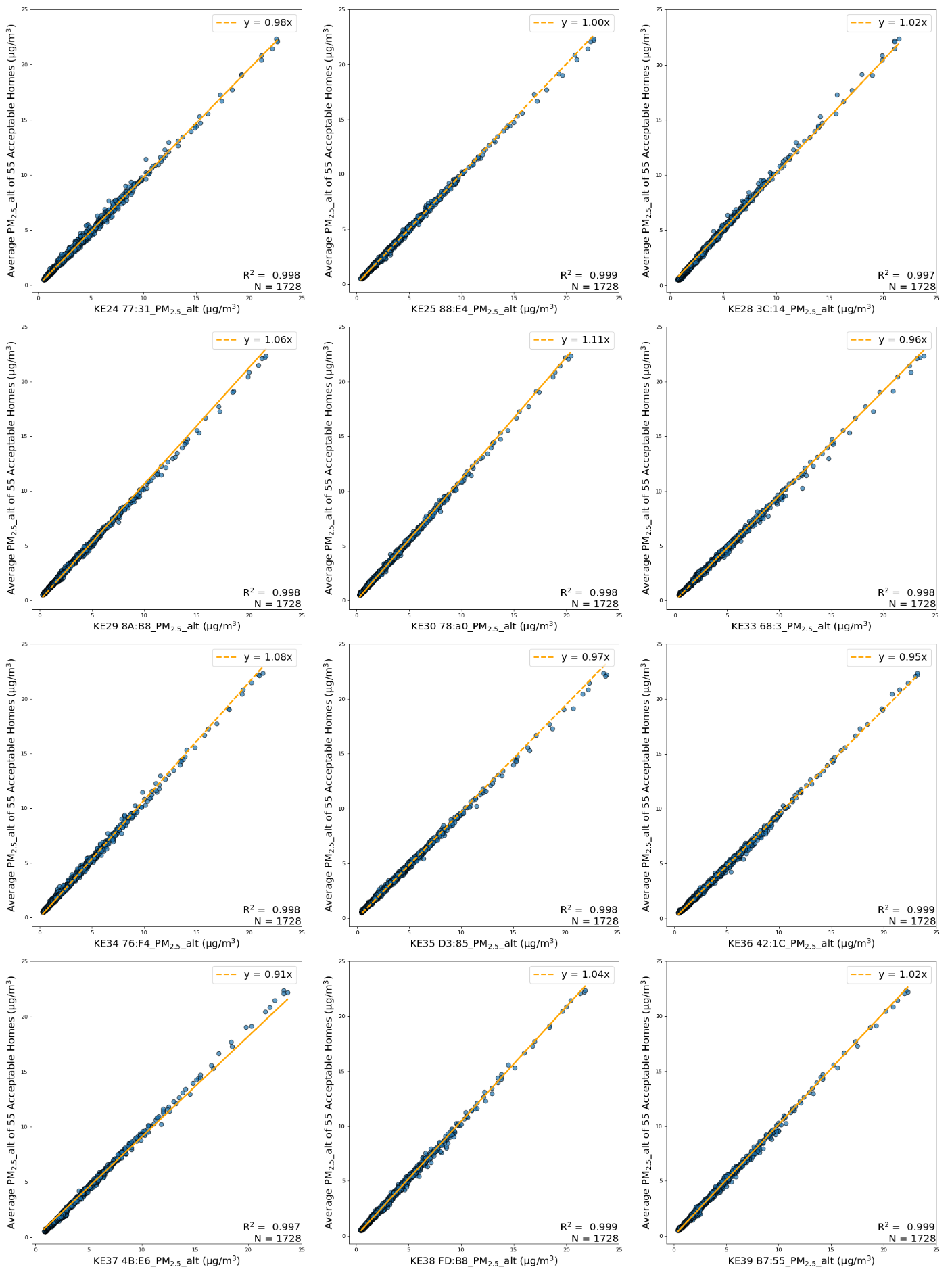


**Figure S3 (continued).** Scatter plots and regression results of concurrent PM2.5_alt concentrations from each monitor versus the average of 55 acceptable monitors from the largest co-location test


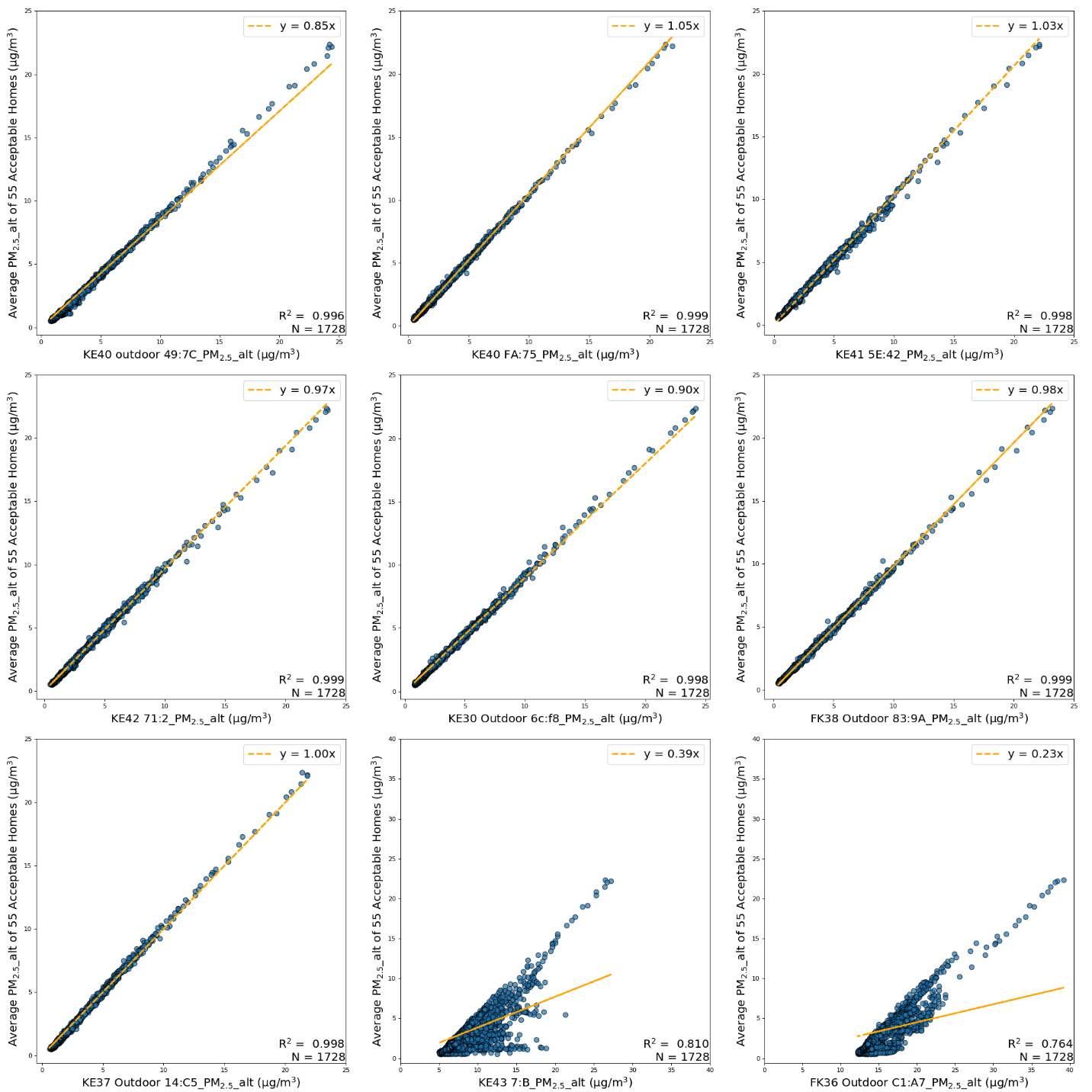


**Figure S3 (continued).** Scatter plots and regression results of concurrent PM2.5_alt concentrations from each monitor versus the average of 55 acceptable monitors from the largest co-location test

**Figure S4** shows a time-series profile of the average PM_2.5__alt concentrations from 55 acceptable monitors during the primary garage co-location measurements to explore the nature of PM sources. The trends show that while the bulk of indoor PM likely infiltrated from outdoors, with slow increasing and decreases over time, there were a few clear patterns of intermittent sources followed by a classic exponential (first order) decay, which might be attributable to indoor sources and/or short-term garage door opening during periods of elevated outdoor concentrations. However, as we did not have concurrent outdoor measurements, it is difficult to identify the sources. Nevertheless, the garage appeared to include a mix of ambient-infiltrated PM and intermittent, yet uncharacterized, indoor PM sources.


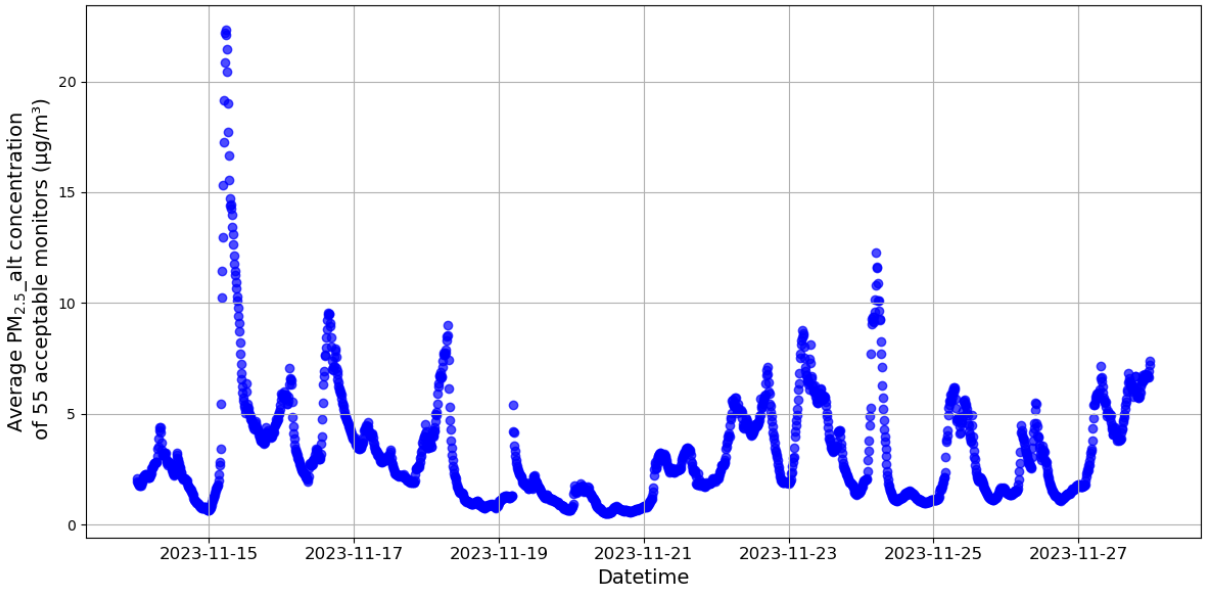


Figure S4. Time-series profile of the average PM_2.5__alt concentrations from 55 acceptable monitors during the primary garage co-location measurements

**Figure S5** shows an example of PM_2.5__alt concentrations resulting from one of the poorer performing monitors against the arbitrary reference, including the average of A and B channels and channels A and B separately to investigate whether one of the channels was problematic. **Figure S5a** indicates that the regression between monitor C1:A7’s *pm2.5_alt* average channel and the reference has an offset (intercept), while **Figure S5b** and **Figure S5c** also show a similar offset for the individual channel responses. Since both channel A and B show similar regression results, with R^2^ similar to the mean of ~0.8, it appears that an intercept could be added to the regression to improve performance and maintain usability. In this case, an intercept was added to the linear regression equation as shown in **Figure S6**.


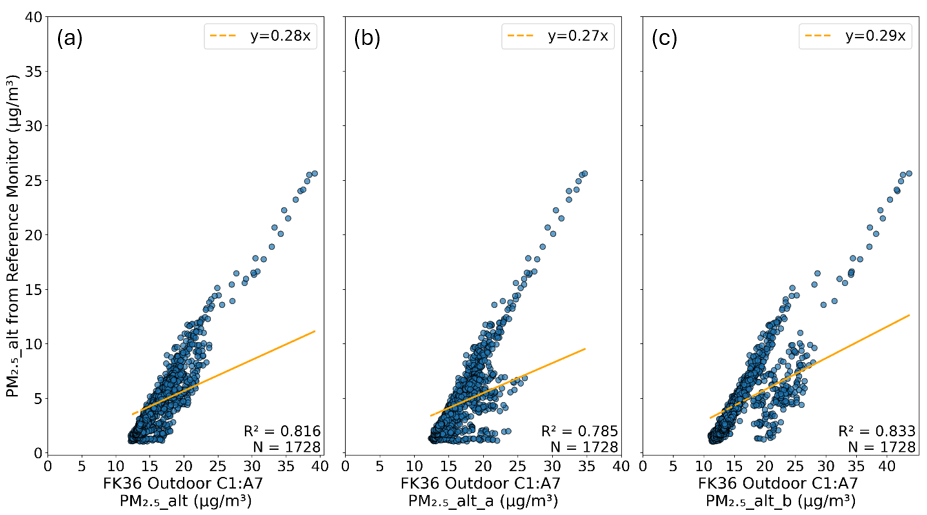


Figure S5. Example regression of monitor FK36 (C1:A7) *pm2.5_alt* concentrations against an arbitrary reference: (a) average of channel A and B; (b) channel A only; and (c) channel B only.


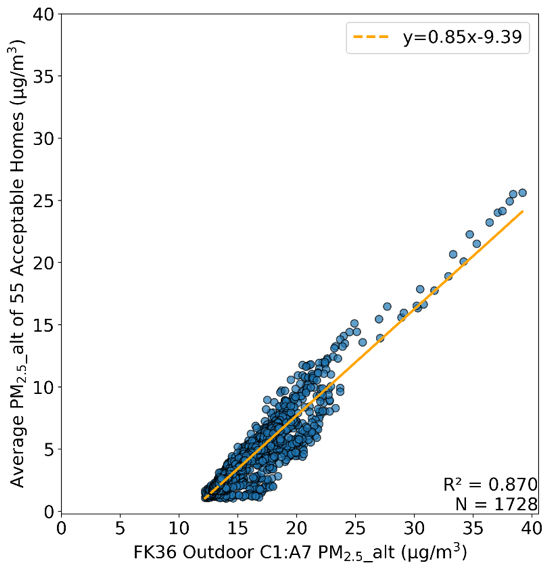


**Figure S6**. Example regression of monitor C1:A7’s *pm2.5_alt*: adding an intercept to the regression

With the co-location reference set as the average of 55 acceptable monitors, the co-location results of all PM related parameters, as well as temperature, humidity and pressure (all using the average of channel A and B) are summarized in **Table S5**. Zero intercept was set for all PM related parameters.

Table S5. November 2023 co-location regression results: each monitor (excluding two unacceptable monitors) versus the mean of 55 acceptable monitors

| **Monitor Registered Name** | **pm2.5_alt** | | **pm1.0_cf_1** | | **pm2.5_cf_1** | | **pm10.0_cf_1** | | **pm1.0_atm** | | **pm2.5_atm** | | **pm10.0_atm** | | **0.3_µm_count** | |
| --- | --- | --- | --- | --- | --- | --- | --- | --- | --- | --- | --- | --- | --- | --- | --- | --- |
|  | **Slope** | **R^2^** | **Slope** | **R^2^** | **Slope** | **R^2^** | **Slope** | **R^2^** | **Slope** | **R^2^** | **Slope** | **R^2^** | **Slope** | **R^2^** | **Slope** | **R^2^** |
| FK02 | 1.09 | 1.00 | 1.16 | 1.00 | 1.08 | 1.00 | 1.05 | 1.00 | 1.15 | 0.99 | 1.08 | 1.00 | 1.05 | 1.00 | 1.14 | 0.99 |
| FK03 | 1.10 | 1.00 | 1.11 | 1.00 | 1.07 | 1.00 | 1.07 | 1.00 | 1.10 | 1.00 | 1.07 | 1.00 | 1.07 | 1.00 | 1.14 | 1.00 |
| FK06 | 0.91 | 0.97 | 2.00 | 0.95 | 0.92 | 0.97 | 0.86 | 0.97 | 0.92 | 0.96 | 0.92 | 0.98 | 0.86 | 0.98 | 0.75 | 0.83 |
| FK07 | 1.08 | 1.00 | 1.12 | 1.00 | 1.08 | 1.00 | 1.07 | 1.00 | 1.12 | 1.00 | 1.07 | 1.00 | 1.06 | 1.00 | 1.03 | 1.00 |
| FK10 | 1.06 | 1.00 | 1.09 | 1.00 | 1.04 | 1.00 | 1.03 | 1.00 | 1.09 | 1.00 | 1.04 | 1.00 | 1.03 | 1.00 | 1.14 | 1.00 |
| FK13 | 1.13 | 1.00 | 1.20 | 1.00 | 1.17 | 1.00 | 1.15 | 1.00 | 1.19 | 1.00 | 1.16 | 1.00 | 1.15 | 1.00 | 1.19 | 1.00 |
| FK14 | 1.12 | 1.00 | 1.09 | 1.00 | 1.12 | 1.00 | 1.16 | 1.00 | 1.08 | 1.00 | 1.12 | 1.00 | 1.15 | 1.00 | 1.10 | 1.00 |
| FK15 | 1.01 | 1.00 | 1.05 | 1.00 | 0.99 | 1.00 | 0.95 | 1.00 | 1.05 | 1.00 | 0.99 | 1.00 | 0.95 | 1.00 | 1.07 | 1.00 |
| FK16 | 1.02 | 1.00 | 1.03 | 1.00 | 1.02 | 1.00 | 0.98 | 1.00 | 1.03 | 1.00 | 1.02 | 1.00 | 0.98 | 1.00 | 1.06 | 1.00 |
| FK19 | 1.05 | 1.00 | 1.11 | 1.00 | 1.06 | 1.00 | 1.08 | 1.00 | 1.11 | 1.00 | 1.06 | 1.00 | 1.08 | 1.00 | 1.13 | 1.00 |
| FK20 | 1.10 | 1.00 | 1.16 | 1.00 | 1.13 | 1.00 | 1.14 | 1.00 | 1.16 | 1.00 | 1.12 | 1.00 | 1.14 | 1.00 | 1.18 | 1.00 |
| FK21 | 1.01 | 1.00 | 1.04 | 1.00 | 1.03 | 1.00 | 1.08 | 1.00 | 1.03 | 1.00 | 1.03 | 1.00 | 1.08 | 1.00 | 1.06 | 1.00 |
| FK25 | 0.98 | 1.00 | 0.89 | 0.94 | 0.98 | 0.99 | 1.02 | 0.99 | 0.96 | 1.00 | 0.99 | 1.00 | 1.03 | 1.00 | 1.07 | 1.00 |
| FK26 | 1.02 | 1.00 | 1.02 | 1.00 | 1.03 | 1.00 | 1.06 | 1.00 | 1.02 | 1.00 | 1.02 | 1.00 | 1.05 | 1.00 | 1.07 | 1.00 |
| FK27 | 0.92 | 1.00 | 0.96 | 1.00 | 0.92 | 1.00 | 0.91 | 1.00 | 0.96 | 1.00 | 0.92 | 1.00 | 0.91 | 1.00 | 1.00 | 1.00 |
| FK28 | 1.01 | 1.00 | 1.01 | 1.00 | 1.02 | 1.00 | 1.04 | 1.00 | 1.01 | 1.00 | 1.02 | 1.00 | 1.03 | 1.00 | 1.00 | 1.00 |
| FK29 | 0.96 | 1.00 | 0.91 | 1.00 | 0.95 | 1.00 | 0.94 | 1.00 | 0.91 | 1.00 | 0.95 | 1.00 | 0.94 | 1.00 | 1.00 | 1.00 |
| FK31 | 0.97 | 0.96 | 0.88 | 0.91 | 0.97 | 0.95 | 0.99 | 0.96 | 0.90 | 0.92 | 0.97 | 0.95 | 0.99 | 0.96 | 0.76 | 0.79 |
| FK32 | 1.05 | 1.00 | 0.98 | 1.00 | 1.00 | 1.00 | 1.01 | 1.00 | 0.98 | 1.00 | 1.00 | 1.00 | 1.01 | 1.00 | 1.08 | 1.00 |
| FK34 | 0.99 | 1.00 | 0.98 | 1.00 | 0.97 | 1.00 | 0.94 | 1.00 | 0.98 | 1.00 | 0.97 | 1.00 | 0.94 | 1.00 | 1.02 | 1.00 |
| FK36 | 0.96 | 1.00 | 0.96 | 1.00 | 0.95 | 1.00 | 0.95 | 1.00 | 0.96 | 1.00 | 0.95 | 1.00 | 0.95 | 1.00 | 1.01 | 1.00 |
| FK37 | 1.05 | 1.00 | 1.01 | 1.00 | 1.05 | 1.00 | 1.01 | 1.00 | 1.00 | 1.00 | 1.04 | 1.00 | 1.01 | 1.00 | 1.00 | 1.00 |
| FK38 | 1.08 | 1.00 | 1.08 | 1.00 | 1.05 | 1.00 | 1.08 | 1.00 | 1.08 | 1.00 | 1.06 | 1.00 | 1.08 | 1.00 | 1.10 | 1.00 |
| FK39 | 1.07 | 1.00 | 1.03 | 1.00 | 1.05 | 1.00 | 1.08 | 1.00 | 1.03 | 1.00 | 1.05 | 1.00 | 1.08 | 1.00 | 1.08 | 1.00 |
| KE10 | 1.06 | 1.00 | 1.09 | 1.00 | 1.05 | 1.00 | 1.06 | 1.00 | 1.09 | 1.00 | 1.05 | 1.00 | 1.06 | 1.00 | 1.15 | 1.00 |
| KE11 | 1.00 | 1.00 | 1.03 | 1.00 | 0.96 | 1.00 | 0.97 | 1.00 | 1.03 | 1.00 | 0.96 | 1.00 | 0.97 | 1.00 | 1.07 | 1.00 |
| KE17 | 0.97 | 1.00 | 0.95 | 1.00 | 0.94 | 1.00 | 0.95 | 1.00 | 0.96 | 1.00 | 0.94 | 1.00 | 0.95 | 1.00 | 1.02 | 1.00 |
| KE19 | 0.99 | 1.00 | 0.96 | 1.00 | 1.00 | 1.00 | 1.04 | 1.00 | 0.96 | 1.00 | 1.00 | 1.00 | 1.04 | 1.00 | 1.02 | 0.99 |
| KE21 | 0.93 | 1.00 | 0.92 | 1.00 | 0.92 | 1.00 | 0.95 | 1.00 | 0.93 | 1.00 | 0.92 | 1.00 | 0.95 | 1.00 | 0.99 | 1.00 |
| KE22 | 0.95 | 1.00 | 1.01 | 1.00 | 0.96 | 1.00 | 0.97 | 1.00 | 1.01 | 1.00 | 0.97 | 1.00 | 0.97 | 1.00 | 1.06 | 1.00 |
| KE23 | 0.96 | 1.00 | 0.93 | 1.00 | 0.98 | 1.00 | 0.98 | 1.00 | 0.93 | 1.00 | 0.97 | 1.00 | 0.98 | 1.00 | 0.93 | 1.00 |

Table S5 (continued). November 2023 co-location regression results: each monitor (excluding two unacceptable monitors) versus the mean of 55 acceptable monitors

| **Monitor Registered Name** | **pm2.5_alt** | | **pm1.0_cf_1** | | **pm2.5_cf_1** | | **pm10.0_cf_1** | | **pm1.0_atm** | | **pm2.5_atm** | | **pm10.0_atm** | | **0.3_µm_count** | |
| --- | --- | --- | --- | --- | --- | --- | --- | --- | --- | --- | --- | --- | --- | --- | --- | --- |
|  | **Slope** | **R^2^** | **Slope** | **R^2^** | **Slope** | **R^2^** | **Slope** | **R^2^** | **Slope** | **R^2^** | **Slope** | **R^2^** | **Slope** | **R^2^** | **Slope** | **R^2^** |
| KE24 | 0.98 | 1.00 | 0.99 | 1.00 | 0.98 | 1.00 | 0.99 | 1.00 | 0.99 | 1.00 | 0.98 | 1.00 | 0.99 | 1.00 | 1.01 | 1.00 |
| KE25 | 1.00 | 1.00 | 1.00 | 1.00 | 0.98 | 1.00 | 0.97 | 1.00 | 1.01 | 1.00 | 0.98 | 1.00 | 0.97 | 1.00 | 1.05 | 1.00 |
| KE28 | 1.02 | 1.00 | 0.92 | 0.98 | 0.95 | 0.99 | 0.97 | 0.99 | 0.92 | 0.98 | 0.95 | 0.99 | 0.97 | 0.99 | 0.96 | 0.99 |
| KE29 | 1.06 | 1.00 | 1.04 | 1.00 | 1.04 | 1.00 | 1.08 | 1.00 | 1.04 | 1.00 | 1.04 | 1.00 | 1.08 | 1.00 | 1.08 | 1.00 |
| KE30 | 1.11 | 1.00 | 1.16 | 1.00 | 1.14 | 1.00 | 1.15 | 1.00 | 1.15 | 1.00 | 1.13 | 1.00 | 1.15 | 1.00 | 1.16 | 1.00 |
| KE33 | 0.96 | 1.00 | 0.97 | 1.00 | 0.96 | 1.00 | 0.98 | 1.00 | 0.97 | 1.00 | 0.96 | 1.00 | 0.98 | 1.00 | 0.99 | 1.00 |
| KE34 | 1.08 | 1.00 | 1.08 | 1.00 | 1.06 | 1.00 | 1.06 | 1.00 | 1.08 | 1.00 | 1.06 | 1.00 | 1.06 | 1.00 | 1.11 | 1.00 |
| KE35 | 0.97 | 1.00 | 0.98 | 1.00 | 0.98 | 1.00 | 1.00 | 1.00 | 0.98 | 1.00 | 0.98 | 1.00 | 1.00 | 1.00 | 1.01 | 1.00 |
| KE36 | 0.95 | 1.00 | 0.99 | 1.00 | 0.94 | 1.00 | 0.96 | 1.00 | 0.99 | 1.00 | 0.94 | 1.00 | 0.96 | 1.00 | 1.03 | 1.00 |
| KE37 | 0.91 | 1.00 | 0.84 | 0.99 | 0.91 | 0.99 | 0.86 | 1.00 | 0.84 | 0.99 | 0.91 | 0.99 | 0.87 | 1.00 | 0.83 | 0.99 |
| KE38 | 1.04 | 1.00 | 1.09 | 1.00 | 1.06 | 1.00 | 1.08 | 1.00 | 1.09 | 1.00 | 1.06 | 1.00 | 1.08 | 1.00 | 1.08 | 1.00 |
| KE39 | 1.02 | 1.00 | 1.03 | 1.00 | 1.00 | 1.00 | 1.03 | 1.00 | 1.03 | 1.00 | 1.00 | 1.00 | 1.03 | 1.00 | 1.09 | 1.00 |
| KE40 | 1.05 | 1.00 | 1.11 | 1.00 | 1.05 | 1.00 | 1.04 | 1.00 | 1.11 | 1.00 | 1.05 | 1.00 | 1.03 | 1.00 | 1.14 | 1.00 |
| KE41 | 1.03 | 1.00 | 1.08 | 1.00 | 1.04 | 1.00 | 1.07 | 1.00 | 1.08 | 1.00 | 1.04 | 1.00 | 1.07 | 1.00 | 1.10 | 0.99 |
| KE42 | 0.97 | 1.00 | 1.00 | 1.00 | 0.95 | 1.00 | 0.96 | 1.00 | 1.00 | 1.00 | 0.95 | 1.00 | 0.96 | 1.00 | 1.02 | 1.00 |
| FK10 Outdoor | 0.95 | 0.96 | 0.90 | 0.91 | 0.97 | 0.96 | 0.98 | 0.96 | 0.94 | 0.95 | 0.99 | 0.97 | 0.98 | 0.96 | 0.75 | 0.82 |
| KE29 Outdoor | 0.92 | 0.99 | 0.89 | 0.99 | 0.95 | 0.99 | 0.88 | 0.99 | 0.89 | 0.99 | 0.95 | 0.99 | 0.88 | 0.99 | 0.83 | 0.99 |
| FK12 Outdoor | 0.90 | 0.99 | 0.91 | 1.00 | 0.96 | 1.00 | 0.92 | 1.00 | 0.92 | 1.00 | 0.96 | 1.00 | 0.92 | 1.00 | 0.65 | 0.96 |
| KE22 Outdoor | 0.82 | 0.99 | 0.78 | 0.99 | 0.85 | 1.00 | 0.88 | 1.00 | 0.78 | 0.99 | 0.86 | 1.00 | 0.88 | 1.00 | 0.60 | 0.96 |
| FK29 Outdoor | 0.95 | 1.00 | 0.87 | 1.00 | 0.97 | 1.00 | 1.01 | 1.00 | 0.87 | 1.00 | 0.97 | 1.00 | 1.01 | 1.00 | 0.89 | 1.00 |
| KE40 Outdoor | 0.85 | 1.00 | 0.82 | 1.00 | 0.89 | 1.00 | 0.85 | 1.00 | 0.83 | 1.00 | 0.89 | 1.00 | 0.86 | 1.00 | 0.73 | 0.98 |
| KE30 Outdoor | 0.90 | 1.00 | 0.93 | 1.00 | 0.94 | 1.00 | 0.94 | 1.00 | 0.93 | 1.00 | 0.95 | 1.00 | 0.94 | 1.00 | 0.74 | 0.98 |
| FK38 Outdoor | 0.98 | 1.00 | 0.97 | 1.00 | 0.99 | 1.00 | 0.94 | 1.00 | 0.97 | 1.00 | 0.99 | 1.00 | 0.94 | 1.00 | 0.97 | 1.00 |
| KE37 Outdoor | 1.00 | 1.00 | 0.94 | 0.99 | 0.97 | 0.99 | 0.89 | 0.99 | 0.94 | 0.99 | 0.96 | 0.99 | 0.89 | 0.99 | 0.94 | 1.00 |

Table S5 (continued). November 2023 co-location regression results: each monitor (excluding two unacceptable monitors) versus the mean of 55 acceptable monitors

| **Monitor Registered Name** | **0.5_µm_count** | | **1.0_µm_count** | | **2.5_µm_count** | | **5.0_µm_count** | | **10.0_µm_count** | | **temperature** | | **humidity** | | **pressure** | |
| --- | --- | --- | --- | --- | --- | --- | --- | --- | --- | --- | --- | --- | --- | --- | --- | --- |
|  | **Slope** | **R^2^** | **Slope** | **R^2^** | **Slope** | **R^2^** | **Slope** | **R^2^** | **Slope** | **R^2^** | **Slope** | **R^2^** | **Slope** | **R^2^** | **Slope** | **R^2^** |
| FK02 | 1.11 | 1.00 | 1.02 | 1.00 | 0.81 | 0.99 | 0.93 | 0.94 | 0.69 | 0.92 | 0.94 | 0.99 | 1.04 | 1.00 | 0.99 | 1.00 |
| FK03 | 1.10 | 1.00 | 1.06 | 1.00 | 0.92 | 0.99 | 1.04 | 0.94 | 0.87 | 0.92 | 0.86 | 0.99 | 1.02 | 1.00 | 0.99 | 1.00 |
| FK06 | 0.86 | 0.95 | 0.92 | 1.00 | 0.68 | 0.98 | 0.50 | 0.94 | 0.82 | 0.87 | 1.08 | 0.99 | 1.03 | 1.00 | 0.99 | 1.00 |
| FK07 | 1.11 | 1.00 | 1.06 | 1.00 | 0.90 | 0.99 | 0.92 | 0.95 | 0.69 | 0.93 | 1.04 | 0.98 | 1.03 | 1.00 | 0.99 | 1.00 |
| FK10 | 1.08 | 1.00 | 1.00 | 1.00 | 0.86 | 0.99 | 0.88 | 0.96 | 0.64 | 0.95 | 0.99 | 0.99 | 1.04 | 1.00 | 0.99 | 1.00 |
| FK13 | 1.16 | 1.00 | 1.08 | 1.00 | 1.00 | 0.99 | 1.36 | 0.92 | 1.00 | 0.90 | 0.98 | 0.99 | 1.09 | 1.00 | 0.99 | 1.00 |
| FK14 | 1.08 | 1.00 | 1.17 | 1.00 | 1.33 | 0.99 | 1.80 | 0.95 | 1.08 | 0.94 | 1.05 | 0.99 | 0.98 | 1.00 | 0.99 | 1.00 |
| FK15 | 1.03 | 1.00 | 0.94 | 1.00 | 0.69 | 0.99 | 0.77 | 0.92 | 0.77 | 0.89 | 1.03 | 0.96 | 1.02 | 1.00 | 0.98 | 1.00 |
| FK16 | 1.02 | 1.00 | 0.99 | 1.00 | 0.91 | 0.99 | 0.55 | 0.95 | 1.39 | 0.86 | 1.02 | 0.98 | 1.03 | 1.00 | 0.99 | 1.00 |
| FK19 | 1.10 | 1.00 | 1.00 | 1.00 | 1.03 | 0.99 | 1.22 | 0.95 | 1.33 | 0.90 | 1.02 | 0.99 | 1.00 | 1.00 | 0.99 | 1.00 |
| FK20 | 1.13 | 1.00 | 1.07 | 1.00 | 1.18 | 0.98 | 1.28 | 0.93 | 0.90 | 0.91 | 1.08 | 0.98 | 1.27 | 1.00 | 0.99 | 1.00 |
| FK21 | 1.04 | 1.00 | 1.01 | 1.00 | 1.32 | 0.99 | 1.70 | 0.94 | 3.52 | 0.78 | 1.05 | 0.99 | 1.04 | 1.00 | 0.99 | 1.00 |
| FK25 | 0.99 | 1.00 | 0.97 | 1.00 | 1.27 | 0.98 | 1.75 | 0.95 | 1.55 | 0.92 | 0.87 | 0.99 | 0.97 | 1.00 | 0.98 | 1.00 |
| FK26 | 1.02 | 1.00 | 1.03 | 1.00 | 1.27 | 0.98 | 1.57 | 0.93 | 1.42 | 0.87 | 0.96 | 0.99 | 1.05 | 1.00 | 0.99 | 1.00 |
| FK27 | 0.97 | 1.00 | 0.87 | 1.00 | 0.78 | 0.99 | 0.76 | 0.96 | 0.58 | 0.94 | 1.03 | 0.99 | 1.02 | 1.00 | 0.99 | 1.00 |
| FK28 | 1.00 | 1.00 | 1.02 | 1.00 | 1.09 | 0.98 | 1.31 | 0.95 | 2.29 | 0.86 | 0.93 | 0.99 | 1.02 | 1.00 | 0.99 | 1.00 |
| FK29 | 0.95 | 1.00 | 0.96 | 1.00 | 0.93 | 0.99 | 1.02 | 0.92 | 1.03 | 0.87 | 0.95 | 0.99 | 1.06 | 1.00 | 0.99 | 1.00 |
| FK31 | 0.88 | 0.90 | 1.05 | 1.00 | 1.07 | 0.99 | 1.03 | 0.95 | 2.48 | 0.84 | 0.97 | 0.99 | 1.04 | 1.00 | 0.99 | 1.00 |
| FK32 | 1.00 | 1.00 | 1.05 | 1.00 | 0.93 | 0.99 | 1.06 | 0.94 | 1.31 | 0.87 | 1.01 | 0.98 | 1.02 | 1.00 | 0.99 | 1.00 |
| FK34 | 1.00 | 1.00 | 0.96 | 1.00 | 0.83 | 0.99 | 0.56 | 0.92 | 0.92 | 0.89 | 0.83 | 0.99 | 0.99 | 1.00 | 0.99 | 1.00 |
| FK36 | 0.97 | 1.00 | 0.94 | 1.00 | 0.95 | 0.99 | 0.97 | 0.96 | 0.74 | 0.94 | 1.11 | 0.98 | 1.00 | 1.00 | 0.99 | 1.00 |
| FK37 | 0.97 | 1.00 | 1.14 | 1.00 | 1.21 | 0.99 | 0.61 | 0.95 | 0.64 | 0.94 | 0.99 | 0.99 | 1.02 | 1.00 | 0.99 | 1.00 |
| FK38 | 1.08 | 1.00 | 1.06 | 1.00 | 1.03 | 0.99 | 1.25 | 0.95 | 1.02 | 0.92 | 0.98 | 0.99 | 0.99 | 1.00 | 0.99 | 1.00 |
| FK39 | 1.04 | 1.00 | 1.08 | 1.00 | 1.15 | 0.98 | 1.29 | 0.95 | 1.49 | 0.89 | 0.97 | 0.99 | 1.06 | 1.00 | 0.99 | 1.00 |
| KE10 | 1.09 | 1.00 | 1.01 | 1.00 | 0.98 | 0.99 | 1.03 | 0.95 | 1.02 | 0.92 | 0.87 | 0.98 | 0.93 | 1.00 | 0.99 | 1.00 |
| KE11 | 1.03 | 1.00 | 0.93 | 1.00 | 0.75 | 0.99 | 1.13 | 0.94 | 1.23 | 0.88 | 0.86 | 0.99 | 0.99 | 1.00 | 0.98 | 1.00 |
| KE17 | 0.99 | 1.00 | 0.93 | 1.00 | 0.88 | 0.99 | 1.13 | 0.95 | 1.04 | 0.91 | 0.88 | 0.99 | 0.99 | 1.00 | 0.99 | 1.00 |
| KE19 | 1.00 | 1.00 | 1.01 | 1.00 | 1.39 | 0.99 | 1.61 | 0.95 | 2.29 | 0.89 | 0.84 | 0.98 | 0.93 | 1.00 | 0.99 | 1.00 |
| KE21 | 0.95 | 1.00 | 0.92 | 1.00 | 1.07 | 0.99 | 1.44 | 0.94 | 2.24 | 0.86 | 1.04 | 0.97 | 1.13 | 1.00 | 0.99 | 1.00 |
| KE22 | 1.03 | 1.00 | 0.89 | 1.00 | 0.91 | 0.99 | 1.02 | 0.95 | 1.25 | 0.89 | 0.95 | 0.99 | 1.02 | 1.00 | 0.99 | 1.00 |

Table S5 (continued). November 2023 co-location regression results: each monitor (excluding two unacceptable monitors) versus the mean of 55 acceptable monitors

| **Monitor Registered Name** | **0.5_µm_count** | | **1.0_µm_count** | | **2.5_µm_count** | | **5.0_µm_count** | | **10.0_µm_count** | | **temperature** | | **humidity** | | **pressure** | |
| --- | --- | --- | --- | --- | --- | --- | --- | --- | --- | --- | --- | --- | --- | --- | --- | --- |
|  | **Slope** | **R^2^** | **Slope** | **R^2^** | **Slope** | **R^2^** | **Slope** | **R^2^** | **Slope** | **R^2^** | **Slope** | **R^2^** | **Slope** | **R^2^** | **Slope** | **R^2^** |
| KE23 | 0.89 | 1.00 | 1.06 | 1.00 | 1.37 | 0.98 | 1.09 | 0.94 | 0.80 | 0.91 | 1.01 | 0.99 | 0.99 | 1.00 | 0.99 | 1.00 |
| KE24 | 0.99 | 1.00 | 0.97 | 1.00 | 1.07 | 0.99 | 0.94 | 0.95 | 0.85 | 0.92 | 1.00 | 0.99 | 0.98 | 1.00 | 0.99 | 1.00 |
| KE25 | 1.02 | 1.00 | 0.96 | 1.00 | 0.85 | 0.99 | 0.81 | 0.96 | 0.68 | 0.94 | 1.00 | 0.97 | 1.03 | 1.00 | 0.99 | 1.00 |
| KE28 | 0.94 | 0.99 | 1.06 | 1.00 | 0.92 | 0.97 | 1.13 | 0.95 | 0.87 | 0.94 | 0.96 | 0.99 | 1.06 | 1.00 | 0.99 | 1.00 |
| KE29 | 1.02 | 1.00 | 1.09 | 1.00 | 1.22 | 0.99 | 1.92 | 0.93 | 1.58 | 0.91 | 0.91 | 0.99 | 0.98 | 1.00 | 0.99 | 1.00 |
| KE30 | 1.14 | 1.00 | 1.10 | 1.00 | 1.27 | 0.99 | 1.19 | 0.97 | 0.96 | 0.95 | 0.97 | 0.99 | 1.06 | 1.00 | 0.99 | 1.00 |
| KE33 | 0.99 | 1.00 | 0.94 | 1.00 | 0.99 | 0.99 | 1.22 | 0.95 | 1.25 | 0.90 | 0.86 | 0.99 | 0.92 | 1.00 | 0.99 | 1.00 |
| KE34 | 1.09 | 1.00 | 1.05 | 1.00 | 0.98 | 0.99 | 1.02 | 0.96 | 0.92 | 0.93 | 1.00 | 0.99 | 0.99 | 1.00 | 0.99 | 1.00 |
| KE35 | 1.00 | 1.00 | 0.97 | 1.00 | 1.29 | 0.99 | 1.21 | 0.95 | 0.93 | 0.93 | 0.92 | 0.99 | 0.99 | 1.00 | 0.99 | 1.00 |
| KE36 | 1.00 | 1.00 | 0.90 | 1.00 | 0.84 | 0.99 | 1.19 | 0.95 | 1.40 | 0.87 | 0.88 | 0.98 | 0.98 | 1.00 | 0.98 | 1.00 |
| KE37 | 0.85 | 0.99 | 0.97 | 1.00 | 0.94 | 0.99 | 0.49 | 0.91 | 0.37 | 0.83 | 0.97 | 0.99 | 1.01 | 1.00 | 0.99 | 1.00 |
| KE38 | 1.10 | 1.00 | 1.00 | 1.00 | 1.00 | 0.99 | 1.17 | 0.95 | 1.31 | 0.90 | 1.10 | 0.99 | 0.97 | 1.00 | 0.99 | 1.00 |
| KE39 | 1.04 | 1.00 | 0.98 | 1.00 | 1.01 | 0.99 | 1.36 | 0.94 | 1.87 | 0.86 | 0.85 | 0.98 | 0.99 | 1.00 | 0.99 | 1.00 |
| KE40 | 1.09 | 1.00 | 0.98 | 1.00 | 0.86 | 0.99 | 0.92 | 0.95 | 0.75 | 0.92 | 0.88 | 0.99 | 0.98 | 1.00 | 0.99 | 1.00 |
| KE41 | 1.08 | 1.00 | 0.99 | 1.00 | 1.10 | 0.99 | 1.32 | 0.94 | 1.27 | 0.89 | 0.84 | 0.98 | 0.91 | 1.00 | 0.98 | 1.00 |
| KE42 | 0.99 | 1.00 | 0.93 | 1.00 | 0.90 | 0.99 | 1.23 | 0.94 | 1.11 | 0.90 | 0.99 | 0.99 | 1.03 | 1.00 | 0.99 | 1.00 |
| FK10 Outdoor | 0.88 | 0.90 | 1.03 | 1.00 | 1.05 | 0.99 | 0.97 | 0.92 | 0.96 | 0.93 | 0.97 | 0.99 | 0.98 | 1.00 | 0.99 | 1.00 |
| KE29 Outdoor | 0.78 | 0.98 | 1.05 | 1.00 | 0.92 | 0.99 | 0.46 | 0.95 | 0.27 | 0.91 | 0.87 | 0.99 | 0.98 | 1.00 | 0.99 | 1.00 |
| FK12 Outdoor | 0.92 | 1.00 | 1.00 | 1.00 | 0.88 | 0.99 | 0.50 | 0.89 | 1.38 | 0.86 | nan | 0.00 | 0.66 | 0.98 | 0.78 | 0.53 |
| KE22 Outdoor | 0.79 | 1.00 | 0.98 | 1.00 | 1.31 | 0.98 | 1.09 | 0.92 | 0.78 | 0.82 | 0.85 | 0.97 | 0.89 | 1.00 | 0.98 | 1.00 |
| FK29 Outdoor | 0.89 | 1.00 | 1.07 | 1.00 | 1.86 | 0.99 | 1.93 | 0.95 | 2.33 | 0.88 | 0.99 | 0.99 | 0.95 | 1.00 | 0.98 | 1.00 |
| KE40 Outdoor | 0.86 | 1.00 | 0.92 | 1.00 | 0.98 | 0.99 | 0.49 | 0.88 | 0.44 | 0.85 | 1.00 | 0.99 | 0.99 | 1.00 | 0.98 | 1.00 |
| KE30 Outdoor | 0.95 | 1.00 | 0.95 | 1.00 | 0.92 | 0.99 | 0.73 | 0.95 | 0.97 | 0.92 | 0.95 | 0.99 | 1.01 | 1.00 | 0.99 | 1.00 |
| FK38 Outdoor | 0.97 | 1.00 | 0.98 | 1.00 | 0.89 | 0.99 | 0.67 | 0.94 | 0.45 | 0.83 | 1.02 | 0.99 | 0.94 | 1.00 | 0.99 | 1.00 |
| KE37 Outdoor | 0.94 | 1.00 | 1.01 | 1.00 | 0.76 | 0.98 | 0.59 | 0.95 | 0.38 | 0.87 | 0.99 | 0.99 | 1.00 | 1.00 | 0.99 | 1.00 |

# 5. Co-location and Field Data Range Summary

**Table S6** shows the summary of lab co-location data (Lab), field raw data (Field) and field data with co-location factors applied (Field-col) for the 50 monitors (44 indoors and 6 outdoors) that were used in the field study.

Table S6. Summary of the data ranges from lab co-location (“Lab”) and field-collected data, including raw data (“Field”) and data with co-location factors applied (“Field-col”)

| **Parameter** | **Dataset** | **Count** | **Mean** | **Std** | **1%** | **25%** | **Median** | **75%** | **99%** |
| --- | --- | --- | --- | --- | --- | --- | --- | --- | --- |
| PM_2.5__alt  (µg/m^3^) | Lab | 119046 | 3.9 | 3.4 | 0.45 | 1.6 | 2.9 | 5.1 | 16.6 |
|  | Field | 704484 | 8.1 | 21.7 | 0.52 | 2.6 | 4.3 | 7.7 | 69.1 |
|  | Field-col | 704484 | 8.1 | 21.8 | 0.54 | 2.6 | 4.3 | 7.6 | 69.8 |
| PM_1__cf1  (µg/m^3^) | Lab | 119046 | 4.0 | 4.1 | 0 | 1.0 | 2.7 | 5.7 | 19.1 |
|  | Field | 704484 | 8.3 | 20.0 | 0.01 | 2.1 | 4.3 | 8.2 | 79.1 |
|  | Field-col | 704484 | 8.3 | 20.3 | 0.01 | 2.1 | 4.2 | 8.1 | 80.8 |
| PM_2.5__cf1  (µg/m^3^) | Lab | 119046 | 5.9 | 6.0 | 0.06 | 1.7 | 4.1 | 8.2 | 28.9 |
|  | Field | 704484 | 13.1 | 38.9 | 0.12 | 3.5 | 6.6 | 12.4 | 121.4 |
|  | Field-col | 704484 | 13.1 | 38.8 | 0.12 | 3.5 | 6.5 | 12.3 | 122.1 |
| PM_10__cf1  (µg/m^3^) | Lab | 119046 | 6.5 | 6.5 | 0.15 | 2.0 | 4.6 | 8.8 | 32.0 |
|  | Field | 704484 | 14.4 | 50.2 | 0.22 | 4.0 | 7.3 | 13.5 | 130.4 |
|  | Field-col | 704484 | 14.3 | 49.5 | 0.22 | 4.0 | 7.2 | 13.3 | 131.4 |
| PM_1__atm  (µg/m^3^) | Lab | 119046 | 3.9 | 4.0 | 0 | 1.0 | 2.7 | 5.7 | 18.8 |
|  | Field | 704484 | 7.3 | 13.7 | 0.01 | 2.1 | 4.3 | 8.2 | 52.3 |
|  | Field-col | 704484 | 7.3 | 13.8 | 0.01 | 2.1 | 4.2 | 8.1 | 53.4 |
| PM_2.5__atm  (µg/m^3^) | Lab | 119046 | 5.9 | 5.9 | 0.06 | 1.7 | 4.1 | 8.2 | 28.5 |
|  | Field | 704484 | 11.6 | 26.3 | 0.12 | 3.5 | 6.6 | 12.4 | 80.5 |
|  | Field-col | 704484 | 11.5 | 26.2 | 0.12 | 3.5 | 6.5 | 12.2 | 81.1 |
| PM_10__atm  (µg/m^3^) | Lab | 119046 | 6.5 | 6.5 | 0.15 | 2.0 | 4.6 | 8.8 | 31.9 |
|  | Field | 704484 | 12.9 | 34.0 | 0.22 | 4.0 | 7.3 | 13.5 | 88.4 |
|  | Field-col | 704484 | 12.9 | 33.5 | 0.22 | 4.0 | 7.2 | 13.3 | 89.3 |
| 0.3 µm count  (#/dL) | Lab | 119046 | 898.8 | 722.9 | 105.5 | 386.6 | 707.3 | 1200.8 | 645.9 |
|  | Field | 704484 | 1603.3 | 3203.3 | 132.6 | 568.2 | 935.9 | 1609.5 | 13518.8 |
|  | Field-col | 704484 | 1577.0 | 3274.8 | 130.4 | 554.1 | 908.2 | 1558.3 | 13808.6 |
| 0.5 µm count  (#/dL) | Lab | 119046 | 235.8 | 193.9 | 27.4 | 98.4 | 180.9 | 316.8 | 968.7 |
|  | Field | 704484 | 444.3 | 963.0 | 33.7 | 153.7 | 254.1 | 438.8 | 3763.1 |
|  | Field-col | 704484 | 442.3 | 972.7 | 33.7 | 154.3 | 252.4 | 432.5 | 3828.4 |
| 1.0 µm count  (#/dL) | Lab | 119046 | 38.7 | 36.4 | 4.2 | 14.3 | 28.1 | 50.1 | 180.8 |
|  | Field | 704484 | 90.6 | 316.7 | 4.9 | 26.4 | 45.1 | 82.4 | 774.0 |
|  | Field-col | 704484 | 90.2 | 314.5 | 5.0 | 26.5 | 45.0 | 81.9 | 773.5 |
| 2.5 µm count  (#/dL) | Lab | 119046 | 3.6 | 3.9 | 0.39 | 1.4 | 2.5 | 4.3 | 20.9 |
|  | Field | 704484 | 8.7 | 76.6 | 0.43 | 2.3 | 3.9 | 7.1 | 73.5 |
|  | Field-col | 704484 | 8.6 | 74.2 | 0.45 | 2.3 | 3.8 | 7.0 | 73.2 |
| 5 µm  count  (#/dL) | Lab | 119046 | 0.72 | 0.81 | 0.04 | 0.27 | 0.51 | 0.87 | 4.00 |
|  | Field | 704484 | 1.65 | 22.59 | 0.06 | 0.43 | 0.77 | 1.41 | 13.77 |
|  | Field-col | 704484 | 1.52 | 23.28 | 0.06 | 0.43 | 0.75 | 1.29 | 11.99 |
| 10 µm count  (#/dL) | Lab | 119046 | 0.34 | 0.43 | 0 | 0.11 | 0.24 | 0.43 | 1.92 |
|  | Field | 704484 | 0.78 | 9.31 | 0 | 0.19 | 0.38 | 0.7 | 6.97 |
|  | Field-col | 704484 | 0.71 | 9.65 | 0 | 0.2 | 0.37 | 0.64 | 5.64 |

**Figure S7** shows the log-scale box plot of the field PM_2.5__alt data (raw and co-location factor applied) and the lab co-location measurement data from the 50 monitors used in the field study.


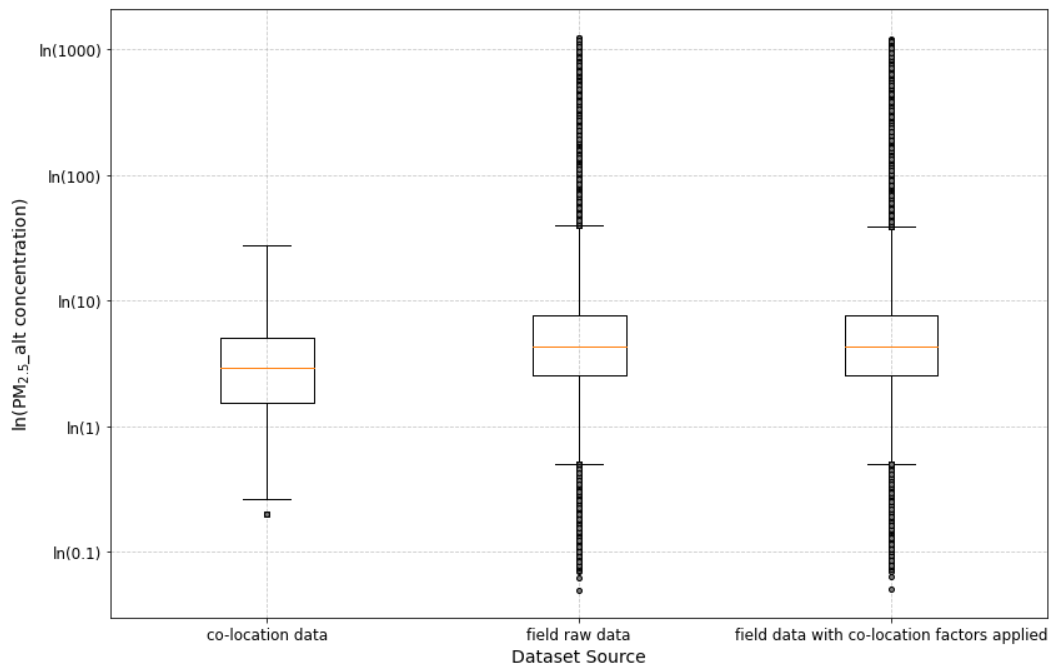


**Figure S7.** Box plot of the field-collected and laboratory co-located PM_2.5__alt concentration data (natural log transformed)


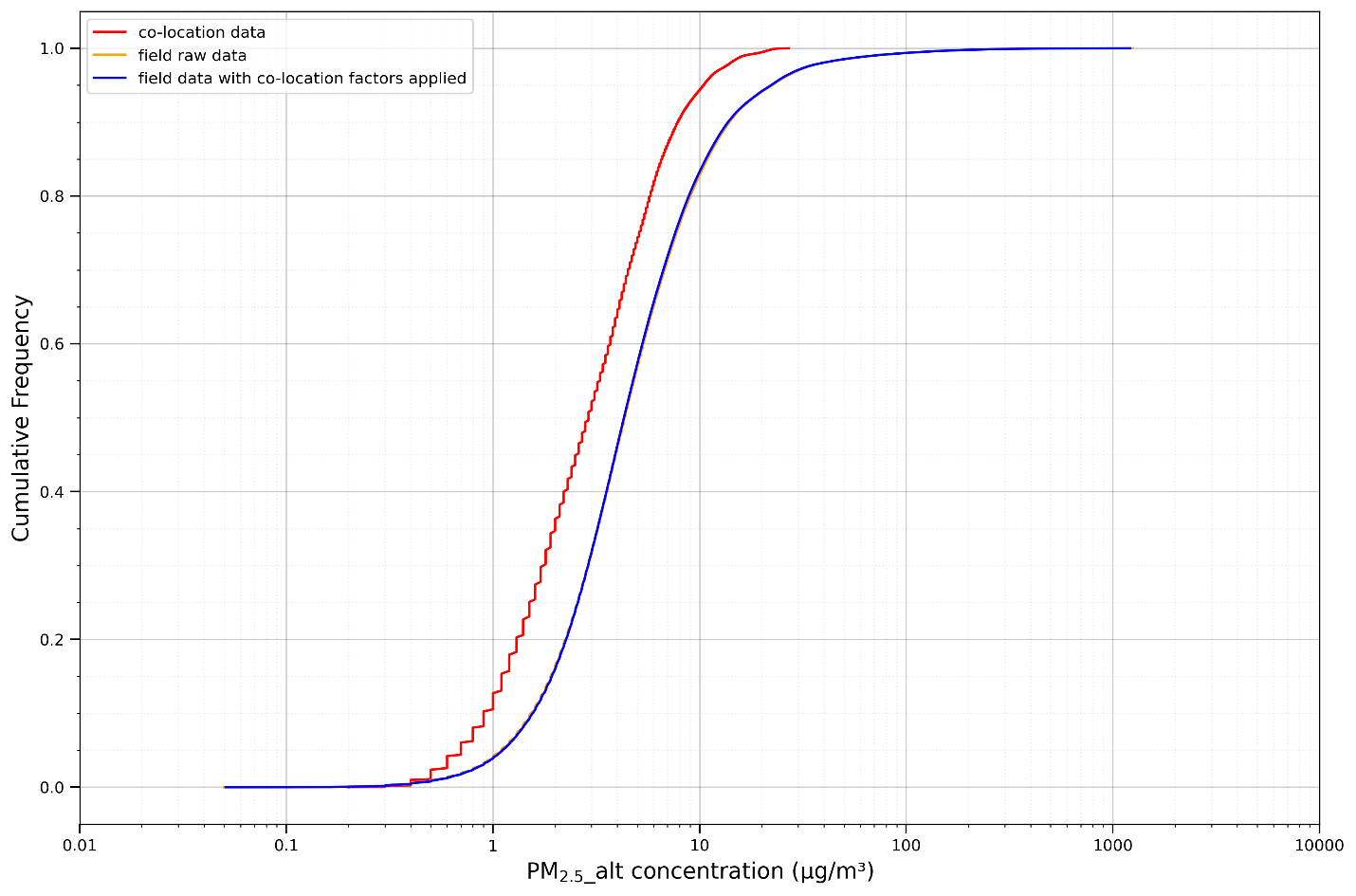


**Figure S8.** Cumulative distributions of the laboratory co-located and field-collected (both raw and co-location factor adjusted) PM_2.5__alt concentration data (natural log transformed for clarity)

The co-location data appeared more normally distributed than the field-collected data. The field raw data and field data with co-location factors show long right tails, suggesting PM_2.5__alt values in these datasets are more concentrated at the lower end. Comparing the field raw data to the field data with co-location factors applied, applying the co-location factors slightly reduced PM_2.5__alt values. Statistical analysis was conducted to examine the differences in PM_2.5__alt concentrations across these three datasets. Firstly, a normality test (Shapiro-Wilk test) was performed on all datasets. The results (p = 0 for all datasets) indicated that none of the datasets were normally distributed, even after logarithmic transformation. Thus, we used a non-parametric statistical test – the Kolmogorov-Smirnov (KS) test – to test for differences in distributions between the datasets. There were statistically significant differences between each data set (p < 0.05); however, the magnitude of these differences was larger between the co-location data and both field datasets (KS statistic ~0.21) compared to the difference between raw and co-location calibrated field data (KS statistic ~0.007). This suggests that while the datasets were statistically different, the co-location calibrated field data maintained very similar distribution characteristics to the raw field data.

**6. Co-location and Field Data Precision Summary**

**Figure S9**, **Table S7**, and **Table S8** summarize the imprecision for 50 monitors for PM_2.5__alt readings between channels a and b for each monitor in both the laboratory co-locations and the field data collection campaigns, comparing the absolute difference between a and b channels and divided by the sum of a and b channels at each collected timestamp.


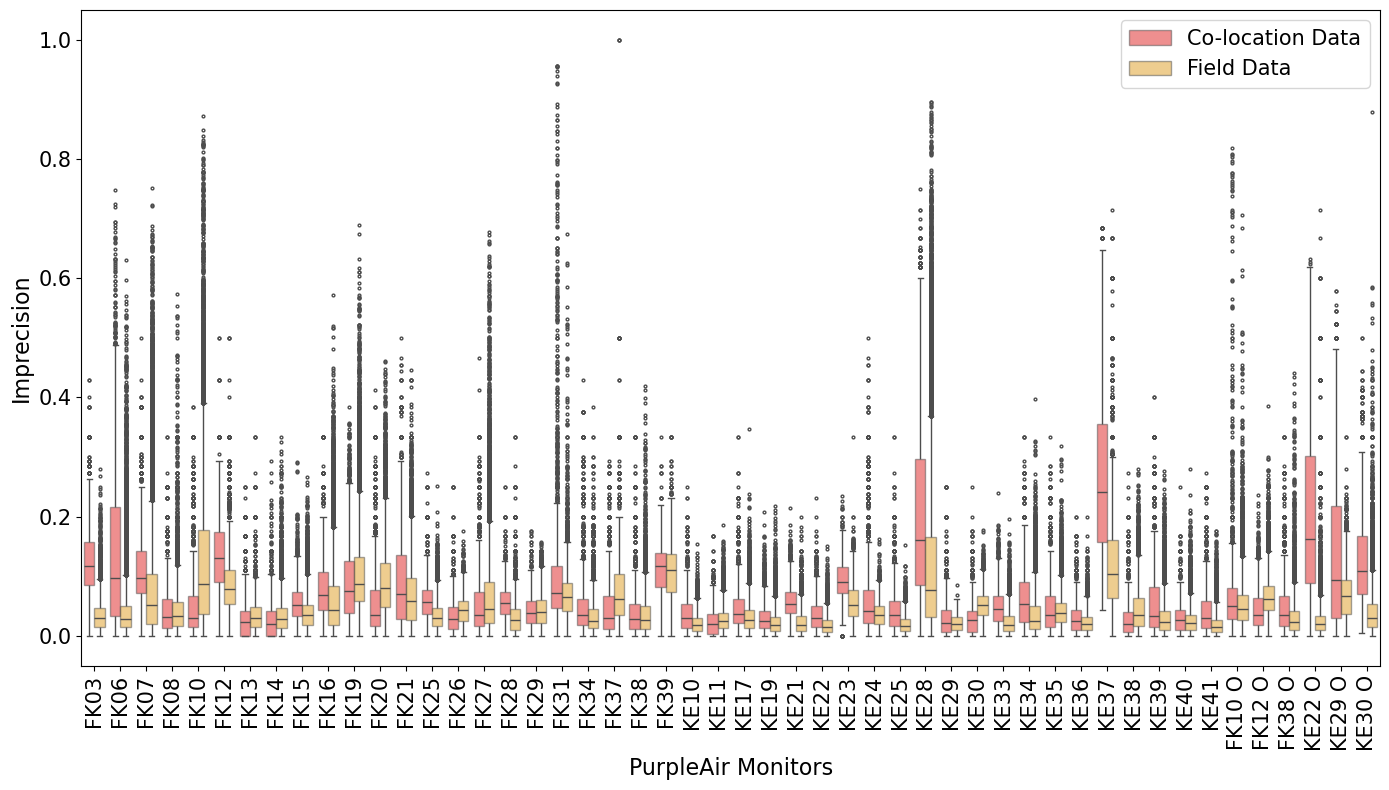


**Figure S9**. Within-monitor imprecision between channel a and b for PM_2.5__alt using both laboratory co-location data and field-collected data

Table S7. Summary of imprecision for 50 monitors on PM_2.5__alt during co-location measurements

| **Monitor Registered Name** | **Valid N** | **Mean** | **SD** | **Lower Quartile (Q1)** | **Median** | **Upper Quartile (Q3)** | **90th Percentile** | |
| --- | --- | --- | --- | --- | --- | --- | --- | --- |
| FK03 | 2418 | 0.13 | 0.06 | 0.09 | 0.12 | 0.16 | 0.20 | |
| FK06 | 2309 | 0.15 | 0.14 | 0.03 | 0.10 | 0.22 | 0.36 | |
| FK07 | 2419 | 0.12 | 0.07 | 0.07 | 0.10 | 0.14 | 0.21 | |
| FK08 | 2456 | 0.04 | 0.05 | 0.01 | 0.03 | 0.06 | 0.11 | |
| FK10 | 2310 | 0.05 | 0.06 | 0.01 | 0.03 | 0.07 | 0.14 | |
| FK12 | 1579 | 0.14 | 0.07 | 0.09 | 0.13 | 0.17 | 0.23 | |
| FK13 | 2418 | 0.03 | 0.03 | 0.00 | 0.02 | 0.04 | 0.07 | |
| FK14 | 2163 | 0.03 | 0.03 | 0.00 | 0.02 | 0.04 | 0.07 | |
| FK15 | 2301 | 0.06 | 0.03 | 0.03 | 0.05 | 0.07 | 0.10 | |
| FK16 | 2308 | 0.08 | 0.05 | 0.04 | 0.07 | 0.11 | 0.16 | |
| FK19 | 2307 | 0.09 | 0.07 | 0.04 | 0.08 | 0.13 | 0.19 | |
| FK20 | 2850 | 0.06 | 0.06 | 0.02 | 0.04 | 0.08 | 0.14 | |
| FK21 | 2419 | 0.10 | 0.09 | 0.03 | 0.07 | 0.14 | 0.22 | |
| FK25 | 2417 | 0.06 | 0.04 | 0.04 | 0.06 | 0.08 | 0.10 | |
| FK26 | 2309 | 0.03 | 0.03 | 0.01 | 0.03 | 0.05 | 0.08 | |
| FK27 | 2309 | 0.06 | 0.06 | 0.02 | 0.04 | 0.07 | 0.14 | |
| FK28 | 2311 | 0.06 | 0.03 | 0.04 | 0.05 | 0.07 | 0.09 | |
| FK29 | 2418 | 0.04 | 0.03 | 0.02 | 0.04 | 0.06 | 0.08 | |
| FK31 | 2418 | 0.11 | 0.14 | 0.05 | 0.07 | 0.12 | 0.23 | |
| FK34 | 2416 | 0.05 | 0.06 | 0.02 | 0.04 | 0.06 | 0.12 | |
| FK37 | 2418 | 0.05 | 0.05 | 0.01 | 0.03 | 0.07 | 0.11 | |
| FK38 | 2307 | 0.04 | 0.05 | 0.01 | 0.03 | 0.05 | 0.10 | |
| FK39 | 2418 | 0.11 | 0.05 | 0.08 | 0.12 | 0.14 | 0.16 | |
| KE10 | 2310 | 0.04 | 0.04 | 0.01 | 0.03 | 0.05 | 0.09 | |
| KE11 | 2306 | 0.03 | 0.02 | 0.00 | 0.02 | 0.04 | 0.06 | |
| KE17 | 2310 | 0.05 | 0.04 | 0.02 | 0.04 | 0.06 | 0.10 | |
| KE19 | 2306 | 0.03 | 0.03 | 0.01 | 0.02 | 0.04 | 0.06 | |
| KE21 | 2308 | 0.06 | 0.03 | 0.04 | 0.05 | 0.07 | 0.09 | |
| KE22 | 2418 | 0.04 | 0.03 | 0.02 | 0.03 | 0.05 | 0.07 | |
| KE23 | 2310 | 0.09 | 0.03 | 0.07 | 0.09 | 0.12 | 0.14 | |
| KE24 | 2305 | 0.07 | 0.07 | 0.02 | 0.04 | 0.08 | 0.17 | |
| KE25 | 2309 | 0.04 | 0.04 | 0.02 | 0.03 | 0.06 | 0.09 | |
| KE28 | 2418 | 0.21 | 0.15 | 0.09 | 0.16 | 0.30 | 0.44 | |
| KE29 | 2309 | 0.03 | 0.03 | 0.01 | 0.02 | 0.04 | 0.07 | |
| KE30 | 2310 | 0.03 | 0.03 | 0.01 | 0.03 | 0.04 | 0.07 | |
| KE33 | 2306 | 0.05 | 0.03 | 0.03 | 0.05 | 0.07 | 0.09 | |
| KE34 | 2305 | 0.06 | 0.05 | 0.02 | 0.05 | 0.09 | 0.14 | |
| KE35 | 2306 | 0.05 | 0.05 | 0.02 | 0.03 | 0.07 | 0.11 | |
| KE36 | 2417 | 0.03 | 0.03 | 0.01 | 0.03 | 0.04 | 0.06 | |
| KE37 | 2309 | 0.27 | 0.14 | 0.16 | 0.24 | 0.35 | 0.47 | |
| KE38 | 2305 | 0.03 | 0.03 | 0.01 | 0.02 | 0.04 | 0.07 | |
| KE39 | 2306 | 0.06 | 0.06 | 0.01 | 0.03 | 0.08 | 0.14 | |
| KE40 | 2417 | 0.03 | 0.03 | 0.01 | 0.03 | 0.04 | 0.06 | |
| KE41 | 2306 | 0.04 | 0.05 | 0.01 | 0.03 | 0.06 | 0.11 |  |

Table S7 (continued). Summary of imprecision for 50 monitors on PM_2.5__alt during co-location measurements

| **Monitor Registered Name** | **Valid N** | **Mean** | **SD** | **Lower Quartile (Q1)** | **Median** | **Upper Quartile (Q3)** | **90th Percentile** |  |
| --- | --- | --- | --- | --- | --- | --- | --- | --- |
| FK10 Outdoor | 2308 | 0.07 | 0.10 | 0.03 | 0.05 | 0.08 | 0.13 | |
| FK12 Outdoor | 2306 | 0.04 | 0.04 | 0.02 | 0.04 | 0.06 | 0.09 | |
| FK38 Outdoor | 2310 | 0.05 | 0.05 | 0.02 | 0.03 | 0.07 | 0.13 | |
| KE22 Outdoor | 2305 | 0.21 | 0.14 | 0.09 | 0.16 | 0.30 | 0.43 | |
| KE29 Outdoor | 2306 | 0.14 | 0.13 | 0.03 | 0.09 | 0.22 | 0.33 | |
| KE30 Outdoor | 2307 | 0.13 | 0.08 | 0.07 | 0.11 | 0.17 | 0.24 | |

Table S8. Summary of imprecision for 50 monitors on PM_2.5__alt during field measurements

| **Monitor Registered Name** | **Valid N** | **Mean** | **SD** | **Lower Quartile (Q1)** | **Median** | **Upper Quartile (Q3)** | **90th Percentile** |
| --- | --- | --- | --- | --- | --- | --- | --- |
| FK03 | 14083 | 0.03 | 0.03 | 0.01 | 0.03 | 0.05 | 0.06 |
| FK06 | 19573 | 0.04 | 0.06 | 0.01 | 0.03 | 0.05 | 0.10 |
| FK07 | 24577 | 0.08 | 0.09 | 0.02 | 0.05 | 0.10 | 0.20 |
| FK08 | 23862 | 0.04 | 0.04 | 0.02 | 0.03 | 0.06 | 0.08 |
| FK10 | 24901 | 0.13 | 0.12 | 0.04 | 0.09 | 0.18 | 0.30 |
| FK12 | 22353 | 0.09 | 0.05 | 0.05 | 0.08 | 0.11 | 0.14 |
| FK13 | 4031 | 0.04 | 0.03 | 0.02 | 0.03 | 0.05 | 0.07 |
| FK14 | 22125 | 0.03 | 0.03 | 0.01 | 0.03 | 0.05 | 0.07 |
| FK15 | 24873 | 0.04 | 0.03 | 0.02 | 0.03 | 0.05 | 0.07 |
| FK16 | 24886 | 0.06 | 0.06 | 0.02 | 0.04 | 0.08 | 0.14 |
| FK19 | 24583 | 0.11 | 0.07 | 0.06 | 0.09 | 0.13 | 0.20 |
| FK20 | 9318 | 0.09 | 0.06 | 0.05 | 0.08 | 0.12 | 0.17 |
| FK21 | 14977 | 0.07 | 0.06 | 0.03 | 0.06 | 0.10 | 0.14 |
| FK25 | 12073 | 0.03 | 0.02 | 0.02 | 0.03 | 0.05 | 0.07 |
| FK26 | 11680 | 0.04 | 0.02 | 0.03 | 0.04 | 0.06 | 0.07 |
| FK27 | 17240 | 0.07 | 0.07 | 0.02 | 0.05 | 0.09 | 0.15 |
| FK28 | 10457 | 0.03 | 0.03 | 0.01 | 0.03 | 0.04 | 0.07 |
| FK29 | 8970 | 0.04 | 0.03 | 0.02 | 0.04 | 0.06 | 0.08 |
| FK31 | 10067 | 0.07 | 0.04 | 0.04 | 0.06 | 0.09 | 0.11 |
| FK34 | 9481 | 0.03 | 0.03 | 0.01 | 0.03 | 0.05 | 0.07 |
| FK37 | 9481 | 0.08 | 0.06 | 0.03 | 0.06 | 0.10 | 0.15 |
| FK38 | 5469 | 0.04 | 0.04 | 0.01 | 0.03 | 0.05 | 0.09 |
| FK39 | 3749 | 0.10 | 0.05 | 0.07 | 0.11 | 0.14 | 0.17 |
| KE10 | 5474 | 0.02 | 0.02 | 0.01 | 0.02 | 0.03 | 0.05 |
| KE11 | 12473 | 0.03 | 0.02 | 0.01 | 0.02 | 0.04 | 0.05 |
| KE17 | 21295 | 0.03 | 0.02 | 0.01 | 0.03 | 0.04 | 0.06 |
| KE19 | 17594 | 0.02 | 0.02 | 0.01 | 0.02 | 0.03 | 0.05 |
| KE21 | 6697 | 0.02 | 0.02 | 0.01 | 0.02 | 0.03 | 0.05 |
| KE22 | 11761 | 0.02 | 0.02 | 0.01 | 0.01 | 0.03 | 0.04 |
| KE23 | 5484 | 0.06 | 0.03 | 0.03 | 0.05 | 0.08 | 0.10 |
| KE24 | 17723 | 0.04 | 0.02 | 0.02 | 0.03 | 0.05 | 0.06 |

Table S8 (continued). Summary of imprecision for 50 monitors on PM_2.5__alt during field measurements

| **Monitor Registered Name** | **Valid N** | **Mean** | **SD** | **Lower Quartile (Q1)** | **Median** | **Upper Quartile (Q3)** | **90th Percentile** |
| --- | --- | --- | --- | --- | --- | --- | --- |
| KE25 | 7606 | 0.02 | 0.02 | 0.01 | 0.02 | 0.03 | 0.04 |
| KE28 | 19220 | 0.13 | 0.14 | 0.03 | 0.08 | 0.17 | 0.32 |
| KE29 | 169 | 0.02 | 0.02 | 0.01 | 0.02 | 0.03 | 0.05 |
| KE30 | 22021 | 0.05 | 0.02 | 0.04 | 0.05 | 0.07 | 0.08 |
| KE33 | 6696 | 0.02 | 0.02 | 0.01 | 0.02 | 0.03 | 0.05 |
| KE34 | 13938 | 0.04 | 0.04 | 0.01 | 0.03 | 0.05 | 0.08 |
| KE35 | 13897 | 0.04 | 0.03 | 0.02 | 0.04 | 0.05 | 0.08 |
| KE36 | 8464 | 0.02 | 0.02 | 0.01 | 0.02 | 0.03 | 0.05 |
| KE37 | 9944 | 0.13 | 0.09 | 0.06 | 0.10 | 0.16 | 0.24 |
| KE38 | 5261 | 0.05 | 0.04 | 0.02 | 0.03 | 0.06 | 0.10 |
| KE39 | 12799 | 0.03 | 0.03 | 0.01 | 0.02 | 0.04 | 0.07 |
| KE40 | 12420 | 0.02 | 0.02 | 0.01 | 0.02 | 0.03 | 0.05 |
| KE41 | 5091 | 0.02 | 0.02 | 0.01 | 0.01 | 0.03 | 0.04 |
| FK10 Outdoor | 21281 | 0.05 | 0.04 | 0.03 | 0.05 | 0.07 | 0.10 |
| FK12 Outdoor | 23061 | 0.07 | 0.03 | 0.04 | 0.06 | 0.08 | 0.11 |
| FK38 Outdoor | 5746 | 0.03 | 0.04 | 0.01 | 0.02 | 0.04 | 0.07 |
| KE22 Outdoor | 22689 | 0.03 | 0.04 | 0.01 | 0.02 | 0.03 | 0.05 |
| KE29 Outdoor | 21365 | 0.07 | 0.04 | 0.04 | 0.07 | 0.09 | 0.12 |
| KE30 Outdoor | 21506 | 0.04 | 0.04 | 0.01 | 0.03 | 0.05 | 0.08 |

**Supplemental References**

Solomon, G., Martinez, N., Von Behren, J., Kaser, I., Chang, D., Singh, A., Jarmul, S., Miller, S., Reynolds, P., Heidarinejad, M., Stephens, B., Singer, B., Wagner, J., Balmes, J., 2025. Evaporative Coolers and Wildfire Smoke Exposure: A Climate Justice Issue in Hot, Dry Regions Authors. Frontiers in Public Health 13. https://doi.org/10.3389/fpubh.2025.1541053

1. <https://community.purpleair.com/t/api-pricing/4523> [↑](#footnote-ref-2)
